# 1,1′-Bi-2,2′-naphthol-Based Macrocycles as Dual Metal Cation Chemosensors: Synthesis, Spectroscopic Characterization, and Molecular Dynamics Insights

**DOI:** 10.1021/acs.orglett.6c02303

**Published:** 2026-08-05

**Authors:** Michele Larocca, Michela Buonocore, Davide Alfredo Meo, Manuela Grimaldi, Anna Maria D’Ursi, Annunziata Soriente

**Affiliations:** † Department of Chemistry and Biology, 19028 University of Salerno, Via Giovanni Paolo II, 132-84084 Fisciano, Salerno, Italy; ‡ Department of Chemical Sciences, University of Naples “Federico II”, Strada Comunale Cintia, 80126 Naples, Italy; § Department of Pharmacy, 19028 University of Salerno, Via Giovanni Paolo II, 132-84084 Fisciano, Salerno, Italy

## Abstract

This study reports the synthesis and characterization of two target macrocycles (**5** and **10**) intended to serve as chemosensors for metal cations relevant to biological processes. According to our findings, macrocycle **5** exhibited a marked fluorescence response toward Ag^+^ and Co^2+^, exhibiting 75.2% fluorescence quenching with Ag^+^ and fluorescence enhancement with Co^2+^ (73.5%). Comprehensive NMR studies provided in-depth insight into the binding mechanisms between the macrocycles and the investigated cations. The analysis revealed that the interaction with Ag^+^ is strongly influenced by conformational rearrangements of the macrocyclic framework in three-dimensional space, suggesting a dynamic adaptation of the host structure to optimize the coordination. Overall, these results indicate that macrocycle **5** can act as an effective dual chemosensor for metal cations, capable of discriminating between different species through distinct fluorescence responses, and thus represents a promising platform for the development of advanced sensing systems in biologically relevant environments.

## Graphical abstract

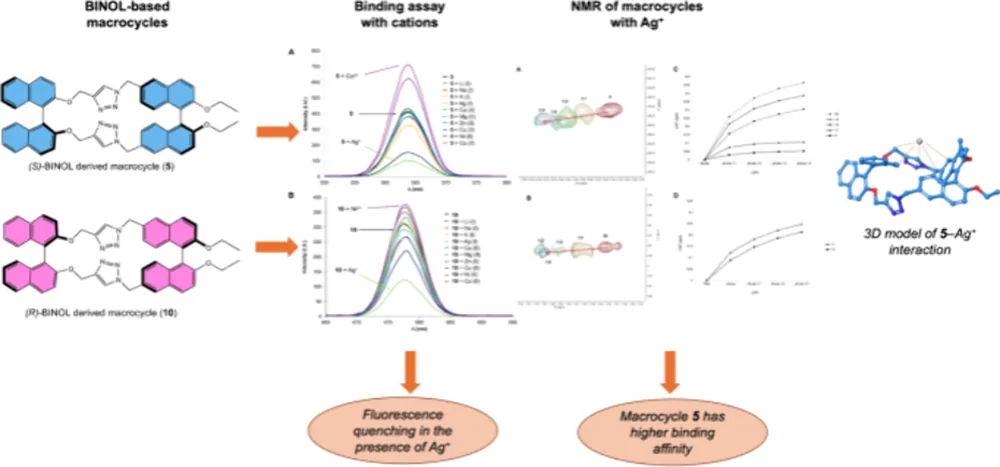

Metal ions are essential elements for the biological functions of living beings, as they are involved in structural and functional roles in many processes. However, when administered at inappropriate concentrations, they may cause harmful effects on human health. Therefore, detecting metal ions in food, soil, and water is crucial.^1–3^ So far, the detection of metal ions has mainly relied on sophisticated analytical techniques, such as AAS, XFS, AES, and ICPMS, but several disadvantages, like high costs, limited portability, and time-consuming sample manipulation, remain issues to be solved.^1,4^ Compared to the aforementioned analytical methods, molecular chemosensors exhibit several advantages since they can provide the use of miniaturized instruments, fast acquisition, low costs, and simple manipulation.^1,5–7^ For these reasons, the interest in developing chemosensors dedicated to metal sensing is increasing. In this context, 1,1′-bi-2,2′-naphthol (BINOL) plays a key role as a class of important chiral molecules with extensive applications in molecular recognition.^8–10^ A BINOL molecule has unique structural features, ranging from a stable and commercially available chiral configuration [(*R*)- and (*S*)-BINOL pure enantiomers] to a highly tunable structure, since functional groups can be specifically introduced to the 2, 3, 4, 5, and 6 positions of the enantiomerically pure BINOL units, modulating even its fluorescent properties.^10,11^ Various studies have described BINOL units and their derivatives as effective molecular tools for detecting metal cations; examples include the detection of Fe^3+^, Bi^3+^, and Ag^+^.^12–14^ Silver cations (Ag^+^) have attracted significant attention for their negative environmental and health impacts, as they can bind various metabolites, such as carboxyl groups, and inactivate sulfhydryl enzymes.^14^ To date, various fluorescent sensors have been developed to detect metal ions, but compared to other heavy transition metal ions, only a few detect silver ions.^3,14,15^ Concerning the latter considerations, we focused on developing a chemosensor based on BINOL units with novel molecular properties, capable of sensing one or more metal cations by displaying specific fluorescence profiles. Recently, we have synthesized two new BINOL-based macrocycles exploiting copper­(I)-catalyzed azide–alkyne cycloaddition (CuAAC) (Figure 1).^16–19^ The macrocycles were then fully characterized, and their ability to complex metal cations of biological interest was tested by fluorescence spectroscopy and NMR titrations. The competitively higher aromaticity of a 1,2,3-triazole ring with respect to benzene, pyridine, pyrrole, pyrazole, and imidazole makes it an electron-rich site that provides binding of metal ions, thereby rendering it as pivotal for the design and synthesis of 1,2,3-triazole-based chemosensors,^20^ considering that the triazole products are not just passive linkers but exhibit bioactivity as able to associate with biological targets.^21^

**Figure 1:**
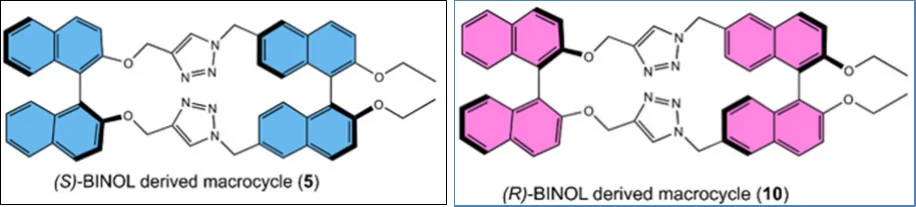
Final macrocycle products.

## Experimental Section

General methods: structural assignments were made with additional information from gCOSY, gHSQC, gNOESY, and gHMBC experiments. The chemosensors were appropriately designed to have an asymmetric structure including two different BINOL units, with the one on the outer side of the molecule having the hydroxyl groups protected by short alkyl chains to prevent the aspecific binding of the metal cations at the outer side of the molecule. Synthetic procedures are reported in detail in the Supporting Information.

Figures 2 and 3 report the synthetic schemes of macrocycles **5** and **10**. Precursors were synthesized in reasonably high or good yields and fully characterized prior to CuAAc macrocyclization. Once obtained, target macrocycles **5** and **10** were characterized by UV/vis spectroscopy, fluorescence, circular dichroism (CD), nuclear magnetic resonance (NMR), and molecular dynamics (MD) simulations. From UV/vis spectroscopy, we observed that the two macrocycles exhibit distinct absorption profiles, with maxima at 281 nm for compound **5** and 338 nm for compound **10** (Figure 4).

**Figure 2:**
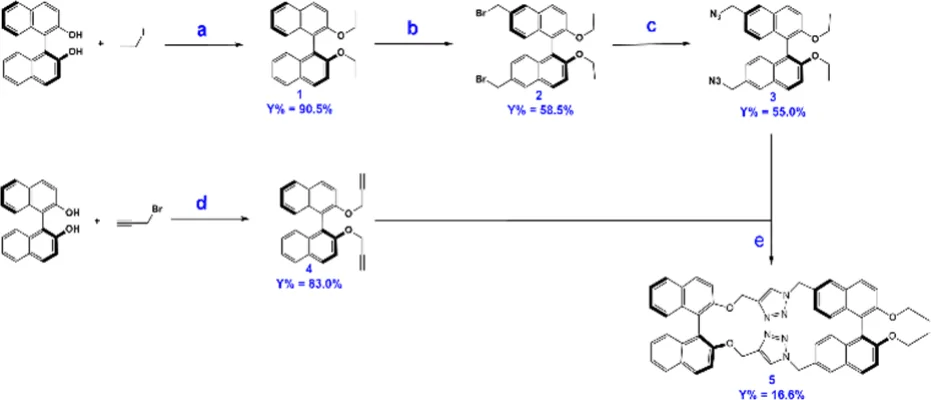
Procedures for the synthesis of macrocycle **5** and its precursors **1**–**4** (details in the Supporting Information).

**Figure 3:**
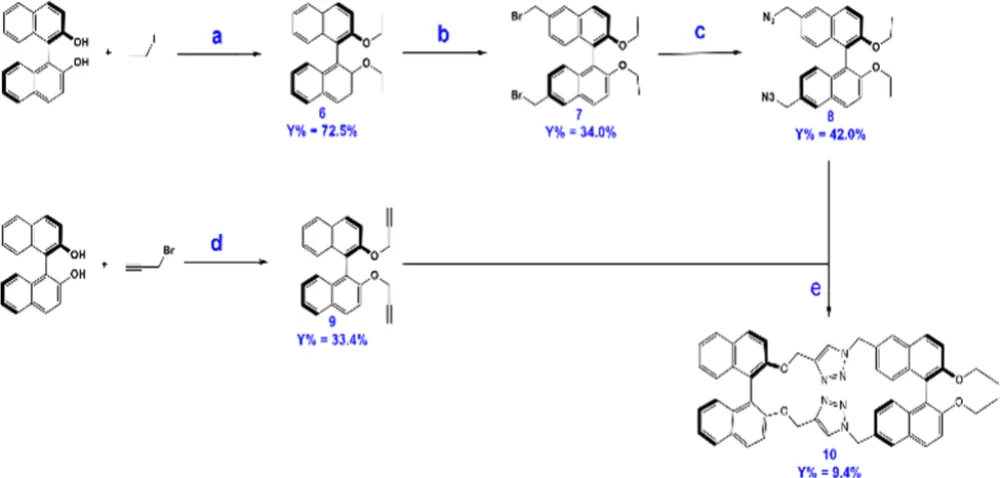
Procedures for the synthesis of macrocycle **10** and its precursors **6**–**9** (details in the Supporting Information).

**Figure 4:**
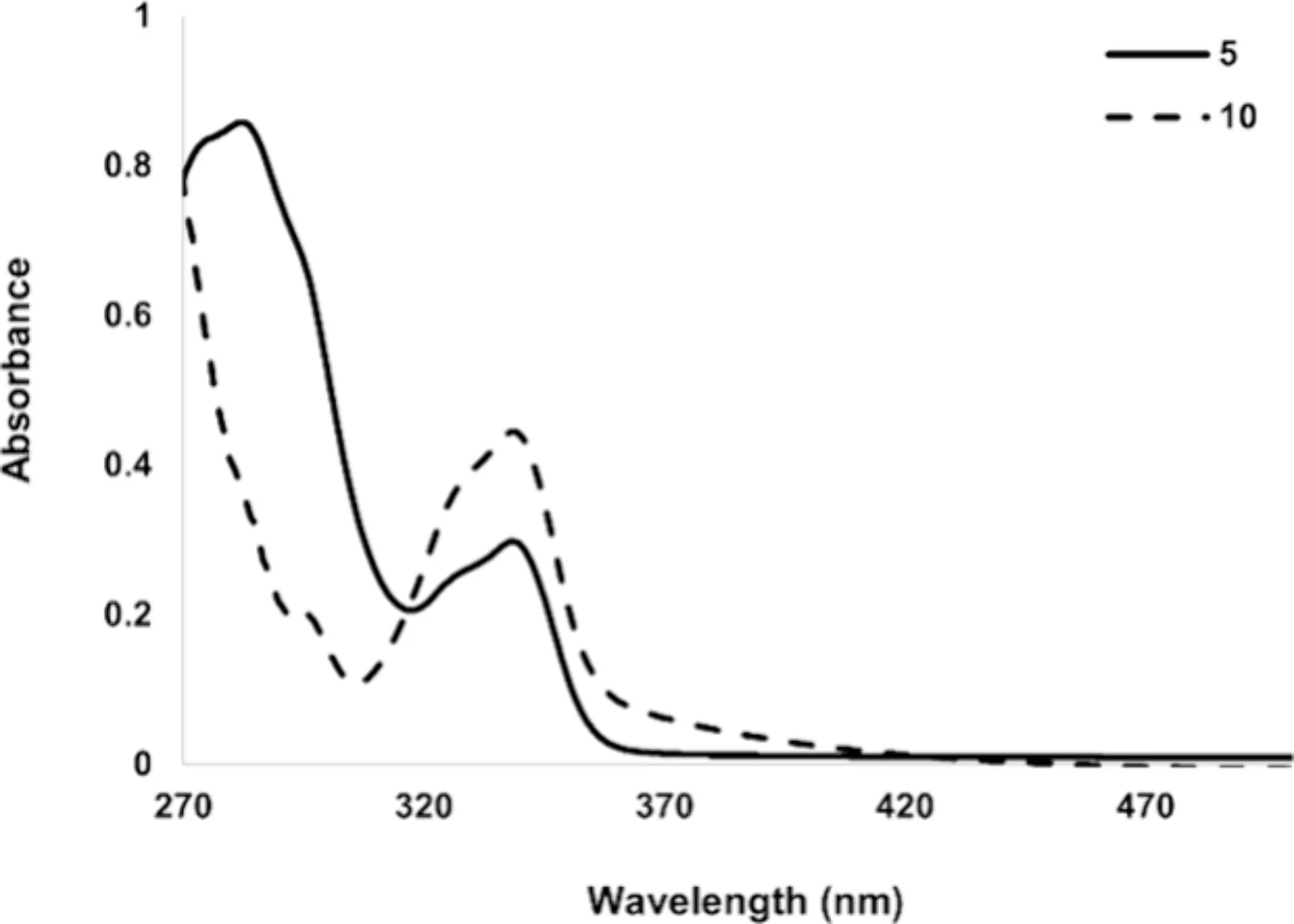
UV/vis spectra of macrocycles **5** and **10**.

Since both spectra were acquired under the same conditions, these absorption profiles might suggest the presence of different conformations, as further confirmed by NMR and MD simulations reported afterward (Figure 8), where, in our opinion, this output is strictly dependent on the different supramolecular properties of each macrocycle.

Fluorescence studies were performed to preliminarily investigate interactions with several metal cations. The results (Figure 5) show that macrocycle **5** exhibits fluorescence quenching (75.2%) in the presence of Ag^+^ and fluorescence enhancement in the presence of Co^2+^ (73.5%). However, Cu^2+^ (62.4% quenching) and Ni^2+^ (52.1% enhancement) also exhibit a good response (Figure 5A). Macrocycle **10** exhibits lower selectivity and less pronounced responses (Figure 5B): fluorescence quenching was observed with Ag^+^ (60.5%), Cu^2+^ (27.9%), and Zn^2+^ (13.4%), whereas enhancement was observed with Ni^2+^ (22.5%), Li^+^ (19.1%), and Co^2+^ (13.3%). The relative fluorescence quantum yield was calculated by comparing the macrocycles in the presence and absence of metal ion, using the ratio of the integrated emission spectra areas and correcting for absorbance at the excitation wavelength from eq 1.

1 $$\frac{\phi_{\mathrm{metal}}}{\phi_{\mathrm{free}}}=\left(\frac{I_{\mathrm{metal}}}{I_{\mathrm{free}}}\right)\cdot \left(\frac{A_{\mathrm{free}}}{A_{\mathrm{metal}}}\right)$$

The results, reported in Table S5, indicate that values of Φ_metal_/Φ_free_ > 1 correspond to fluorescence enhancement, whereas values of <1 indicate quenching induced by the metal. These values represent relative changes in the quantum yield used to compare sensing responses toward different metal ions under identical experimental conditions. Therefore, Φ_metal_/Φ_free_ is a convenient descriptor of the macrocycle sensing performance, although it is not an absolute fluorescence quantum yield measurement.

**Figure 5:**
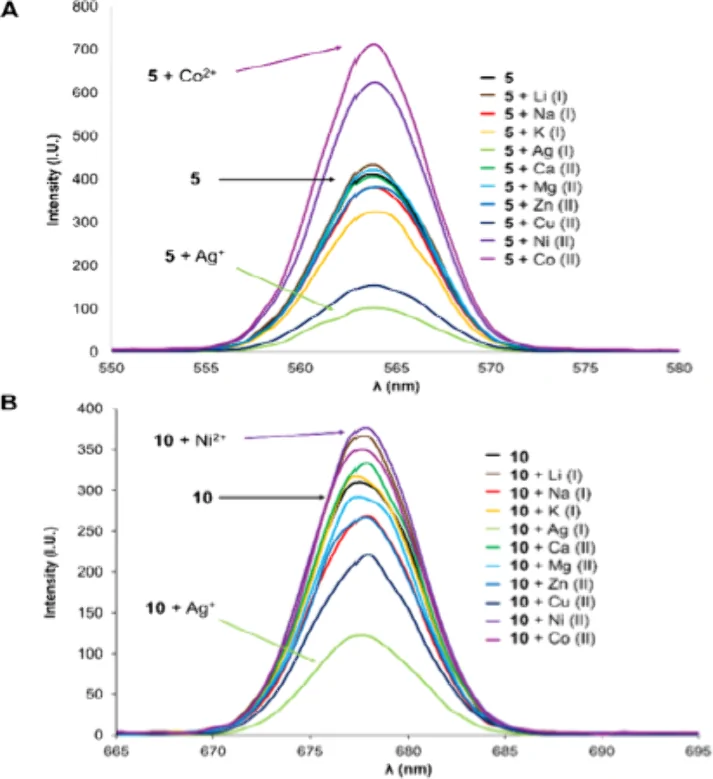
Fluorescence spectra of the two macrocycles (A) **5** and (B) **10** at 11 μM in 99:1, v/v, MeOH/DMSO (black lines).

CD analysis reports two different curves for the enantiomers: macrocycle **5**, deriving from (*S*)-BINOL, shows an intense positive CD effect at 247 nm, while macrocycle **10**, deriving from (*R*)-BINOL, has a negative CD effect at the same wavelength, thus indicating an enantiomeric purity of the two products (Figure S70 of the Supporting Information). NMR spectroscopy was employed to characterize the macrocycles in deuterated dimethyl sulfoxide (DMSO-*d*_6_). We solved the NMR structures of compounds **5** and **10** in DMSO-*d*_6_, acquiring bidimensional NMR experiments and assigning all the corresponding ^1^H and ^13^C chemical shifts (see the Supporting Information).^22^ The 2D ^1^H–^13^C HSQC NMR spectrum shown in Figure 6 provides chemical shift assignments for the proton and carbon signals of both enantiomers. Due to the macrocycle’s symmetry, we report the assignment of a half molecule, highlighted in the following figure.

**Figure 6:**
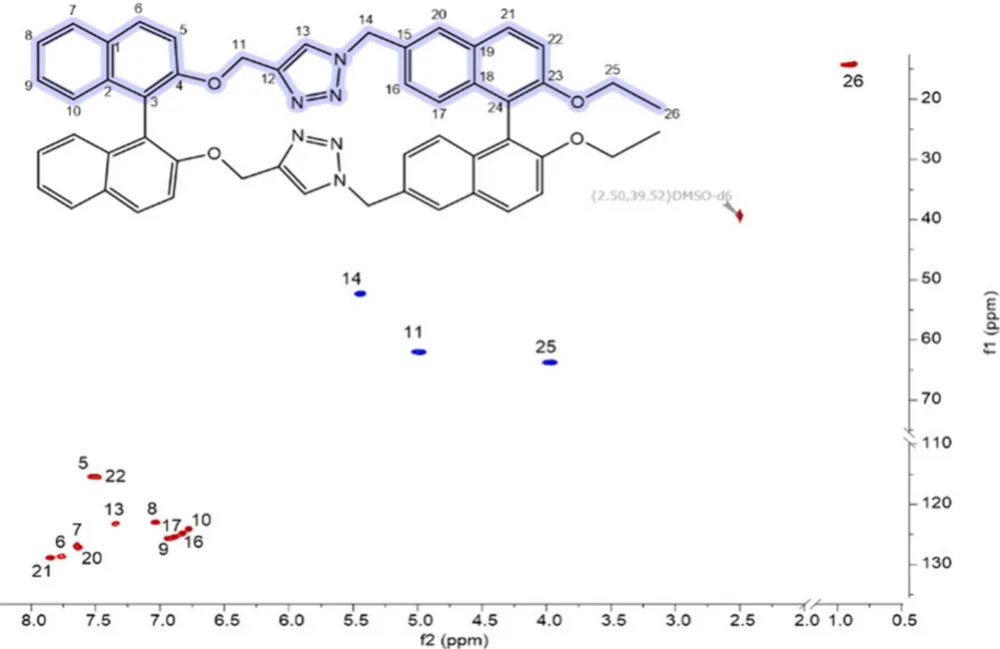
2D ^1^H–^13^C HSQC NMR spectrum of the macrocycles in DMSO-*d*_6_.

On the basis of the 2D ^1^H–^1^H NOESY experiments and the integration of NOE effects, the 3D conformations of compounds **5** and **10** were elucidated. These conformations were subsequently refined and energy-minimized through MD simulations. The resulting models, as shown in Figure 7, reveal a significant conformational difference between the two macrocycles. The structure derived from (*S*)-BINOL adopts a more compact conformation, while the macrocycle derived from (*R*)-BINOL displays a more open conformation with the BINOL-derived moiety oriented outward, away from the macrocyclic cavity.

**Figure 7:**
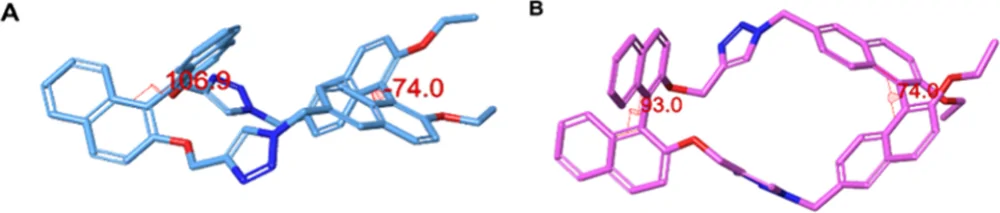
3D conformations of (A) **5** and (B) **10**. Dihedral angles are reported in red.

To identify the functional groups involved in the complex formation with AgNO_3_ and CoCl_2_, we carried out an extensive NMR analysis of the two macrocycles **5** and **10** in the non-complexed state as well as after the addition of various amounts of AgNO_3_ and CoCl_2_. Chemical shift perturbation (CSP) analysis was performed to investigate interactions between the aforementioned metal ions and compounds **5** and **10**. ^1^H–^13^C HSQC spectra of **5** and **10** in the absence and presence of increasing concentrations of metals were acquired up to a 1:4 equiv ratio. Figures 8 and 9 report the results of the CSP analysis^23^ performed by using the following eq 2:

2 $${\Delta \delta }_{\mathrm{comb}}=\sqrt{{\left(\Delta \delta H\right)}^{2}+{\left(0.2514\times \Delta \delta C\right)}^{2}}$$

where Δδ_comb_ is the weighted Euclidean distances expressed as chemical shift differences in either ^1^H and ^13^C dimensions, ΔδH is the chemical shift observed for protons, ΔδC is the chemical shift observed for carbon, and 0.2514 is a weighting factor balance set to |γC|/|γH| = 10.705/42.58 = 0.2514, where γ is a constant (expressed in MHz/T) existing for each type of nuclei.

**Figure 8:**
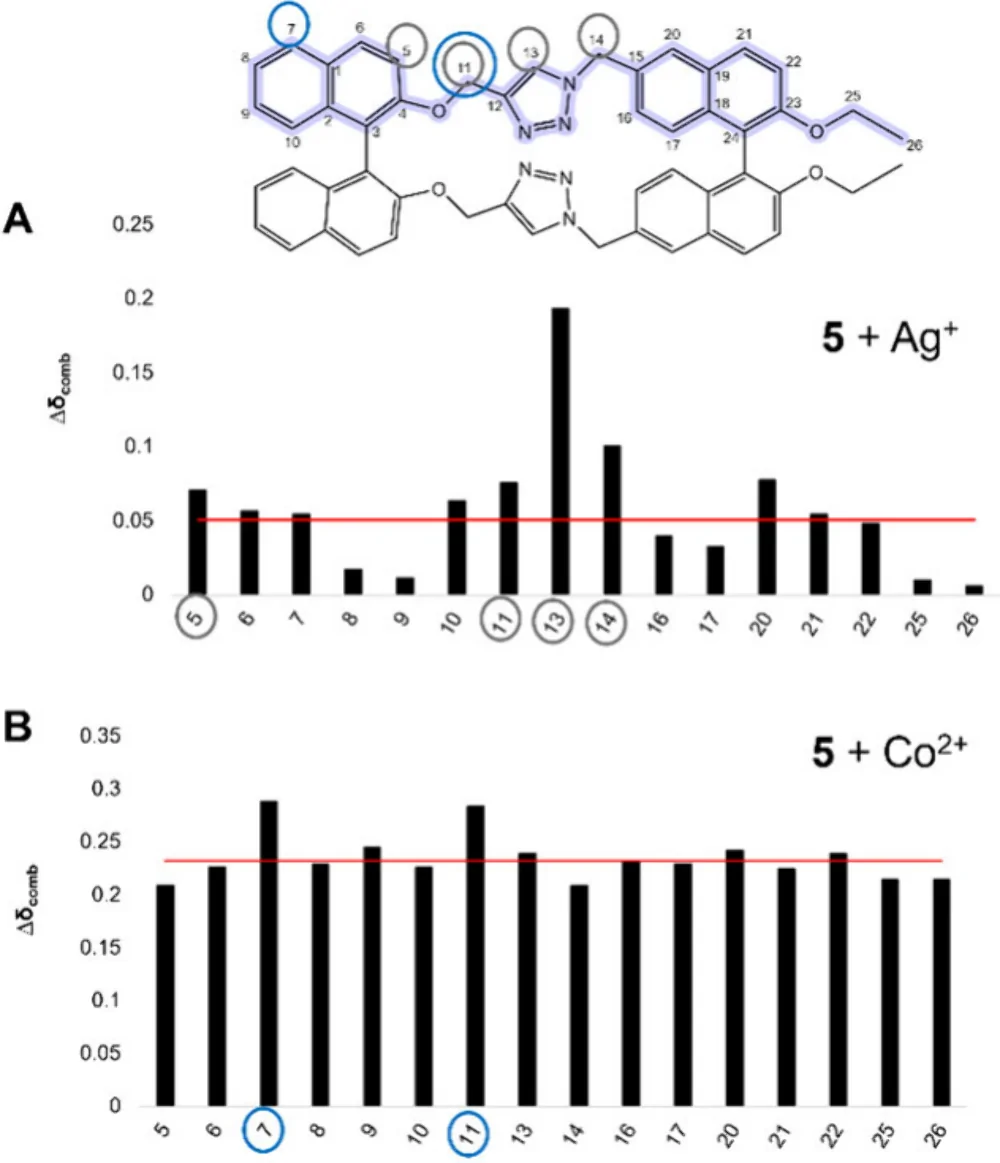
Chemical shift perturbation of the (A) **5**/AgNO_3_ complex in the molar ratio of 1:4 vs non-complexed **5** and (B) **5**/CoCl_2_ complex in the molar ratio of 1:4 vs non-complexed **5**. The red lines represent the average CSPs, whereas (*S*)-BINOL refers to (*S*)-BINOL-based macrocycle **5** (for the numbering, see the Supporting Information). Residues marked in gray are most affected in the assay with Ag^+^, while those marked in blue are most affected in the assay with Co^2+^.

**Figure 9:**
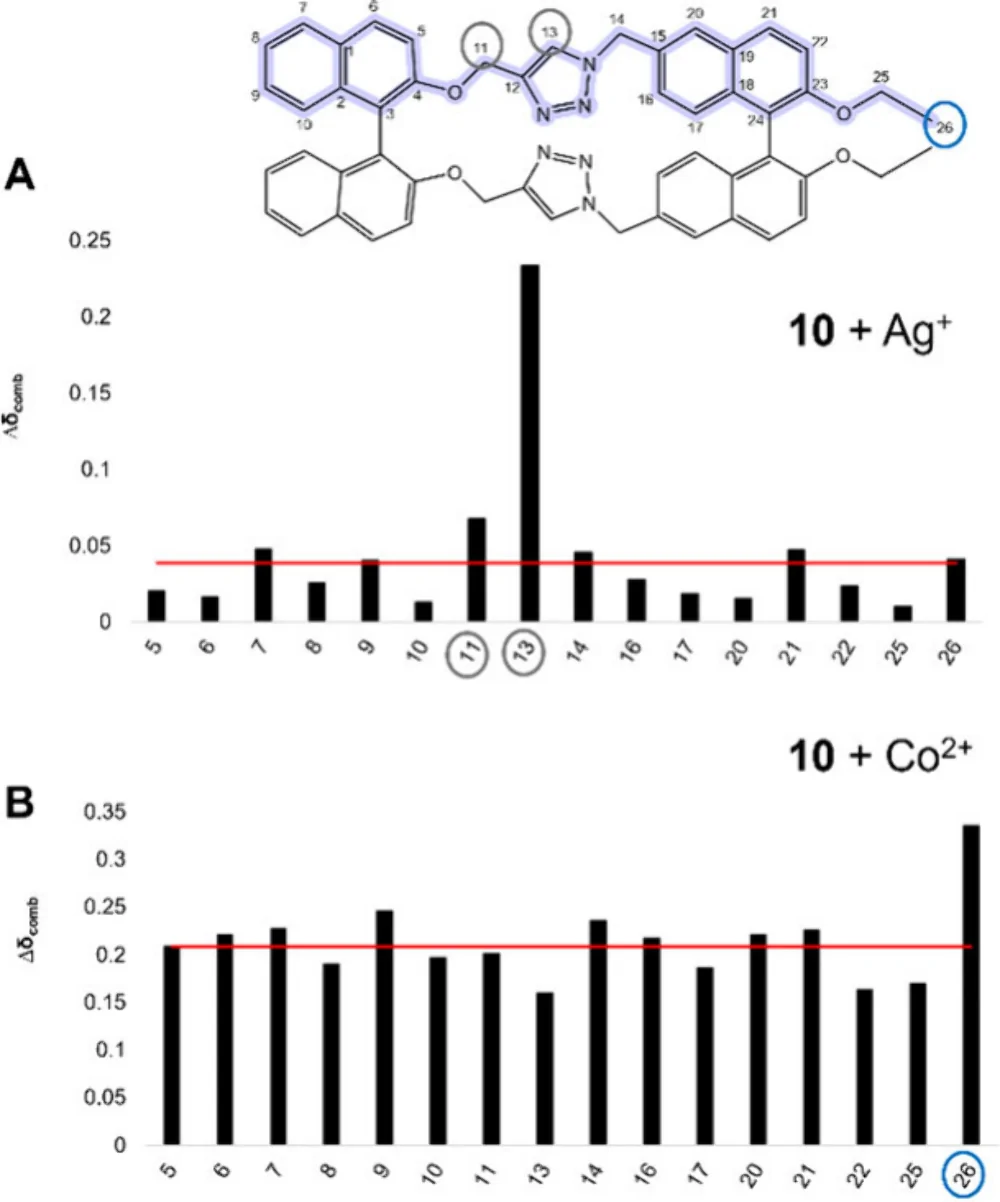
Chemical shift perturbation of the (A) **10**/AgNO_3_ complex in the molar ratio of 1:4 vs non-complexed **10** and (B) **10**/CoCl_2_ complex in the molar ratio of 1:4 vs non-complexed **10**. The red lines represent the average CSPs, whereas (*R*)-BINOL refers to (*R*)-BINOL-based macrocycle **10** (for numbering, see the Supporting Information). Residues marked in gray are most affected in the assay with Ag^+^, while those marked in blue are most affected in the assay with Co^2+^.

Therefore, ΔδH and ΔδC were calculated for compound **5** in the presence of AgNO_3_ and CoCl_2_ and plotted in a bar chart in Figure 8A and B, respectively, and ΔδH and ΔδC were calculated for compound **10** in the presence of AgNO_3_ and CoCl_2_ and represented in a bar graph in Figure 9A and B, respectively.

In Figure 8A, the addition of 4 equiv of AgNO_3_ leads to a change in the conformation mainly involving the signal related to the atoms 5, 11, 13, 14, and 20, whereas from Figure 8B, the addition of 4 equiv of CoCl_2_ results in the perturbation of all the atoms with a focus on 7 and 11.

Accordingly, Figure 9 reports the results of CSP analysis of compound **10** in the presence of AgNO_3_ and CoCl_2_. Figure 9A shows how the addition of 4 equiv of AgNO_3_ leads to a change in conformation mainly involving atoms 11 and 13, whereas Figure 9B illustrates how the addition of 4 equiv of CoCl_2_ results in the perturbation of all atoms, with a focus on atom 26.

Concerning the NMR titrations of both compounds **5** and **10** in the presence of AgNO_3_ (0–4 equiv), a relevant shift of the CH triazole signals was observed in both cases (Figure 10A and B), suggesting a direct involvement of the triazole rings in the interaction with Ag^+^. In the presence of CoCl_2_, broad chemical shift variations were observed throughout the macrocyclic framework. Considering the paramagnetic nature of Co^2+^, these perturbations are likely dominated by paramagnetic effects rather than by the formation of a well-defined coordination complex. Therefore, unlike Ag^+^, for which specific binding interactions could be identified, the NMR data do not allow an unambiguous characterization of a specific Co^2+^-binding mode.

**Figure 10:**
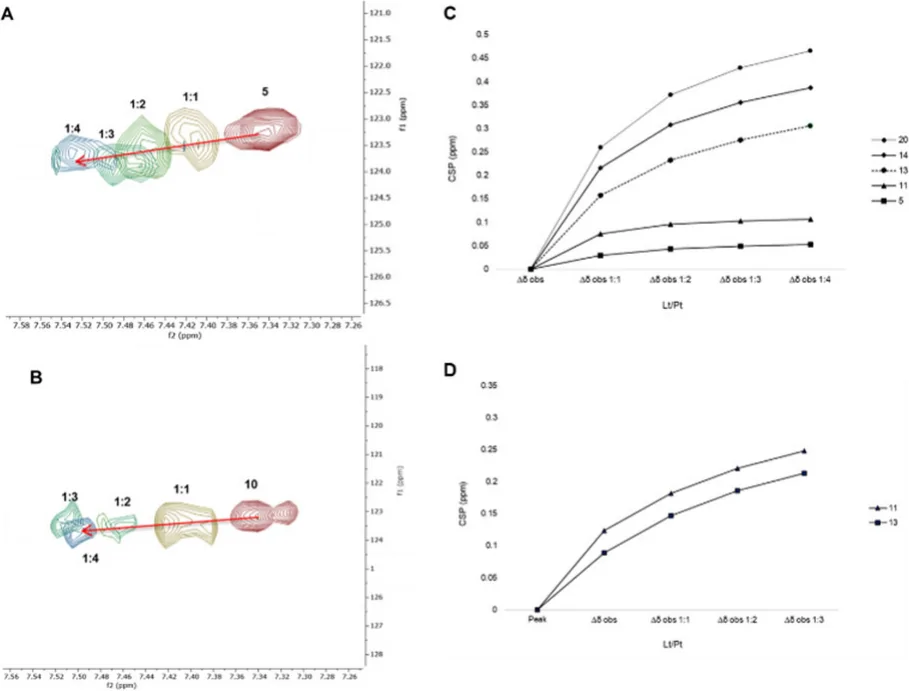
Chemical shift perturbation of atom 13 in (A) macrocycle **5** and (B) macrocycle **10** at macrocycle/AgNO_3_ molar ratios of 1:1 (yellow peak), 1:2 (light green peak), 1:3 (dark green peak), and 1:4 (blue peak) vs non-complexed macrocycle (red peak). Chemical shift perturbation curves of atoms (C) 5–11–13–14–20 of compound **5** and (D) 11–13 of compound **10**.

Taken together, the NMR titration profiles indicate that the most significant chemical shift perturbations occur within the first equivalent of Ag^+^ added, followed by a tendency toward saturation at higher metal concentrations. Although not constituting a definitive stoichiometric determination, this behavior is consistent with a predominant 1:1 interaction between macrocycle **5** and Ag^+^.^24^ The interaction involves more residues in compound **5**, suggesting a different arrangement of the triazole rings compared to that of compound **10**. This hypothesis was further investigated by acquiring 2D NOESY^25^ spectra in the absence and presence of metal ions and performing MD simulations. Intra- and interresidual NOE effects were analyzed to investigate the structure of the macrocycles moving from the non-complexed state to the 1:4 complex (details in the Supporting Information). Figure 11 shows a model of the interaction between **5** and the Ag^+^ cation, derived from NMR chemical shift perturbation information, NOESY calculations, which enables the visualization of the conformation assumed by the macrocycle, and MD simulations. Quantitative metrics for MD simulations of macrocycle **5** in complex with Ag^+^ compared to those of the free macrocycle are reported in Figure S66. The dynamic analysis shows that the free macrocycle displays higher energy values and larger fluctuations, while coordination with Ag^+^ leads to lower and more stable profiles, suggesting that metal binding rigidifies the macrocycle and promotes a more compact ordered conformation. Overall, the Ag^+^ complex appears to be dynamically more stable than the free ligand.

**Figure 11:**
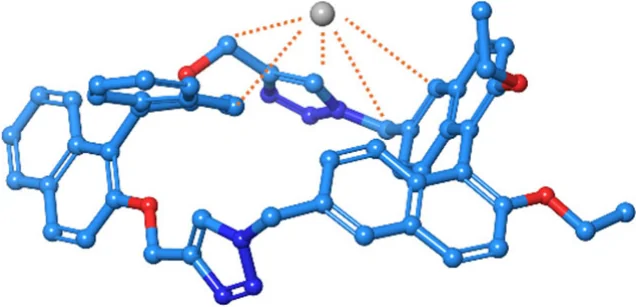
3D model of the interacting moiety of **5** with the Ag^+^ cation from NMR and MD studies.

Binding constants for compounds **5** and **10** were determined via NMR titrations in the presence of Ag^+^, yielding association constants (*K*_a_) of 3.14 × 10^3^ and 1.32 × 10^2^ M^–1^, respectively. The significantly higher binding constant of macrocycle **5** indicates a superior chelation capability and suggests a more stable interaction between the macrocycle and the cation compared to that of compound **10**.

## Conclusion

In this study, we report the synthesis of two BINOL-based macrocycles (**5** and **10**) and evaluate their ability to work as metal cation chemosensors. These macrocycles are designed and synthesized starting from two enantiomeric pure BINOL units and include two triazole groups. In the characterization of the two compounds, we observe that the two macrocycles, despite being isomers, exhibit distinct UV/vis spectra with different absorption maxima, resulting in specific fluorescence profiles. Each macrocycle has demonstrated the ability to complex specific cations, exhibiting distinct fluorescence profiles. Remarkable results were observed with the Ag^+^ cation, which reported the strongest quenching effect on both macrocycles (75.2 and 60.5%, respectively), while Co^2+^ caused a significant increase in fluorescence for both compounds, especially for macrocycle **5** (73.5%). Based on these features, we conducted NMR studies of chemical shift perturbation to examine how the two cations affect each atom within the structures. The analysis revealed that Co^2+^ exerts a non-specific influence on both molecules, primarily causing signal shifts due to paramagnetic effects. In contrast, Ag^+^ binds to particular atoms in the two macrocycles, showing a higher number of residues involved in the interaction and a significantly stronger binding affinity in macrocycle **5** (*K*_a_) of 3.14 × 10^3^ M^–1^. 2D ^1^H–^1^H NOESY spectra and MD simulations were used to determine the preferred conformational states and to build a binding model of macrocycle **5** with the Ag^+^ cation. In particular, this analysis unveiled that macrocycle **5** benefits from a more closed and preorganized conformation, which promotes stronger metal/ligand interactions, enhanced binding affinity, and greater complex stability compared to the open conformation of macrocycle **10**. In Table S6, we compared macrocycles **5** and **10** to previously reported BINOL/triazole systems although non-macrocyclic (Table S6 of the Supporting Information). Macrocycle **5** represents a promising scaffold for developing multifunctional fluorescent chemosensors capable of distinguishing among metal ions via distinct signal transduction mechanisms. In conclusion, the macrocycles studied exhibit different conformational preferences, and the distinct UV/vis profiles may arise from these singular (supra)­molecular properties. Macrocycle **5** displayed a specific interaction with Ag^+^, as demonstrated by fluorescence studies, NMR titrations, binding constant determination, and MD simulations. Although Co^2+^ induced significant fluorescence enhancement, NMR data suggest that its effect may be influenced by paramagnetic perturbations; therefore, the molecular basis of this response remains to be fully elucidated. Nevertheless, the distinct fluorescence behaviors observed for Ag^+^ and Co^2+^ support macrocycle **5** as a differential metal-ion-sensing platform and potentially a “dual metal cation chemosensor”.^26^ Macrocycle **5** can be considered for further biological investigations after chemical modification to perform studies in aqueous media.

## Supplementary Material



## Data Availability

The data underlying this study are available in the published article and its Supporting Information.

## Notes

Detailed synthetic procedure and full characterization of all the synthesized compounds, with additional spectroscopic information reported concerning NMR titrations, LOD, LOQ, and binding constant determination, and competitive binding (PDF)
