# Supplementary material for: 1,1′-Bi-2,2′-naphthol-Based Macrocycles as Dual Metal Cation Chemosensors: Synthesis, Spectroscopic Characterization, and Molecular Dynamics Insights
Source: Org Lett. 2026 Aug 5;28(32):10169–74. doi: 10.1021/acs.orglett.6c02303 (PMC13488491; doi:10.1021/acs.orglett.6c02303)
Supplement: Supplementary file 1 [file ol6c02303_si_001.pdf]

Supporting Information for:

BINOL-based Macrocycles as Dual Metal Cations Chemosensors: Synthesis, Spectroscopic Characterization and Molecular Dynamics Insights

Michele Larocca,[a] Michela Buonocore,[b] Davide Alfredo Meo,[a] Manuela Grimaldi,[c] Anna Maria D'Ursi[c] and Annunziata Soriente*[a]

a *Department of Chemistry and Biology, University of Salerno, Via Giovanni Paolo II, 132 - 84084 Fisciano (SA), Italy*
b *Department of Chemical Sciences, University of Naples "Federico II", Strada Comunale Cintia, 80126 Naples, Italy*
c *Department of Pharmacy, University of Salerno, Via Giovanni Paolo II, 132 - 84084 Fisciano (SA), Italy*

Table of Contents

**Figure S1**	^{1}H-NMR spectrum of compound 1
**FigureS2**	^{13}C-NMR spectrum of compound 1
**Figure S3**	^{1}H-NMR spectrum of compound 2
**Figure S4**	^{13}C-NMR spectrum of compound 2
**Figure S5**	^{1}H-NMR spectrum of compound 3
**Figure S6**	^{13}C-NMR spectrum of compound 3
**Figure S7**	^{1}H-NMR spectrum of compound 4
**Figure S8**	^{13}C-NMR spectrum of compound 4
**Figure S9**	^{1}H-NMR spectrum of compound 6
**Figure S10**	^{13}C-NMR spectrum of compound 6
**Figure S11**	^{1}H-NMR spectrum of compound 7
**Figure S12**	^{13}C-NMR spectrum of compound 7
**Figure S13**	^{1}H-NMR spectrum of compound 8
**Figure S14**	^{13}C-NMR spectrum of compound 8
**Figure S15**	^{1}H-NMR spectrum of compound 9
**Figure S16**	^{13}C-NMR spectrum of compound 9
**Figure S17**	1H NMR spectrum of 5 in DMSO-d6
**Figure S18**	HSQC spectrum of 5 in DMSO-d6
**Figure S19**	HMBC spectrum of 5 in DMSO-d6
**Figure S20**	COSY spectrum of 5 in DMSO-d6
**Figure S21**	NOESY spectrum of 5 in DMSO-d6
**Figure S22**	1H NMR spectrum of 5 in DMSO-d6 after addition of 1 equiv. of AgNO3
**Figure S23**	HSQC spectrum of 5 in DMSO-d6 after addition of 1 equiv. of AgNO3
**Figure S24**	1H NMR spectrum of 5 in DMSO-d6 after addition of 2 equiv. of AgNO3
**Figure S25**	HSQC spectrum of 5 in DMSO-d6 after addition of 2 equiv. of AgNO3
**Figure S26**	1H NMR spectrum of 5 in DMSO-d6 after addition of 3 equiv. of AgNO3
**Figure S27**	HSQC spectrum of 5 in DMSO-d6 after addition of 3 equiv. of AgNO3
**Figure S28**	1H NMR spectrum of 5 in DMSO-d6 after addition of 4 equiv. of AgNO3
**Figure S29**	HSQC spectrum of 5 in DMSO-d6 after addition of 4 equiv. of AgNO3
**Figure S30**	NOESY spectrum of 5 in DMSO-d6 after addition of 4 equiv. of AgNO3
**Table S1**	1H and 13C chemical shifts of 5 in DMSO-d6 in absence and in presence of 1, 2, 3, 4 equiv. of AgNO3
**Figure S31**	1H NMR spectrum of 5 in DMSO-d6 after addition of 1 equiv. of CoCl2
**Figure S32**	HSQC spectrum of 5 in DMSO-d6 after addition of 1 equiv. of CoCl2

**Figure S33**	1H NMR spectrum of 5 in DMSO-d6 after addition of 2 equiv. of CoCl2
**Figure S34**	HSQC spectrum of 5 in DMSO-d6 after addition of 2 equiv. of CoCl2
**Figure S35**	1H NMR spectrum of 5 in DMSO-d6 after addition of 3 equiv. of CoCl2
**Figure S36**	HSQC spectrum of 5 in DMSO-d6 after addition of 3 equiv. of CoCl2
**Figure S37**	1H NMR spectrum of 5 in DMSO-d6 after addition of 4 equiv. of CoCl2
**Figure S38**	HSQC spectrum of 5 in DMSO-d6 after addition of 4 equiv. of CoCl2
**Figure S39**	NOESY spectrum of 5 in DMSO-d6 after addition of 4 equiv. of CoCl2
**Table S2**	1H and 13C chemical shifts of 5 in DMSO-d6 in absence and in presence of 1, 2, 3, 4 equiv. of CoCl2
**Figure S40**	1H NMR spectrum of 10 in DMSO-d6
**Figure S41**	HSQC spectrum of 10 in DMSO-d6
**Figure S42**	HMBC spectrum of 10 in DMSO-d6
**Figure S43**	COSY spectrum of 10 in DMSO-d6
**Figure S44**	NOESY spectrum of 10 in DMSO-d6
**Figure S45**	1H NMR spectrum of 10 in DMSO-d6 after addition of 1 equiv. of AgNO3
**Figure S46**	HSQC spectrum of 10 in DMSO-d6 after addition of 1 equiv. of AgNO3
**Figure S47**	1H NMR spectrum of 10 in DMSO-d6 after addition of 2 equiv. of AgNO3
**Figure S48**	HSQC spectrum of 10 in DMSO-d6 after addition of 2 equiv. of AgNO3
**Figure S49**	1H NMR spectrum of 10 in DMSO-d6 after addition of 3 equiv. of AgNO3
**Figure S50**	HSQC spectrum of 10 in DMSO-d6 after addition of 3 equiv. of AgNO3
**Figure S51**	1H NMR spectrum of 10 in DMSO-d6 after addition of 4 equiv. of AgNO3
**Figure S52**	HSQC spectrum of 10 in DMSO-d6 after addition of 4 equiv. of AgNO3
**Figure S53**	NOESY spectrum of 10 in DMSO-d6 after addition of 4 equiv. of AgNO3
**Table S3**	1H and 13C chemical shifts of 10 in DMSO-d6 in absence and in presence of 1, 2, 3, 4 equiv. of AgNO3
**Figure S54**	1H NMR spectrum of 10 in DMSO-d6 after addition of 1 equiv. of CoCl2
**Figure S55**	HSQC spectrum of 10 in DMSO-d6 after addition of 1 equiv. of CoCl2
**Figure S56**	1H NMR spectrum of 10 in DMSO-d6 after addition of 2 equiv. of CoCl2
**Figure S57**	HSQC spectrum of 10 in DMSO-d6 after addition of 2 equiv. of CoCl2
**Figure S58**	1H NMR spectrum of 10 in DMSO-d6 after addition of 3 equiv. of CoCl2
**Figure S59**	HSQC spectrum of 10 in DMSO-d6 after addition of 3 equiv. of CoCl2
**Figure S60**	1H NMR spectrum of 10 in DMSO-d6 after addition of 4 equiv. of CoCl2
**Figure S61**	HSQC spectrum of 10 in DMSO-d6 after addition of 4 equiv. of CoCl2
**Figure S62**	NOESY spectrum of 10 in DMSO-d6 after addition of 4 equiv. of CoCl2
**Table S4**	1H and 13C chemical shifts of 10 in DMSO-d6 in absence and in presence of 1, 2, 3, 4 equiv. of CoCl2
**Figure S63**	Calibration plot of **5** (concentration range 0-14 µM) at 281 nm using UV/Vis spectroscopy
**Figure S64**	Determination of LOD and LOQ of **5** (concentration range 0-5 µM) at 281 nm using UV/Vis spectroscopy
**Figure S65**	Determination of the Ka of 5 by fluorescence spectroscopy according to the calculation method according to the literature[4] (the concentration of 5 is 11 µM in MeOH/DMSO, *v/v*, 99/1 plus Ag^+ at 0, 1, 5, 10, 20 and 30 equivalents).
**Figure S66**	Competitive binding of the macrocycle 5 in presence of Ag^+ plus Zn^{2+} and Cu^{2+} as the two other cation which provide also fluorescence quenching. Molar ratios: 5 = 11 µM and 5:Ag+:Zn2+ or 5:Ag+:Cu2+ = 1:20:20 equivalents
**Figure S67**	Quantitative metrics for MD simulations of free macrocycle 5 and macrocycle 5 in complex with Ag^+ cation
**Table S5**	Relative fluorescence quantum yield calculated for Macrocycle 5 and macrocycle 10 in the presence of the reported cations
**Figure S68**	ESI-MS spectrum of the complex between 5 and Ag^+.
**Figure S69**	ESI-MS spectrum of the complex between 5 and Co^{2+}.
**Figure S70**	CD spectra of the macrocycles **5** (solid line) and **10** (dashed line) in MeOH/DMSO, *v/v*, 99/1 (200 µM).
**Table S6**	Comparison of the sensing performance of macrocycles **5** and **10** with representative BINOL/triazole-based fluorescent chemosensors reported in the literature.

- **Synthetic general procedures**

The chemicals were purchased from various providers. Reactions were monitored for completion by analysing a small sample by TLC. Thin layer chromatography (TLC): Merck TLC plates silica gel 60 on aluminium with the indicated solvent system; the spots were visualized by UV light (254 nm and 366 nm). ^{1}H-NMR and ^{13}C-NMR spectra were performed in the indicated solvent; Bruker AV-400 (400 MHz); AV-400 (100.0 MHz); δ in ppm, J in Hz. Halogenated compounds were handled appropriately according to the safety procedures including the use of protecting agents.

- **UV/Vis and Fluorescence Studies**

The macrocycles **5** and **10** were studied by means of fluorescence spectroscopy in order to address their ability to selectively recognize metal cations of biological interest. Prior to perform Host-Guest fluorescence interaction studies, each of the above-mentioned macrocycle was analysed by means of UV/Vis spectroscopy at a concentration equal to 20 µM in MeOH/DMSO, *v/v*, 99/1. UV/Vis spectra are reported in Figure 5. Once obtained the absorption maxima (Figure 5) for each of the two macrocycles **5** (λ_{max} = 281 nm) and **10** (λ_{max} = 338 nm), fluorescence spectroscopy studies were carried out aiming at figuring out whether the macrocycles were able to selectively bind/recognize any metal cation of biological interest. The metal cation tested, for both the macrocycles, are the followings: Na^{+} (from NaCl), Li^{+}(from LiCl), K^{+}(from KF), Ag^{+}(from $AgNO_3$), Mg^{2+}(from $MgCl_2\cdot6H_2O$), Ca^{2+}(from $CaCl_2$), Cu^{2+}(from $CuSO_4\cdot5H_2O$), Zn^{2+}(from $Zn(CH_3COO^{-})_2\cdot2H_2O$), Ni^{2+}(from $NiCl_2$) and Co^{2+}(from $CoCl_2\cdot6H_2O$). Each macrocycle was tested at a final concentration of 11uM with the cation added to reach a final molar ratio equal to 20 equivalents with respect to the macrocycles (solution prepared in MeOH/DMSO, *v/v*, 99/1, with prior dissolution of the macrocycles in pure DMSO and the cations in pure MeOH). The fluorescence results are reported in Figure 5.

- **CD spectroscopy**

For CD analysis in $CHCl_3$, the compounds **5** and **10** were brought to a final concentration of 0.15 mM. The CD spectra were obtained using a JASCO J-810 spectropolarimeter, with the aid of a 1 mm long quartz cell and working at a temperature of 25 °C. The CD spectra were obtained by an average of 4 scans, in a measuring range 260–190 nm, at a bandwidth of 1 nm and, at a scanning speed of 10 nm/min. Each spectrum was processed by subtracting the solvent spectrum. The processing of the CD curves was performed using the software JASCO Spectra Analysis.

- **NMR Analysis**

Two different solutions of the macrocycles **5** and **10** (0.5 mg/mL in DMSO-d6 solution) were prepared and ^{1}H NMR spectra and 2D homonuclear and heteronuclear experiments were recorded at 298 K using a Bruker Avance II 500 spectrometer equipped with a BBI with z-gradient probe and processed using TOPSPIN 3.2 software package. The two solutions of **5** and **10** (0.5 mg/mL) were used to perform two titrations with two salts for both isomers. The two salts used were silver chloride and cobalt chloride and various amounts of the salts were added (1:1 eq., 1:2 eq., 1:3 eq., 1:4 eq., **5** or **10**: salts). For each isomer and for each titration point we have acquired one- and two-dimensional experiments (details are reported in Supporting Information). All homonuclear spectra were collected using a spectral width of 12 ppm, a time domain size of 4096 on F2 and of 256 on F1, 32 scans, 16 dummy scan, a relaxation delay of 1.0 s and an acquisition time of 0.24 s. The mixing time of the NOESY experiments used for interproton distance determination was 800 ms. Peak volumes obtained by NOESY and ^{1}H–^{13}C-HSQC spectra were translated into upper distance bounds using the *noeassign* macro in CYANA 3.1 software package. [18] The generated list containing the constraints was used to generate the structures, following the standard CYANA protocol. A custom molecular topology and parameter library specific to the macrocycle was generated using Cylib, ensuring an accurate representation of the molecule's covalent structure and stereochemistry. A total of 100 conformers were calculated, and the 20 lowest-energy structures with the best agreement to the experimental restraints were selected to represent the final ensemble. Structural quality was assessed based on CYANA target function values and restraint violation statistics.

The structures obtained were visualized with Maestro by Schrödinger [19] and used for MD calculations.

Heteronuclear bidimensional spectra were collected by using a pulse sequence with multiplicity selection and a spectral width of 12 ppm for the ^{1}H nucleus and 150 ppm for the ^{13}C nucleus, a time domain size of 4096 on F2 and of 256 on F1, 64 scans, 16 dummy scans, a relaxation delay of 1.0 s, an acquisition time of 0.31 s, and 145 Hz as J(XH), 8 Hz as J(XH) long range.

- **Molecular Dynamics**

Classical MD simulations were performed using GROMACS 2025 on the NMR-derived structures of the macrocycles. Each system was solvated in DMSO, and both the topologies of DMSO molecules and the macrocyclic compounds were generated using CGenFF[1,2]. For all simulations, CHARMM36

force field[3], updated in July 2022, was employed; this version includes parameters for metal cations. Before production runs, all systems underwent an initial energy minimization using the steepest descent algorithm, followed by a two-phase equilibration: NVT equilibration (300 K) for 1 ns using the V-rescale thermostat, and NPT equilibration (1 bar) for 1 ns the Parrinello-Rahman barostat. Subsequently, 100 ns of production MD simulations were carried out under periodic boundary conditions. Coordinates were saved every 10 ns for analysis.

Design and synthetic procedures concerning the macrocycle **5** herein described, is schematically reported in Figure 2:

- Compound **1** ((*S*)-2,2'-Diethoxy-[1,1']binaphthalenyl): In a two-necks round bottom flask (S)-BINOL (3.0 g, 1 eq., 10.48 mmol) was dissolved in 16.7 mL of anhydrous DMF, then, upon dissolution, potassium carbonate (5.79 g, 4 eq., 41.91mmol) was added and the mixture stirred at 50°C for 30 minutes by means of a heating mantle. Subsequently, after cooling down at room temperature, ethyl iodide (9.8 g, 5.05 mL, 6 eq., 62.87 mmol) was added, and the temperature of the system was increased up to 50°C refluxing and stirred continuously for 24 hours. After cooling down at room temperature, 18 mL of deionized water was added to precipitate the product and selectively dissolve the potassium carbonate. The suspension was filtered on a Gooch frit, washed with water (3 x 5 mL) and the product collected from the frit and dried under high vacuum to afford a white solid in a yield of 90.5% (3.29 g).

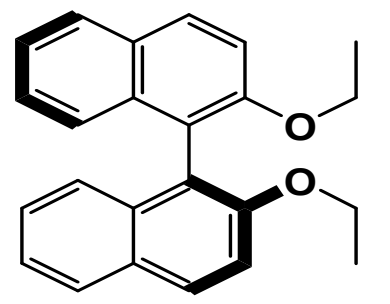

1

^{1}H-NMR: (CD_2Cl_2, 400 MHz) δ 8.02 (2H, d, *J* = 8.8 Hz), 7.92 (2H, d, *J* = 8.0 Hz), 7.49 (2H, d, *J* = 9.2 Hz), 7.36 (2H, t, *J* = 7.6 Hz), 7.25 (2H, t, *J* = 6.8 Hz), 7.15 (2H, d, *J* = 8.0 Hz), 4.11 (4H, dd, *J* = 6.8 Hz, *J* = 13.6 Hz), 1.12 (6H, t, *J* = 7.0 Hz).

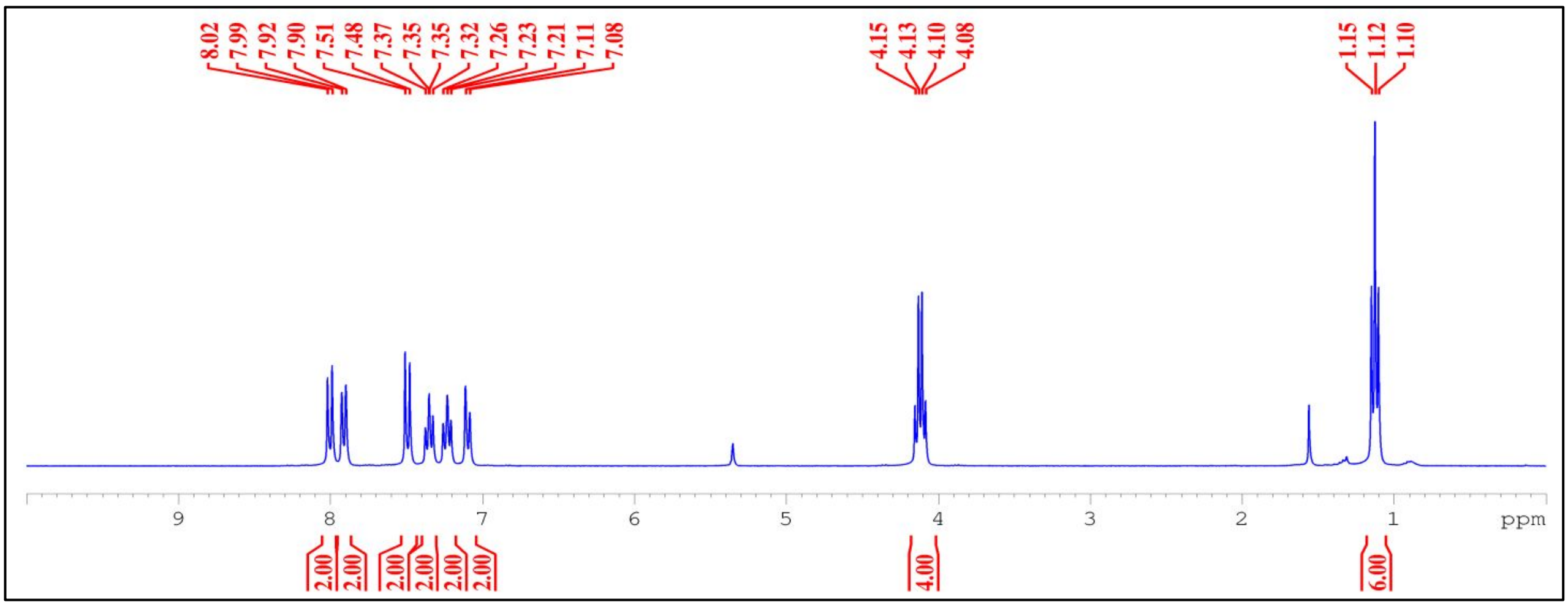

Figure S1. ^{1}H-NMR spectrum of compound 1.

^{13}C-NMR: (CD_2Cl_2, 100 MHz) 154.8, 134.6, 129.7, 129.5, 128.3, 126.5, 126.0, 123.9, 121.1, δ 116.3, 65.7, 15.5.

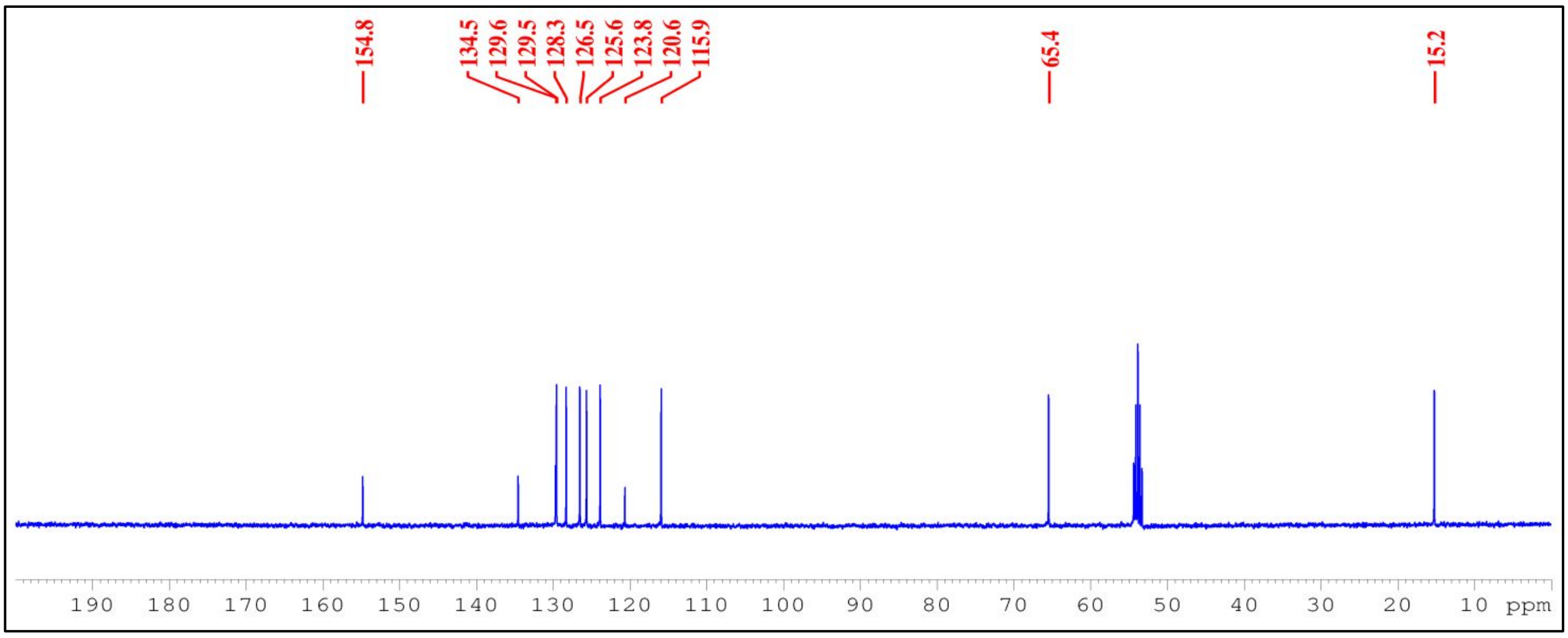

FigureS2. ^{13}C-NMR spectrum of compound 1.

(+)-MALDI-FTICR-MS ($CHCl_3$) m/z: $[M]^{·+}$ Calcd for C24H22O2 342.1614; Found 342.1618.

- Compound **2**, ((*S*)-6,6'-Bis-bromomethyl-2,2'-diethoxy-[1,1']binaphthalenyl): in a two necks round bottom flask, compound **1** (2,2'-Diethoxy-[1,1']binaphthalenyl, 1.00 g, 1 eq., 2.92mmol) and paraformaldehyde (0.51 g, 5.8 eq., 16.94 mmol) were dissolved in 24.3 mL of glacial acetic acid, then, upon dissolution under continuous stirring, hydrobromic acid (30% in acetic acid, 1.37g, 2.91 mL, 5.8 eq., 16.94 mmol) was added and the mixture stirred at r.T. for 90 minutes. Subsequently, the reaction mixture was protected from light, warmed up at 70°C using a heating mantle and left overnight (about 16 hs) under continuous stirring and refluxing. The mixture was cooled down at r.T. and 50 mL of deionized water were added to obtain a white precipitate that was filtrated, washed with water (2 x 50mL), collected and dried. The crude obtained (1.50 g) was further handled, after being split up in three aliquots of 0.50 g, to isolate the product with the following procedure: for each aliquot of 0.50 g, 300 µL of DCM each 0.1 g of crude solid were added and warmed up to 50°C to entirely solubilize the crude. Upon dissolution, using a dropping funnel, 40 mL of hexane were added dropwise while cooling down the system at r.T. An orange sticky solid precipitates while the product is selectively solubilised in hexane. The hexane pale white solution was transferred in a separatory funnel by filtrating on a paper filter, hence the hexane solution was washed only one time with 10 mL of water. After collecting the organic phase, it was directly dried using a rotavapor and high vacuum (without prior drying with Na_2SO_4 because the product might be partially damaged and dispersed within the drying solid agent) and a pale yellow foamy solid affords in 58.5% of yield (0,88 g).

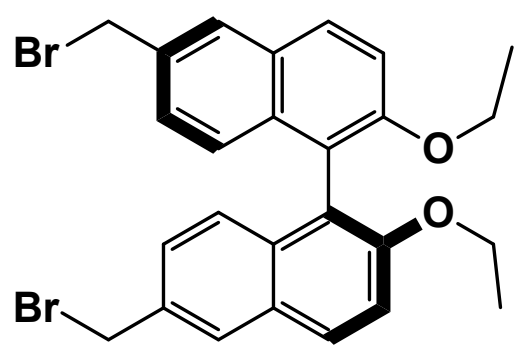

2

^{1}H-NMR: (CD_2Cl_2, 400 MHz) δ 7.99 (2H, s), 7.91(2H, s), 7.50 (2H, d, *J* = 9.0 Hz), 7.26 (2H, d, *J* = 8.7 Hz), 7.08 (2H, d, *J* = 8.7 Hz), 4.69 (4H, s), 4.12 (4H, dd, *J* = 6.9, 6.8 Hz), 1.13 (6H, t, *J* = 6.8 Hz,).

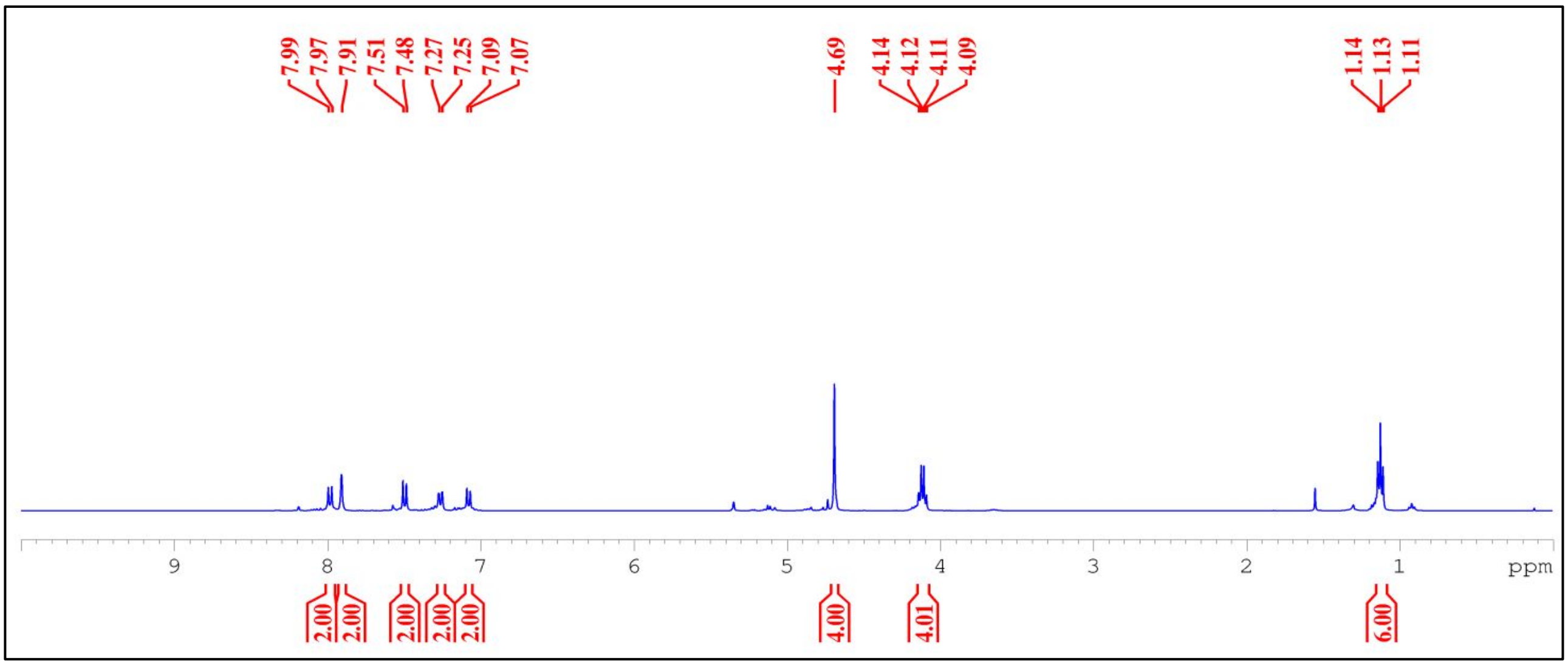

Figure S3. ^{1}H-NMR spectrum of compound 2.

^{13}C-NMR: (CD_2Cl_2, 100 MHz) δ 155.0, 133.7, 132.8, 129.4, 128.7, 128.0, 127.1, 126.0, 115.7, 64.9, 34.5, 14.7.

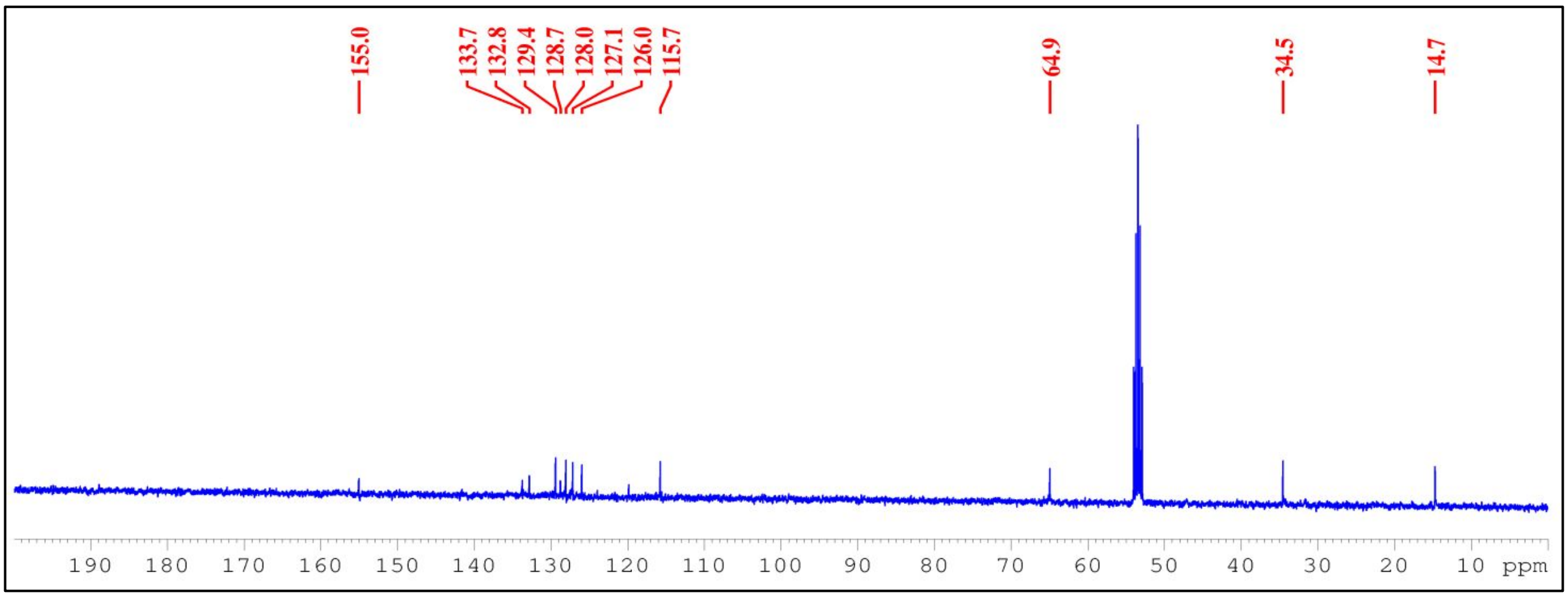

Figure S4. ^{13}C-NMR spectrum of compound 2.

(+)-MALDI-FTICR-MS ($CHCl_3$) m/z: $[M + H]^+$ Calcd for C26H24Br2O2H 529.0122; Found 529.0170.

- Compound **3** ((*S*)-6,6'-Bis-azidomethyl-2,2'-diethoxy-[1,1']binaphthalenyl): in a two necks round bottom flask, compound **2** ((*S*)-6,6'-Bis-bromomethyl-2,2'-diethoxy-[1,1']binaphthalenyl, 306 mg, 1 eq., 0.58 mmol) was solubilised in dry DMSO (3.3 mL) prior to add Sodium Azide (226 mg, 6 eq., 3.48 mmol). The reaction solution was warmed up to 28°C with a heating mantle and left overnight (about 15 hs) under continuous stirring. Subsequently, 15 mL of deionized H_2O were added to quench the reaction, then, the mixture was transferred in a separatory funnel and extracted with DCM (3 x 15 mL). The organic phases were collected, dried over Na_2SO_4 and concentrated by means of a rotavapor and high vacuum. The product was purified by means of semipreparative TLC after having split the crude solid in two aliquots: each aliquot was solubilized in a minimal amount of DCM and distributed on a silica glass slide of 2 mm thickness. After deposition, two elutions were carried out using each time 200 mL of mobile phase made of DCM/Hexane: *v/v*, 8/2; the product of interest has exhibited an R_F = 0.75 after the second run and was recovered by pouring the scratched silica into a chromatography column to elute the desired product by adding 100 mL of DCM followed by 50 mL of Ethyl Acetate. The combined solutions were concentrated and the product afforded as a white solid equal to 145 mg (Y% = 55%).

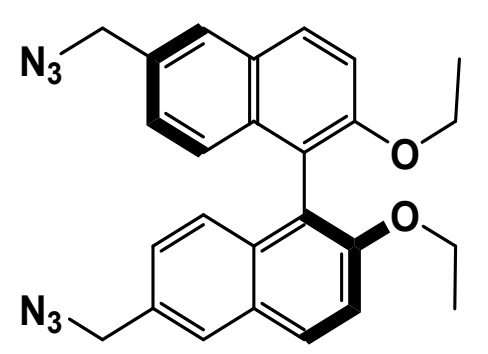

3

^{1}H-NMR: (CD_2Cl_2, 400 MHz) δ 8.02 (2H, d, *J* = 8.8 Hz), 7.84 (2H, s), 7.50 (2H, d, *J* = 9.1 Hz), 7.19 (2H, d, *J* = 8.8 Hz), 7.11 (2H, d, *J* = 8.4 Hz), 4.48(4H, s), 4.11 (4H, dd, *J* = 6.8 Hz), 1.11 (6H, t, *J* = 6.8 Hz).

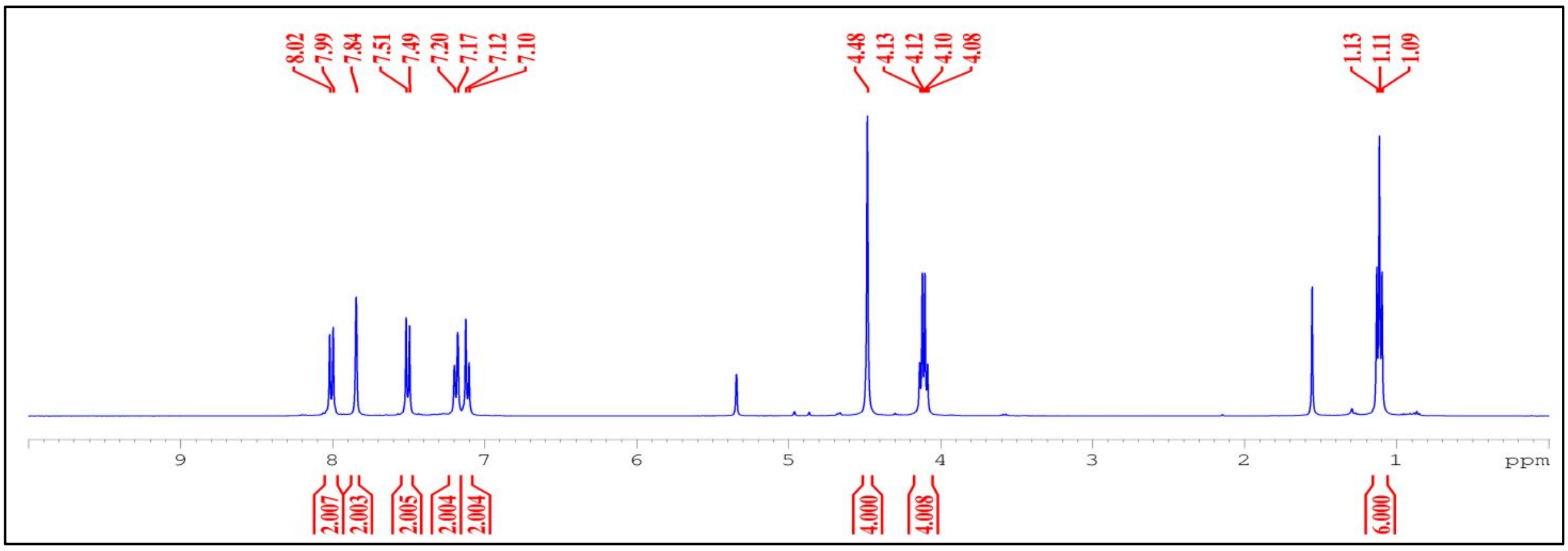

Figure S5. ^{1}H-NMR spectrum of compound 3.

^{13}C-NMR: (CD_2Cl_2, 100 MHz) δ 154.8, 133.7, 130.6, 129.3, 128.8, 127.3, 126.3, 125.9, 119.9, 115.8, 65.0, 54.9, 14.7.

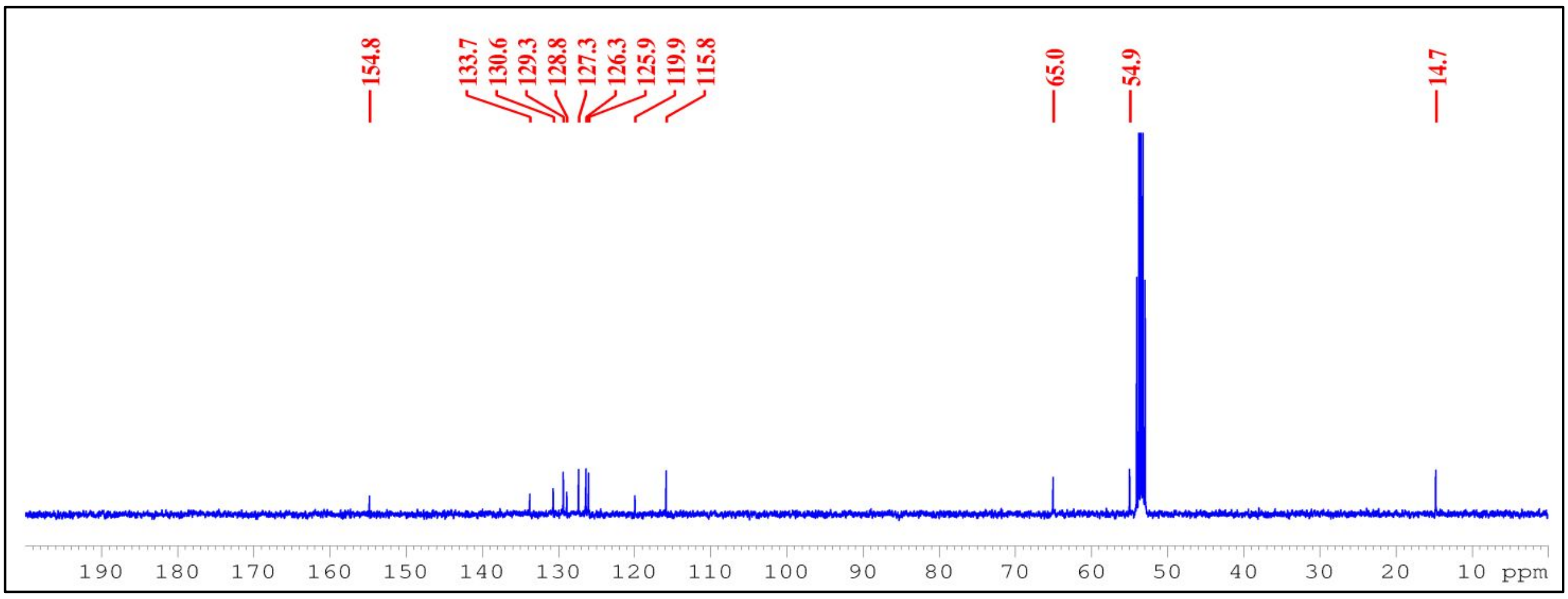

Figure S6. ^{13}C-NMR spectrum of compound 3.

(+)-MALDI-FTICR-MS ($CHCl_3$) m/z: [M]$^{·+}$ Calcd. for C26H24N6O2 452.1955; Found 452.1956

- Compound **4** ((*S*)-2,2'-Bis-prop-2-ynyloxy-[1,1']binaphthalenyl): (S)-BINOL (500 mg, 1 eq., 1.75 mmol) was dissolved in 3.1 mL of anhydrous DMF, then potassium carbonate (965 mg, 4 eq., 6.99 mmol) was added and the mixture stirred at room temperature for 30 minutes. Subsequently, propargylbromide (1.247 g, 0.993 mL, 6 eq., 10.48 mmol) was added and the temperature of the system was increased up to 50°C with a heating mantle refluxing for 5 hours. After cooling down at r.T., 6 mL of deionized H_2O was added and the mixture stirred for 5 minutes to selectively dissolve the potassium carbonate. The mixture was transferred in a separatory funnel to extract with DCM (3 x 6 mL), thus, the organic phases were collected, dried over sodium sulphate, filtered and concentrated to remove the excess of DCM. A yellow solution affords; thus, the product was precipitated by adding 6 mL of deionized water and filtered on a Gooch frit. The solid in the frit was washed with abundant water (45 mL) and dried at the high vacuum to afford 520 mg (Y% = 83%) as a pale-yellow solid.

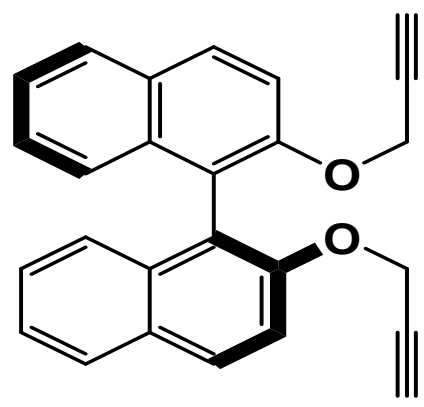

4

^{1}H-NMR: ($CDCl_3$, 400 MHz) δ 8.05 (2H, d, *J* = 9.2Hz), 7.94 (2H, d, *J* = 8.4 Hz), 7.62 (2H, d, *J* = 9.2 Hz), 7.40 (2H, t, *J* = 7.2Hz), 7.26 (2H, t, *J* = 7.6 Hz), 7.12 (2H, d, *J* = 8.4 Hz), 4.71 (4H, s), 2.53 (2H, s).

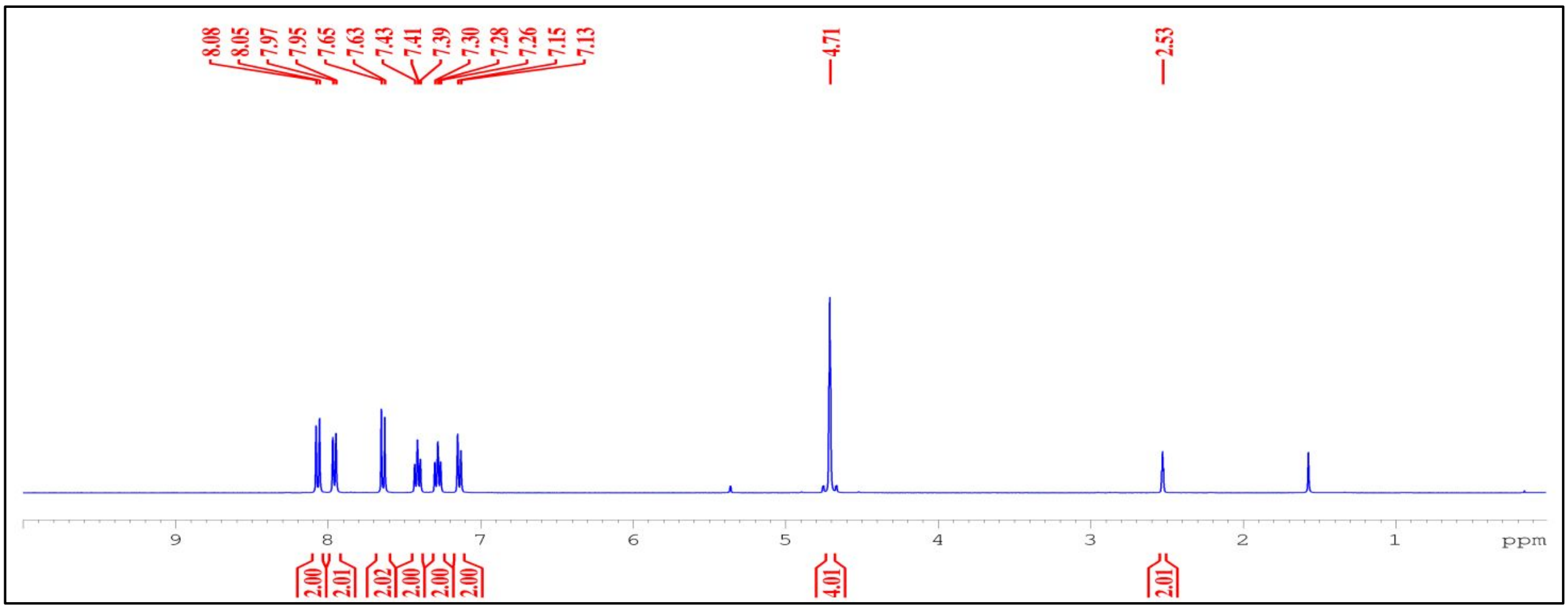

Figure S7. ^{1}H-NMR spectrum of compound 4.

^{13}C-NMR: ($CDCl_3$, 100 MHz) δ 153.1, 133.9, 129.7, 129.4, 128.0, 126.5, 125.3, 124.1,120.3, 115.5, 79.1, 75.1, 57.1.

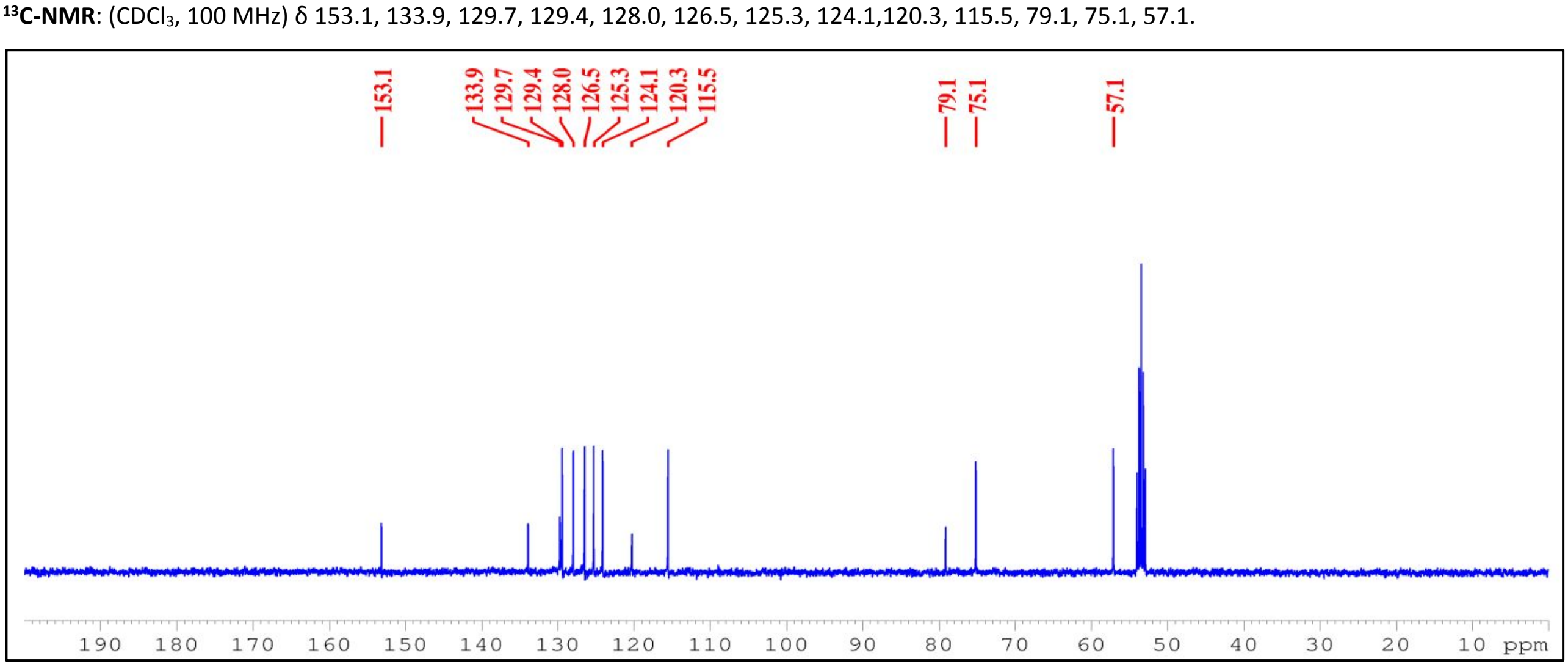

Figure S8. ^{13}C-NMR spectrum of compound 4.

(+)-MALDI-FTICR-MS ($CHCl_3$) m/z: $[M]^{\cdot+}$ Calcd for C26H18O2 362.1301; Found 362.1314.

- Compound **5** ($(4^4Z,11^4Z)$-$1^2,2^2$-diethoxy-$4^1H,11^1H$-6,9-dioxa-4(1,4),11(4,1)-ditriazola-1,2(1,6),7(2,1),8(1,2)-tetranaphthalenacyclododecaphane): $CuSO_4$ (63.5 mg, 2 eq., 0.397 mmol) and Sodium-L-Ascorbate (78.85 mg, 2 eq., 0.397 mmol) were dissolved at r.T. in 2 mL of a solution of H_2O/DMF, *v/v* 1/3, then compound **4** (72 mg, 1 eq., 0.199 mmol) was dissolved in 1.5 mL of DMF, added to the copper-ascorbate solution and stirred for 15 minutes at room temperature. Subsequently, compound **3** (90 mg, 1 eq., 0.199 mmol) was dissolved to 1.5 mL of DMF and added to the previous solution within compound **4**. Then the temperature increased at 60°C with a heating mantle and the reaction mixture stirred continuously for 24 hours. After cooling down the system, 20 mL of deionized H_2O were added to obtain a precipitate that was filtrated on a Gooch and washed with 40 mL of deionized H_2O. The solid was then recovered with 25 mL of $CHCl_3$, transferred into a separatory funnel and extracted with H_2O (3 x 25 mL). The organic phases were collected, dried over Na_2SO_4, filtrated and concentrated to afford 106 mg of impure yellowish product **5** that was further purified by means of a semipreparative TLC with the following conditions: TLC plate of 2 mm thickness submitted to 4 elution using 2 x 200 mL of $CHCl_3$/MeOH *v/v* 99.5/0.5 plus 2 x 200 mL of $CHCl_3$/MeOH *v/v* 99.0/1.0. The macrocycle **5** had an R_F equal to 0.26 and was obtained as a white solid in 16.6% of Yield (27 mg).

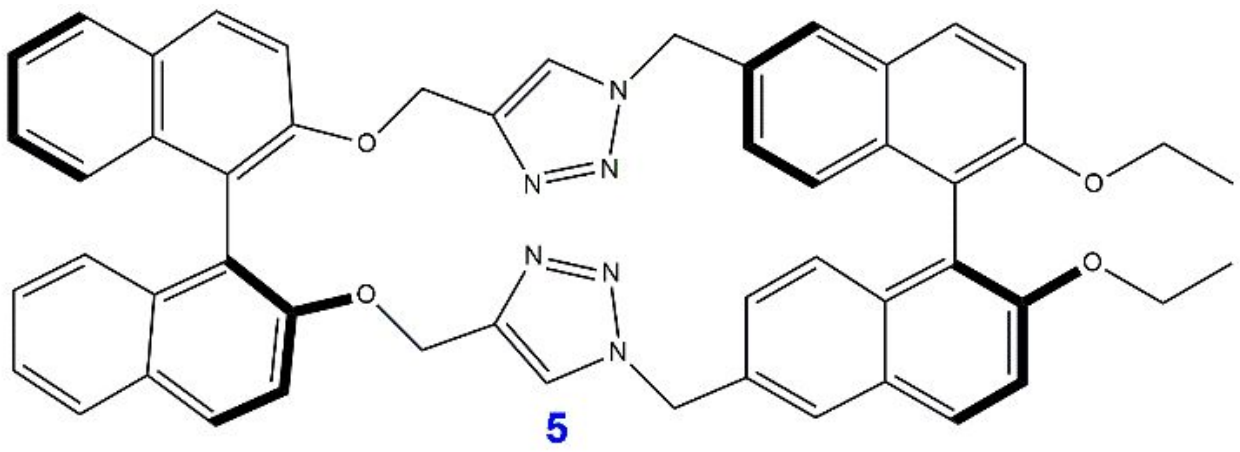

5

^{1}H-NMR: (500 MHz, DMSO-d_6) δ 7.85 (s, 1H), 7.76 (d, *J* = 9.9 Hz, 1H), 7.67 – 7.62 (m, 2H), 7.55 – 7.43 (m, 2H), 7.37 (s, 1H), 7.03 (s, 1H), 6.90 (s, 1H), 6.84 – 6.74 (m, 2H), 5.44 (d, *J* = 14.5 Hz, 2H), 5.02 – 4.93 (m, 2H), 3.96 (s, 1H), 0.91 – 0.82 (m, 3H).

^{13}C-NMR: δ 115.4, 123.0, 123.0, 127.1, 128.8, 125.4, 123.9, 63.7, 62.1, 52.3, 13.9.

(+)-MALDI-FTICR-MS ($CHCl_3$) m/z: $[M + H]^+$ Calcd for C52H42N6O4H 815.3340; Found 815.3347

Design and synthetic procedures concerning the macrocycle **10** herein described, is schematically reported in Figure 3:

- Compound **6** ((*R*)-2,2'-Diethoxy-[1,1']binaphthalenyl): In a two-necks round bottom flask (*R*)-BINOL (3.0 g, 1 eq., 10.48 mmol) was dissolved in 16.7 mL of anhydrous DMF, then, upon dissolution, potassium carbonate (5.79 g, 4 eq., 41.91mmol) was added and the mixture stirred at 50°C by means of a heating mantle for 30 minutes. Subsequently, after cooling down at room temperature, ethyl iodide (9.8 g, 5.05 mL, 6 eq., 62.87 mmol) was added and the temperature of the system was increased up to 50°C refluxing and stirred continuously for 24 hours. After cooling down at room temperature, 18 mL of deionized water was added to precipitate the product and selectively dissolve the potassium carbonate. The suspension was filtered on a Gooch frit, washed with water (3 x 5 mL) and the product collected from the frit and dried under high vacuum. The product was further purified by means of column chromatography using a silica/crude product ratio equal to 1:50 and as mobile phase DCM/Hexane, *v/v* = 1:1. A white solid was obtained in a yield of 72.5% (2.61 g).

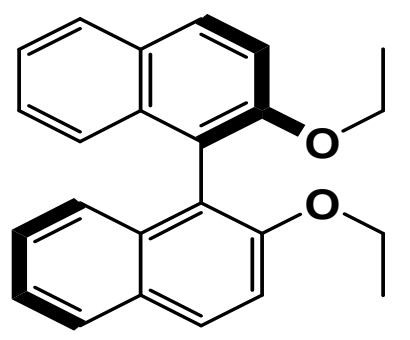

6

^{1}H-NMR: (CD_2Cl_2, 400 MHz) δ 8.02 (2H, d, *J* = 8.8 Hz), 7.94 (2H, d, *J* = 8.0 Hz), 7.52 (2H, d, *J* = 9.2 Hz), 7.37 (2H, t, *J* = 7.6 Hz), 7.25 (2H, t, *J* = 6.8 Hz), 7.15 (2H, d, *J* = 8.0 Hz), 4.11 (4H, dd, *J* = 6.8 Hz, *J* = 13.6 Hz), 1.15 (6H, t, *J* = 7.0 Hz).

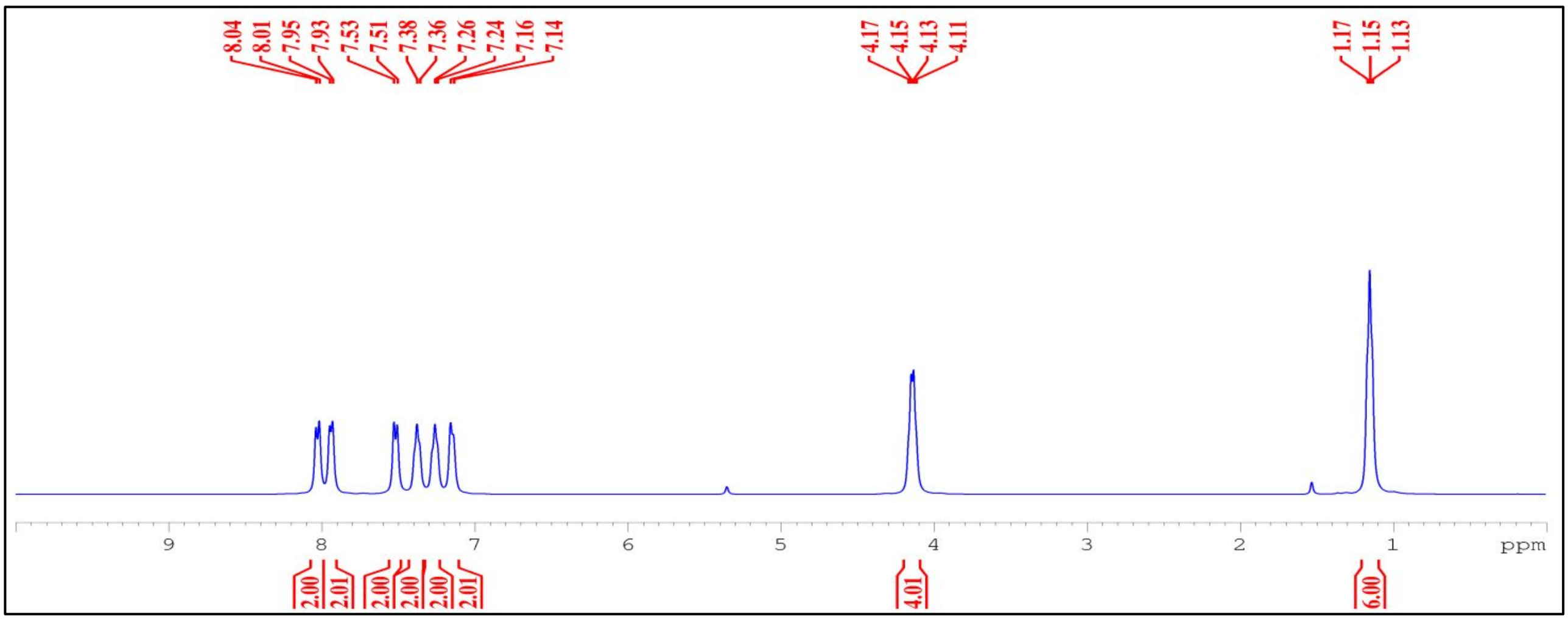

Figure S9. ^{1}H-NMR spectrum of compound 6.

^{13}C-NMR: (CD_2Cl_2, 100 MHz) δ 154.8, 134.6, 129.7, 129.6, 128.3, 126.5, 125.6, 123.9, 120.6, 115.9, 65.4, 15.2.

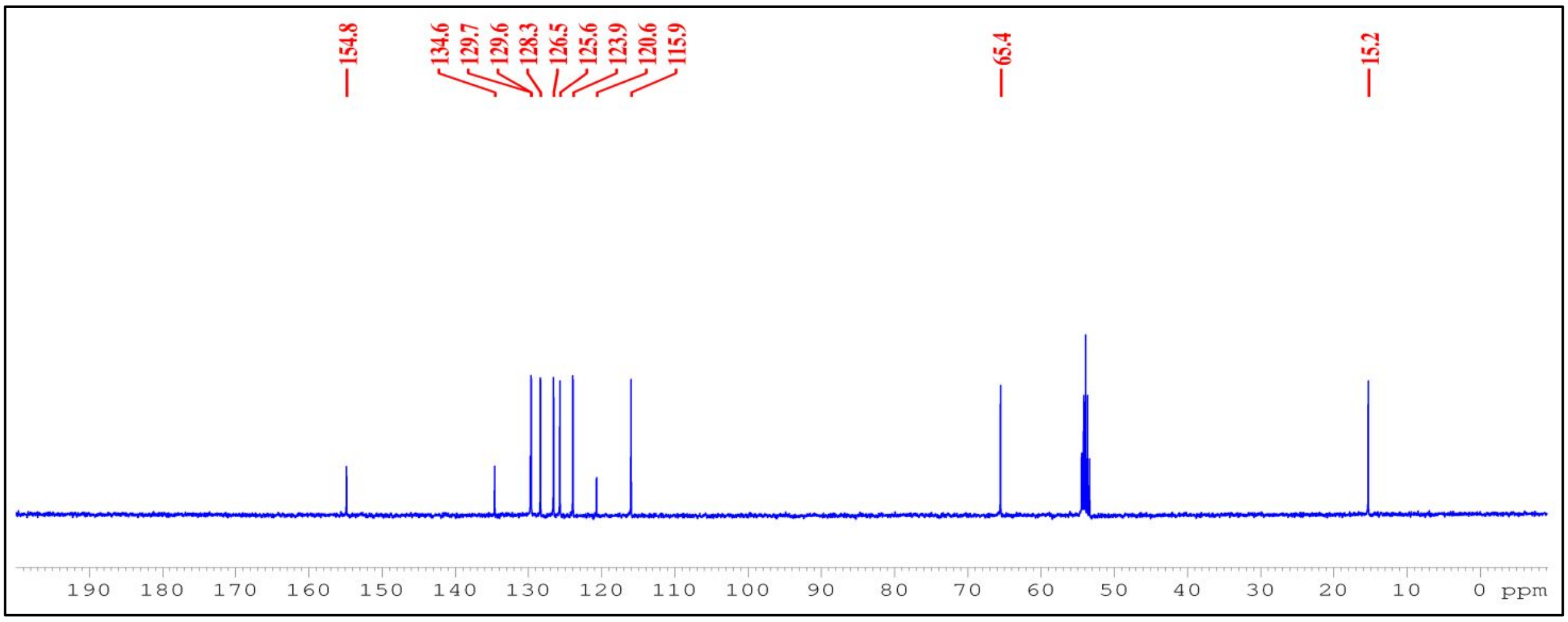

Figure S10. ^{13}C-NMR spectrum of compound 6.

(+)-MALDI-FTICR-MS ($CHCl_3$) m/z: $[M]^{\cdot+}$ Calcd for C24H22O2 342.1618; Found 342.1614.

- Compound **7**, ((*R*)-6,6'-Bis-bromomethyl-2,2'-diethoxy-[1,1']binaphthalenyl): in a two necks round bottom flask, compound **6** ((*R*)-2,2'-Diethoxy-[1,1']binaphthalenyl, 1.00 g, 1 eq., 2.92mmol) and paraformaldehyde (0.51 g, 5.8 eq., 16.94 mmol) were dissolved in 24.3 mL of glacial acetic acid, then, upon dissolution under continuous stirring, hydrobromic acid (30% in acetic acid, 1.37g, 2.91 mL, 5.8 eq., 16.94 mmol) was added and the mixture stirred at r.T. for 90 minutes. Subsequently, the reaction mixture was protected from light, warmed up at 70°C with a heating mantle and left overnight (about 16 hs) under continuous stirring and refluxing. The mixture was cooled down at r.T. and 50 mL of deionized water were added to obtain a white precipitate that was filtrated, washed with water (2 x 50mL), collected and dried. The crude obtained (1.60 g) was further handled, after being split up in three aliquots of 0.53 g, to isolate the product with the following procedure: for each aliquot of 0.53 g, 300 µL of DCM each 0.1 g of crude solid were added and warmed up to 50°C to entirely solubilize the crude. Upon dissolution, using a dropping funnel, 40 mL of hexane were added dropwise while cooling down the system at r.T. An orange sticky solid precipitates while the product is selectively solubilised in hexane. The hexane pale white solution was transferred in a separatory funnel by filtrating on a paper filter; hence the hexane solution was washed only one time with 10 mL of water. After collecting the organic phase, it was directly dried using rotavapor and high vacuum (without prior drying with Na_2SO_4 because the product might be partially damaged and dispersed within the drying solid agent) and a pale yellow foamy solid affords. The product related to each of the aliquots were collected to afford a total yield of in 34.0% of a pale yellow foamy solid (0.521 g).

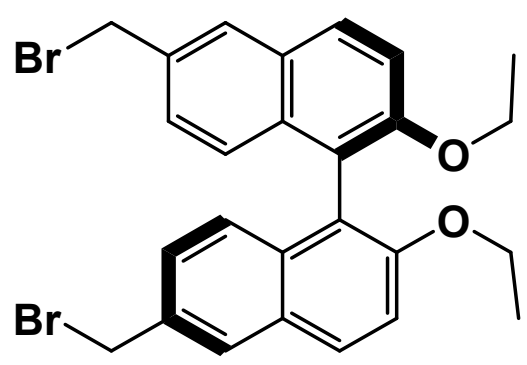

7

^{1}H-NMR: (CD_2Cl_2, 400 MHz) δ 7.98 (2H, d, *J* = 9.0 Hz), 7.91(2H, s), 7.50 (2H, d, *J* = 9.0 Hz), 7.27 (2H, d, *J* = 8.7 Hz), 7.09 (2H, d, *J* = 8.7 Hz), 4.70 (4H, s), 4.12 (4H, dd, *J* = 6.9, 6.8 Hz), 1.13 (6H, *J* = 6.8 Hz, t).

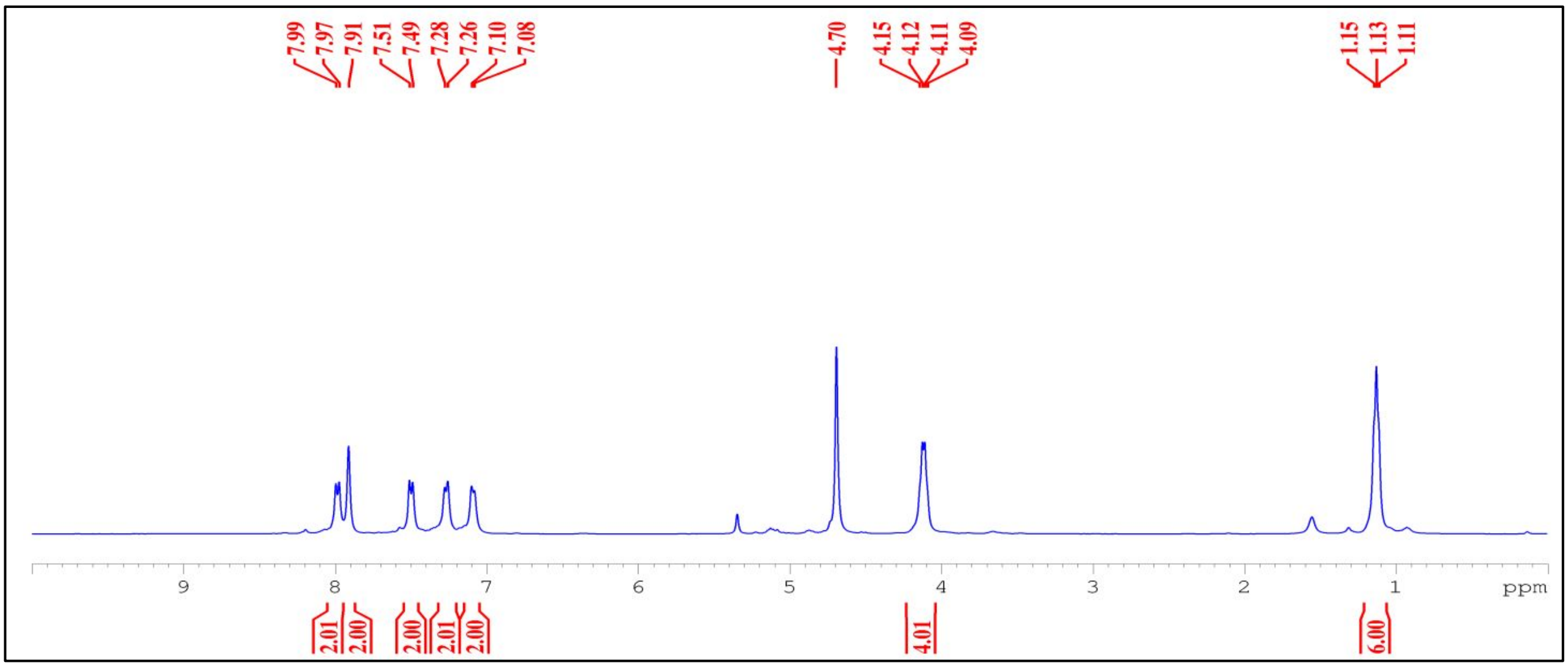

Figure S11. ^{1}H-NMR spectrum of compound 7.

^{13}C-NMR: (CD_2Cl_2, 100 MHz) δ 155.0, 133.7, 132.8, 129.4, 128.7, 128.0, 127.1, 126.0, 115.7, 64.9, 34.5, 14.7.

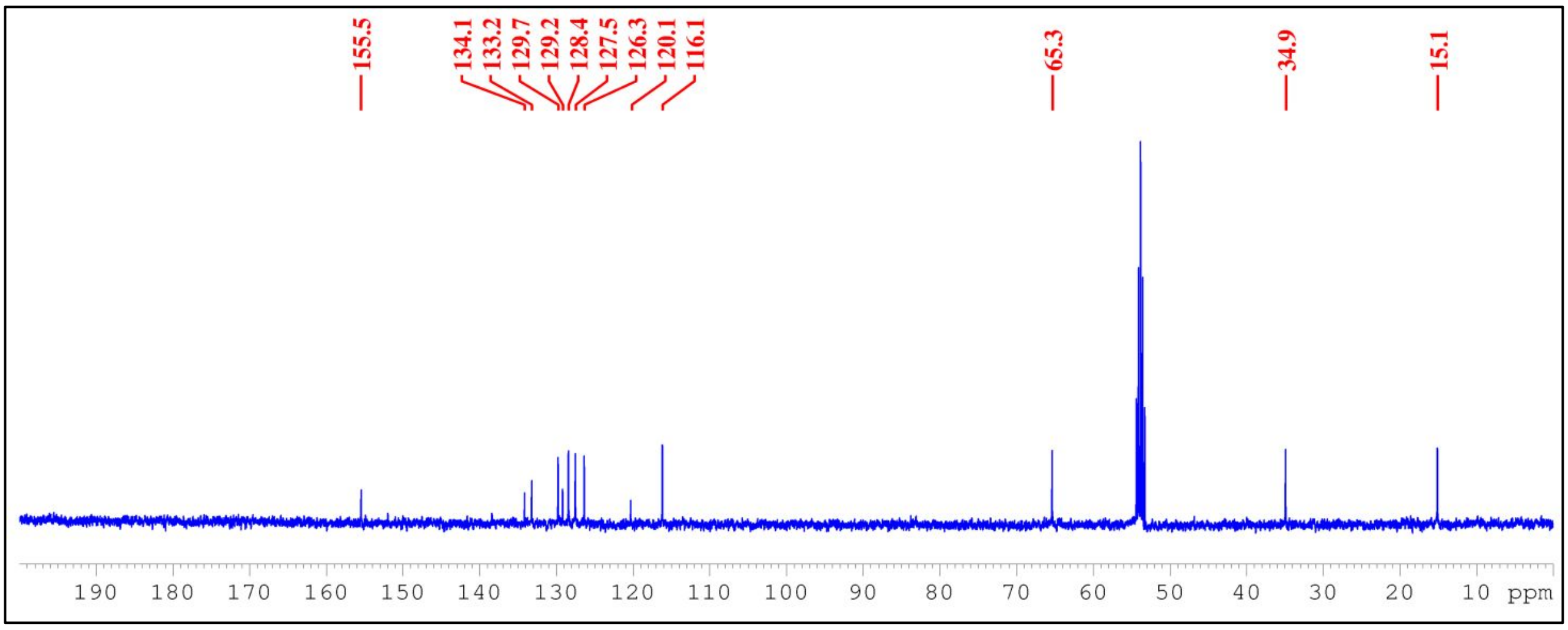

Figure S12. ^{13}C-NMR spectrum of compound 7.

(+)-MALDI-FTICR-MS ($CHCl_3$) m/z: $[M + H]^+$ Calcd for C26H24Br2O2H 529.0122; Found 529.0170.

- Compound **8** ((*R*)-6,6'-Bis-azidomethyl-2,2'-diethoxy-[1,1']binaphthalenyl): in a two necks round bottom flask, compound **7** ((*R*)-6,6'-Bis-bromomethyl-2,2'-diethoxy-[1,1']binaphthalenyl, 306 mg, 1 eq., 0.58 mmol) was solubilised in dry DMSO (3.3 mL) prior to add Sodium Azide (226 mg, 6 eq., 3.48 mmol). The reaction solution was warmed up to 28°C with a heating mantle and left overnight (about 15 hs) under continuous stirring. Subsequently, 15 mL of deionized H_2O were added to quench the reaction, then, the mixture was transferred in a separatory funnel and extracted with DCM (3 x 15 mL). The organic phases were collected, dried over Na_2SO_4 and concentrated by means of a rotavapor and high vacuum. The product was purified by means of semipreparative TLC after having split the crude solid in two aliquots: each aliquot was solubilized in a minimal amount of DCM and distributed on a silica glass slide of 2 mm thickness. After deposition, two elutions were carried out using each time 200 mL of mobile phase made of DCM/Hexane: *v/v*, 8/2; the product of interest has exhibited an R_F = 0.75 after the second run and was recovered by pouring the scratched silica into a chromatography column to elute the desired product by adding 100 mL of DCM followed by 50 mL of Ethyl Acetate. The combined solutions were concentrated and the product afforded as a white solid equal to 114.6 mg (Y% = 42.0%).

FIGURE

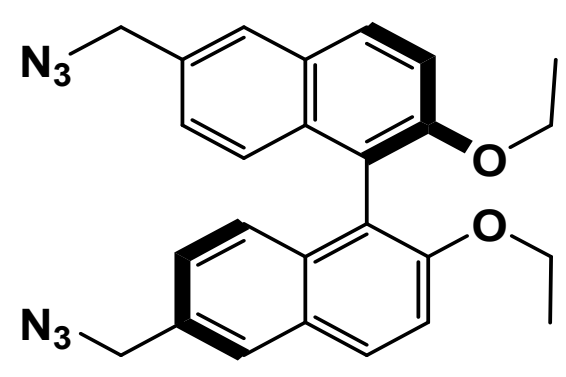

8

^{1}H-NMR: (CD_2Cl_2, 400 MHz) δ 8.02 (2H, d, *J* = 8.8 Hz), 7.85 (2H, s), 7.52 (2H, d, *J* = 9.1 Hz), 7.20 (2H, d, *J* = 8.8 Hz), 7.13 (2H, d, *J* = 8.4 Hz), 4.49(4H, s), 4.13 (4H, dd, *J* = 6.8 Hz), 1.12 (6H, t, *J* = 6.8 Hz).

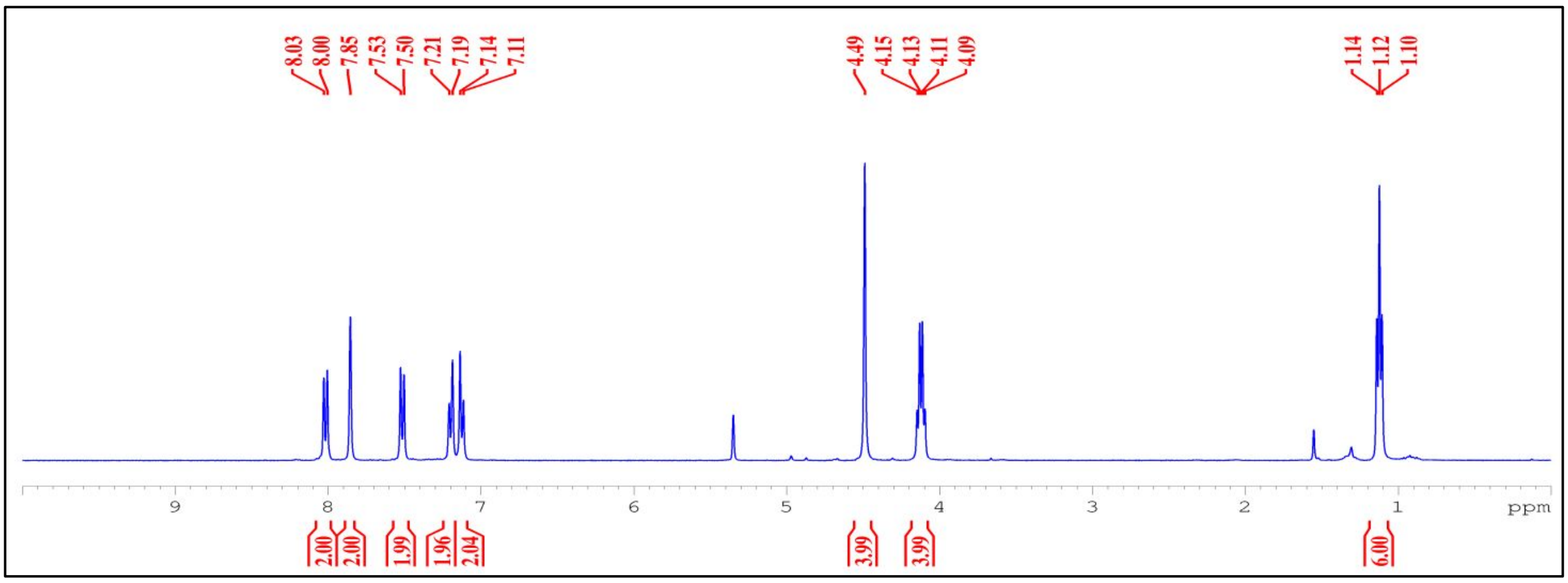

Figure S13. ^{1}H-NMR spectrum of compound 8.

^{13}C-NMR: (CD_2Cl_2, 100 MHz) δ 154.9, 133.7, 130.6, 129.3, 128.8, 127.3, 126.30, 126.0, 120.0, 115.8, 65.0, 54.9, 14.7.

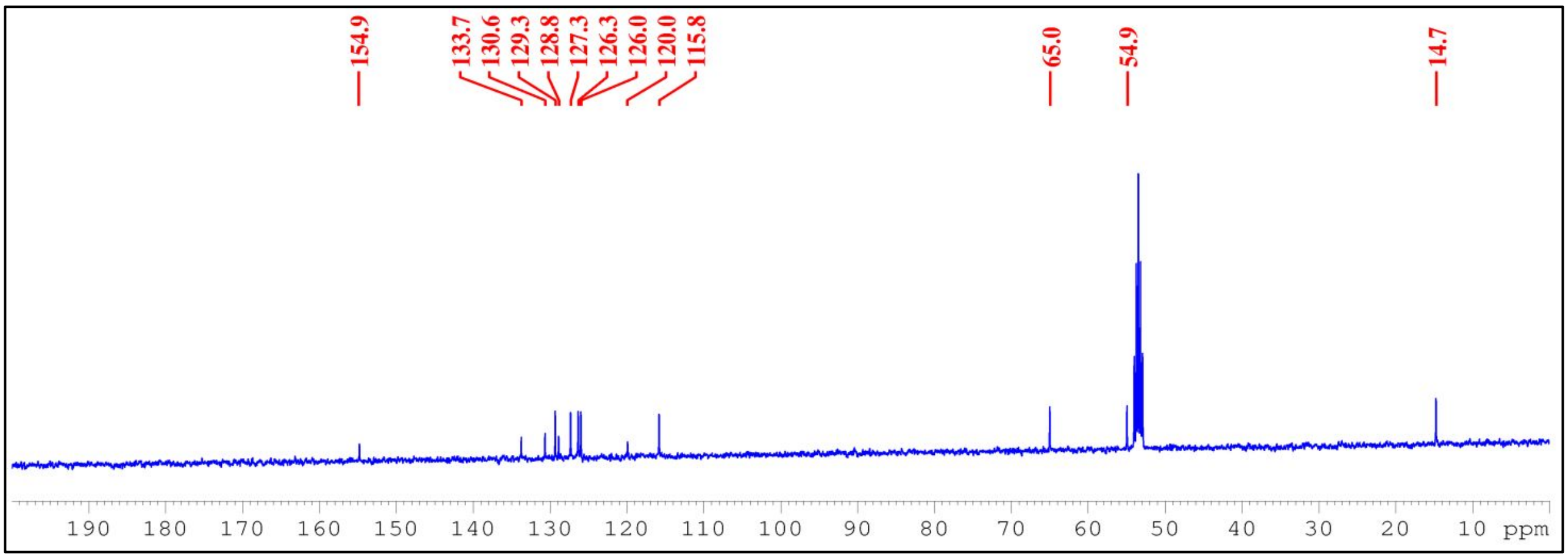

Figure S14. ^{13}C-NMR spectrum of compound 8.

(+)-MALDI-FTICR-MS ($CHCl_3$) m/z: $[M]^{\cdot+}$ Calcd. for C26H24N6O2 452.1955; Found 452.1956

- Compound **9** ((*R*)-2,2'-Bis-prop-2-ynyloxy-[1,1']binaphthalenyl): (*R*)-BINOL (500 mg, 1 eq., 1.75 mmol) was dissolved in 3.1 mL of anhydrous DMF, then potassium carbonate (965 mg, 4 eq., 6.99 mmol) was added and the mixture stirred at room temperature for 30 minutes. Subsequently, propargylbromide (1.247 g, 0.993 mL, 6 eq., 10.48 mmol) was added and the temperature of the system was increased up to 50°C with a heating mantle refluxing for 5 hours. After cooling down at r.T., 6 mL of deionized H_2O was added and the mixture stirred for 5 minutes to selectively dissolve the potassium carbonate. The mixture was transferred in a separatory funnel to extract with DCM (3 x 6 mL), thus, the organic phases were collected, dried over sodium sulphate, filtered and concentrated to remove the excess of DCM. A yellow solution affords; thus the product was precipitated by adding 6 mL of deionized water and filtered on a Gooch frit. The solid in the frit was washed with abundant water (45 mL) and dried at the high vacuum to afford 209 mg (Y% = 33.4%) as a pale-yellow solid.

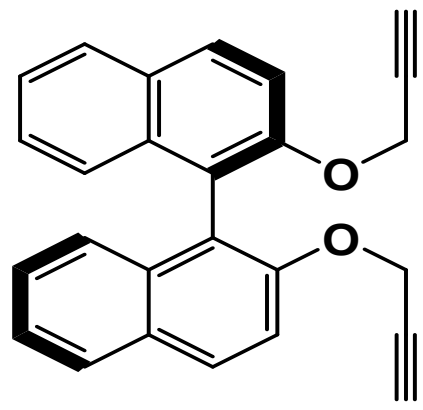

9

^{1}H-NMR: (CD_2Cl_2, 400 MHz) δ 8.05 (2H, d, *J* = 9.2 Hz), 7.93 (2H, d, *J* = 8.4 Hz), 7.61 (2H, d, *J* = 8.8 Hz), 7.39 (2H, t, *J* = 7.6 Hz), 7.27 (2H, t, *J* = 6.8 Hz), 7.11 (2H, d, *J* = 8.4 Hz), 4.68 (4H, s), 2.50(2H, s).

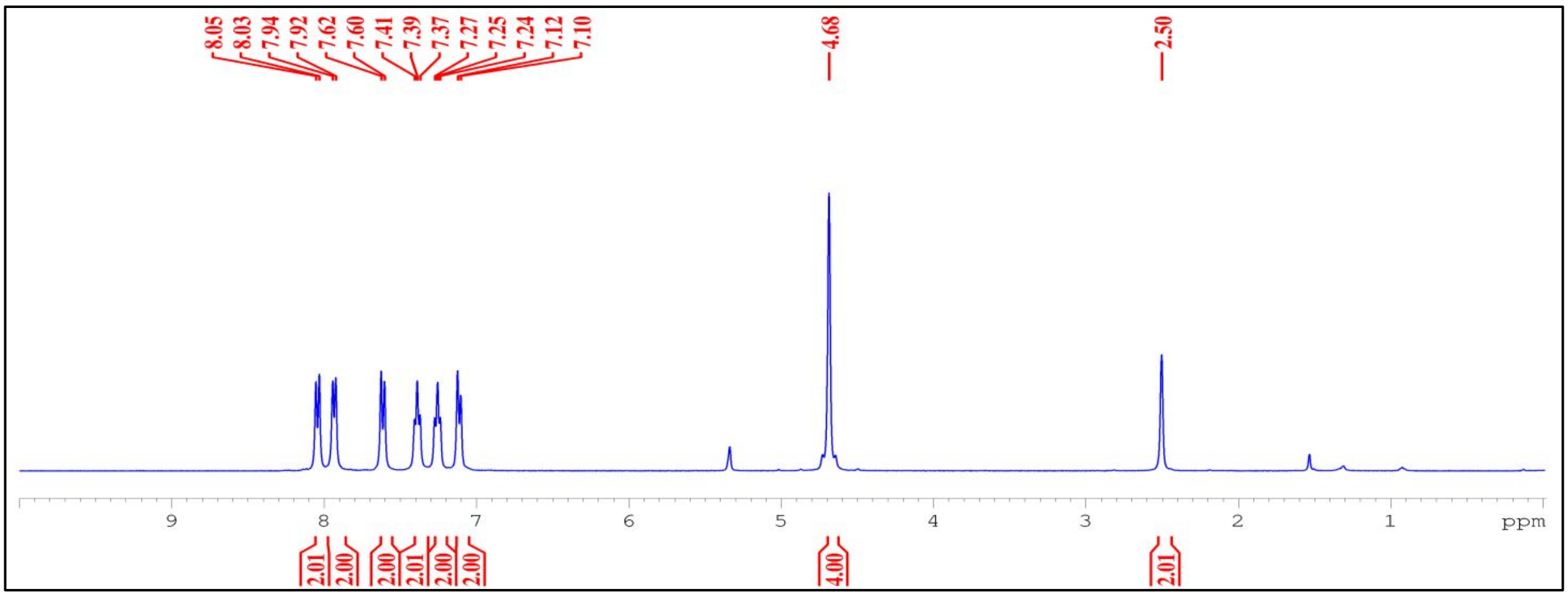

Figure S15. ^{1}H-NMR spectrum of compound 9.

^{13}C-NMR: (CD_2Cl_2, 100 MHz) δ 153.1, 133.9, 129.7, 129.4, 128.0, 126.5, 125.2, 124.1, 120.2, 115.5, 79.09, 75.1, 57.1.

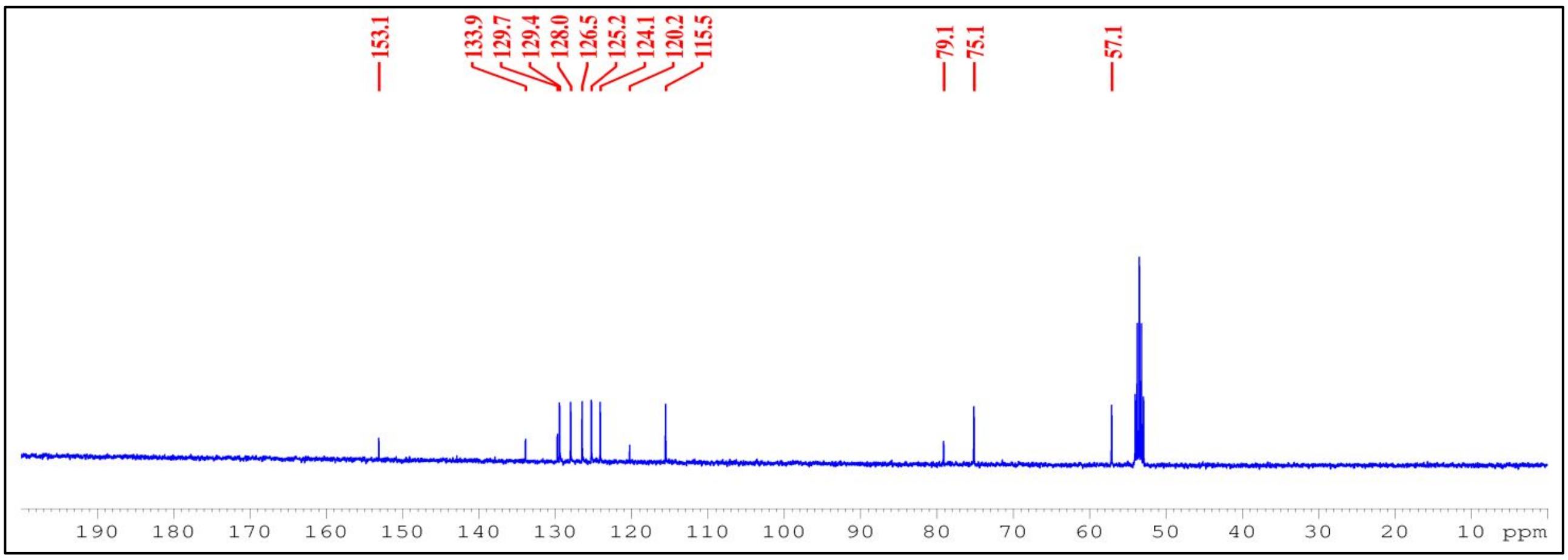

Figure S16. ^{13}C-NMR spectrum of compound 9.

(+)-MALDI-FTICR-MS ($CHCl_3$) m/z: $[M]^{\cdot+}$ Calcd for C26H18O2 362.1301; Found 362.1314.

- Compound **10** ((4^4Z,11^4Z)-1^2,2^2-diethoxy-4^1H,11^1H-6,9-dioxa-4(1,4),11(4,1)-ditriazola-1,2(1,6),7(2,1),8(1,2)-tetranaphthalenacyclododecaphane): $CuSO_4$ (63.5 mg, 2 eq., 0.397 mmol) and Sodium-L-Ascorbate (78.85 mg, 2 eq., 0.397 mmol) were dissolved at r.T. in 2 mL of a solution of H_2O/DMF, *v/v* 1/3, then compound **9** (72 mg, 1 eq., 0.199 mmol) was dissolved in 1.5 mL of DMF, added to the copper-ascorbate solution and stirred for 15 minutes at room temperature. Subsequently, compound **8** (90 mg, 1 eq., 0.199 mmol) was dissolved in 1.5 mL of DMF and added to the previous solution within compound **9**. Then the temperature increased at 60°C using a heating mantle and the reaction mixture stirred continuously for 24 hours. After cooling down the system, 20 mL of deionized H_2O were added to obtain a precipitate that was filtrated on a Gooch and washed with 40 mL of deionized H_2O. The solid was then recovered with 25 mL of $CHCl_3$, transferred into a separatory funnel and extracted with H_2O (3 x 25 mL). The organic phases were collected, dried over Na_2SO_4, filtrated and concentrated to afford 100 mg of impure yellowish product **10** that was further purified by means of a semipreparative TLC with the following conditions: TLC plate of 2 mm thickness submitted to 4 elution using 2 x 200 mL of $CHCl_3$/MeOH

v/v 99.5/0.5 plus 2 x 200 mL of $CHCl_3$/MeOH *v/v* 99.0/1.0. The macrocycle **10** had an R_F equal to 0.26 and was obtained as a white solid in a Yield of 9.4% (15.6 mg).

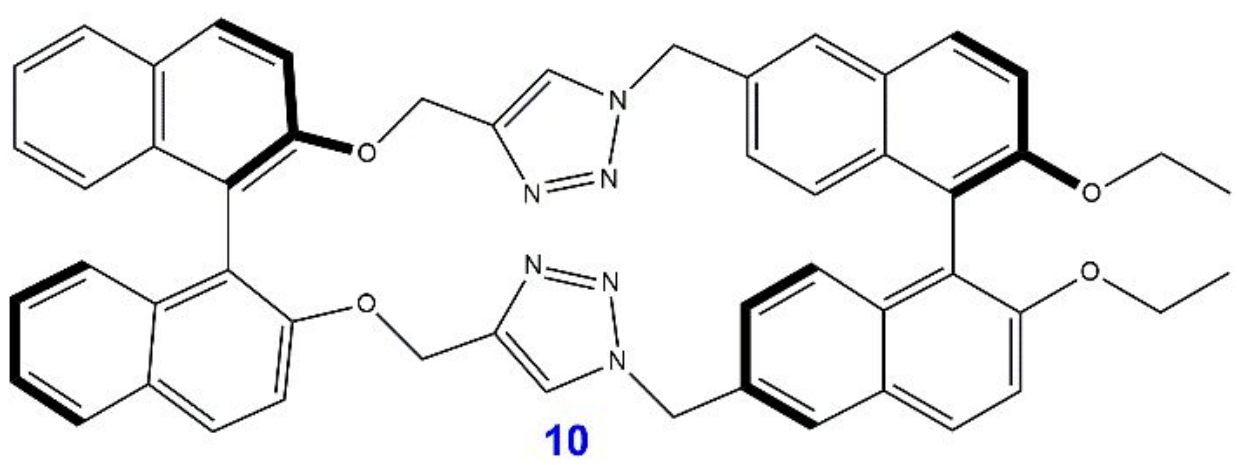

10

^{1}H-NMR: (500 MHz, DMSO-d_6) δ 7.80 – 7.72 (m, 1H), 7.66 – 7.63 (s, 1H), 7.53 – 7.49 (s, 1H), 7.39 – 7.36 (s, 0H), 7.05 – 7.02 (s, 1H), 6.93 – 6.89 (s, 1H), 6.79 – 6.75 (s, 1H), 5.48 – 5.41 (d, *J* = 17.7 Hz, 2H), 5.03 – 4.93 (m, 1H), 3.97 – 3.93 (s, 1H), 0.88 – 0.85 (s, 1H).

^{13}C-NMR: δ 128.9, 126.8, 122.9, 125.7, 122.9, 114.8, 63.7, 62.0, 52.2, 12.8.

(+)-MALDI-FTICR-MS ($CHCl_3$) m/z: $[M + H]^+$ Calcd for C52H42N6O4H 815.3340; Found 815.3347

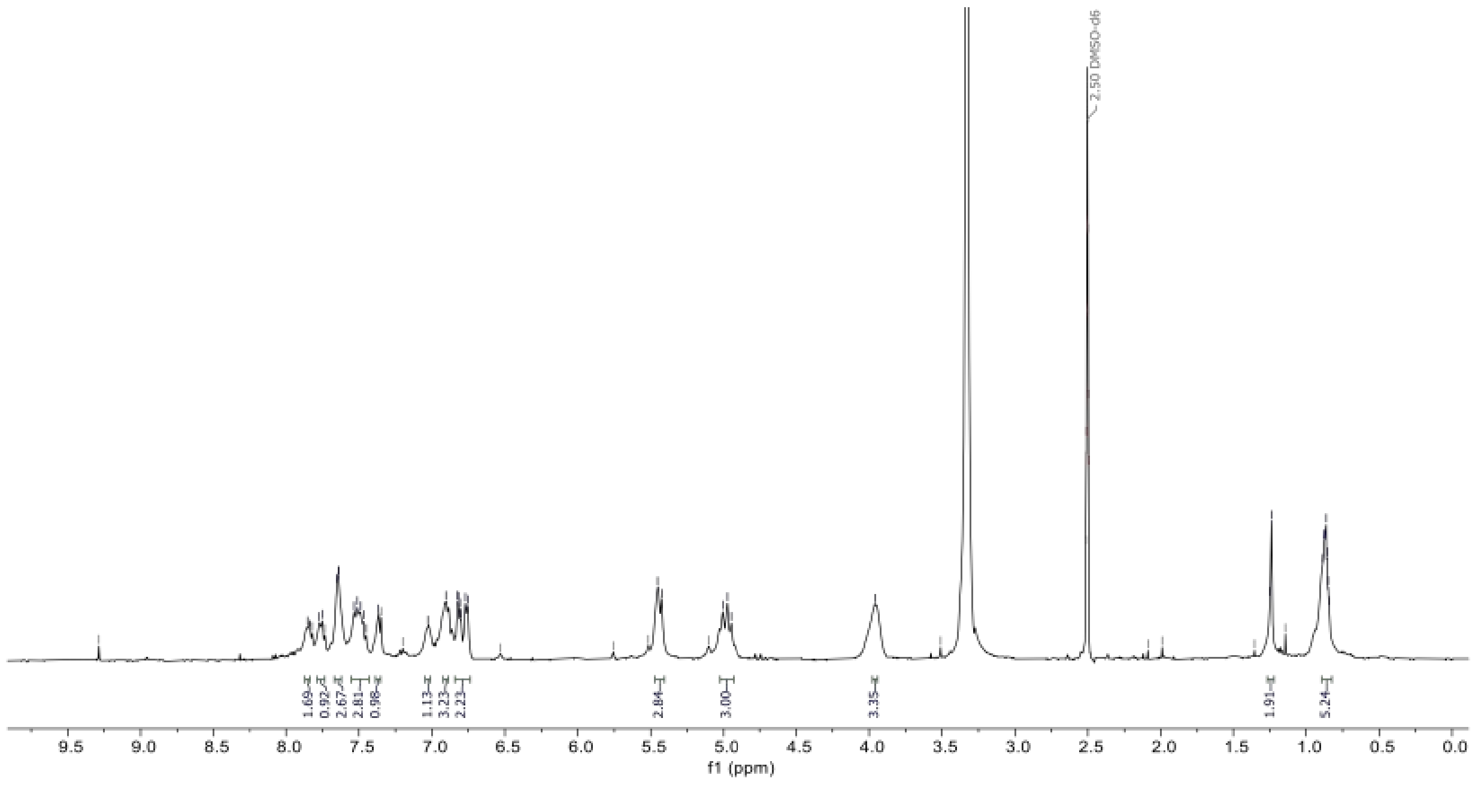

Figure S17: ^{1}H NMR spectrum of compound **5** in DMSO-d_6.

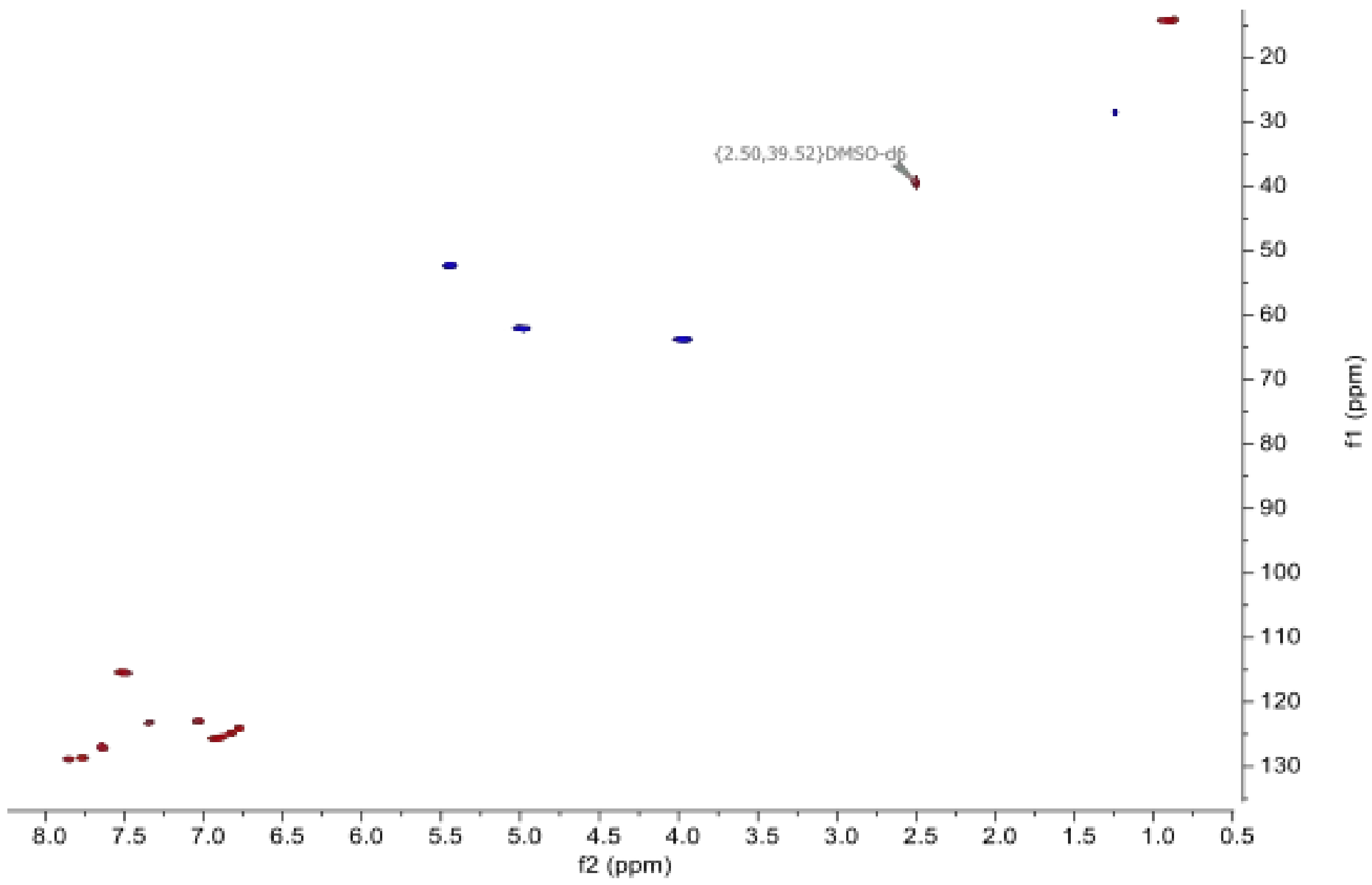

Figure S18: HSQC spectrum of compound **5** in DMSO-d_6.

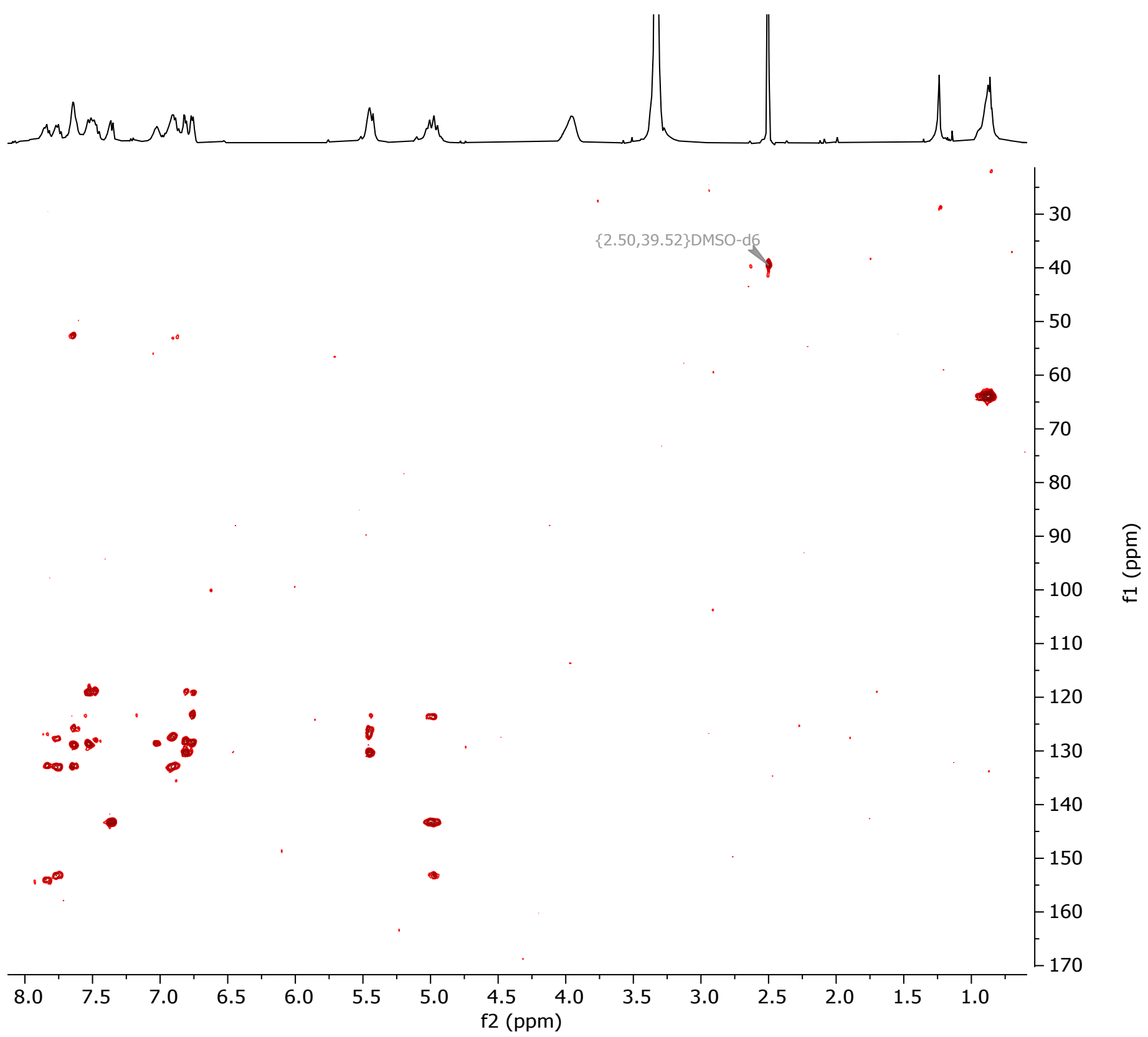

Figure S19: HMBC spectrum of compound **5** in DMSO-d_6.

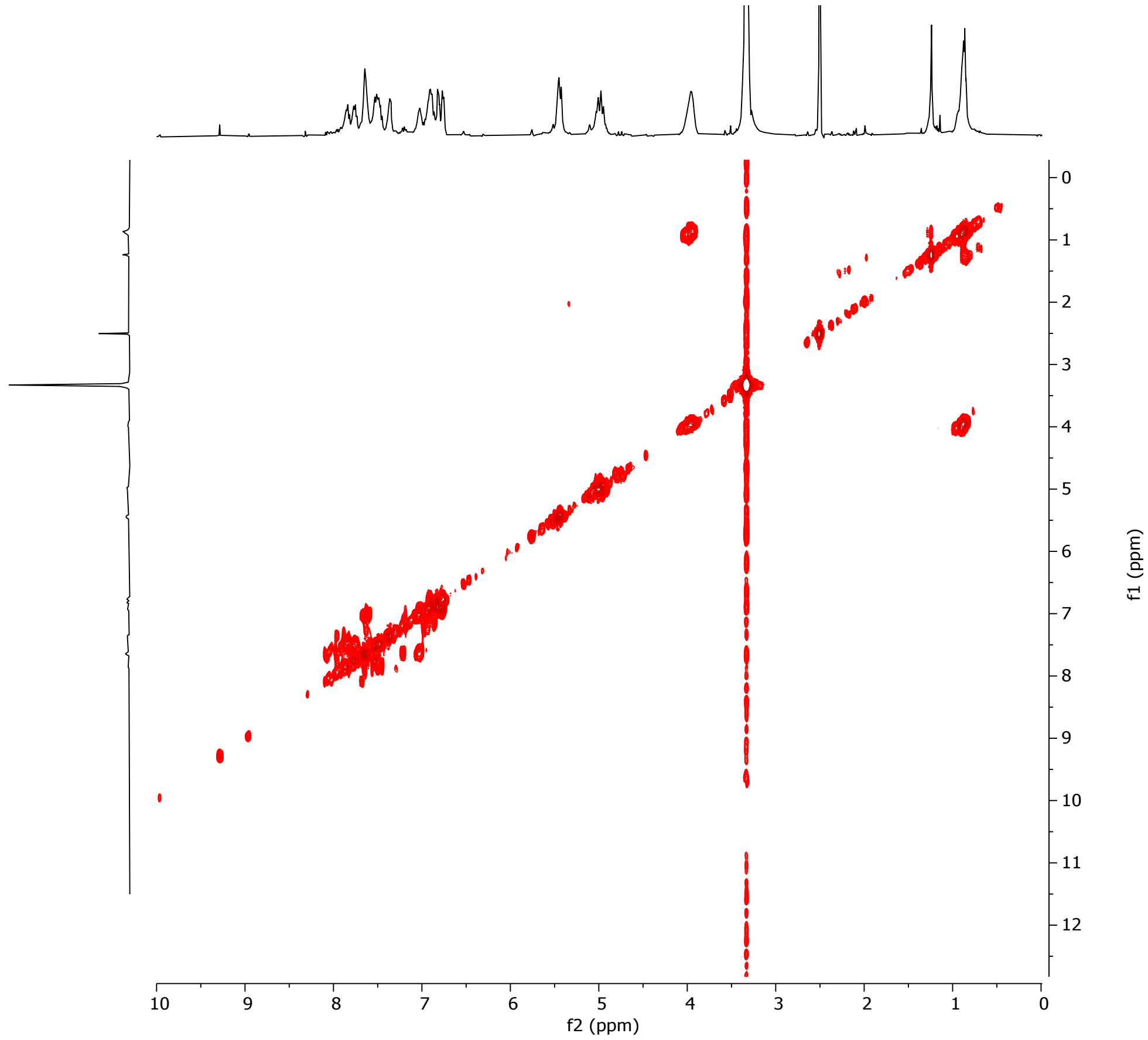

Figure S20: 2D-COSY spectrum of compound **5** in DMSO-d_6.

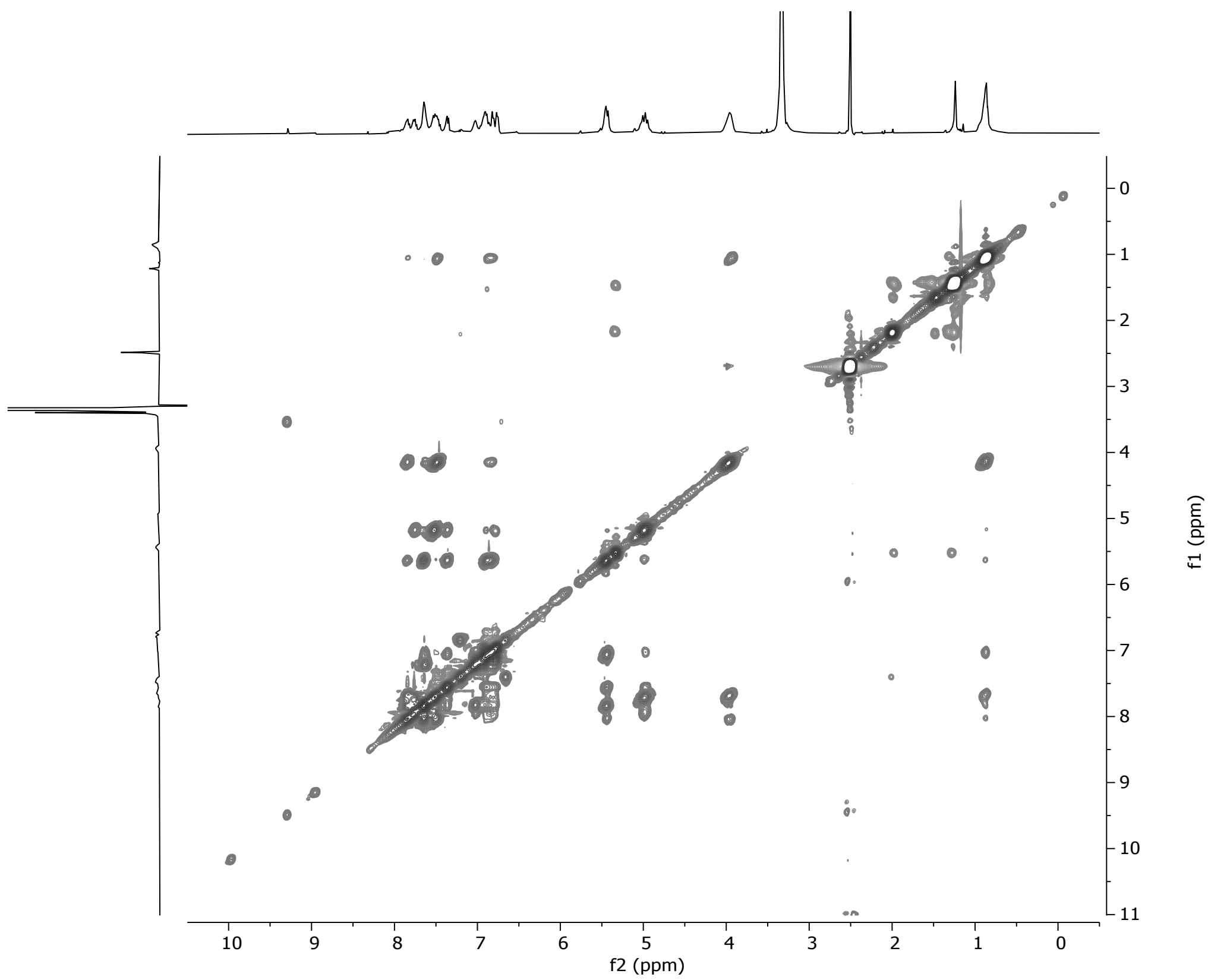

Figure S21: 2D-NOESY spectrum of compound **5** in DMSO-d_6.

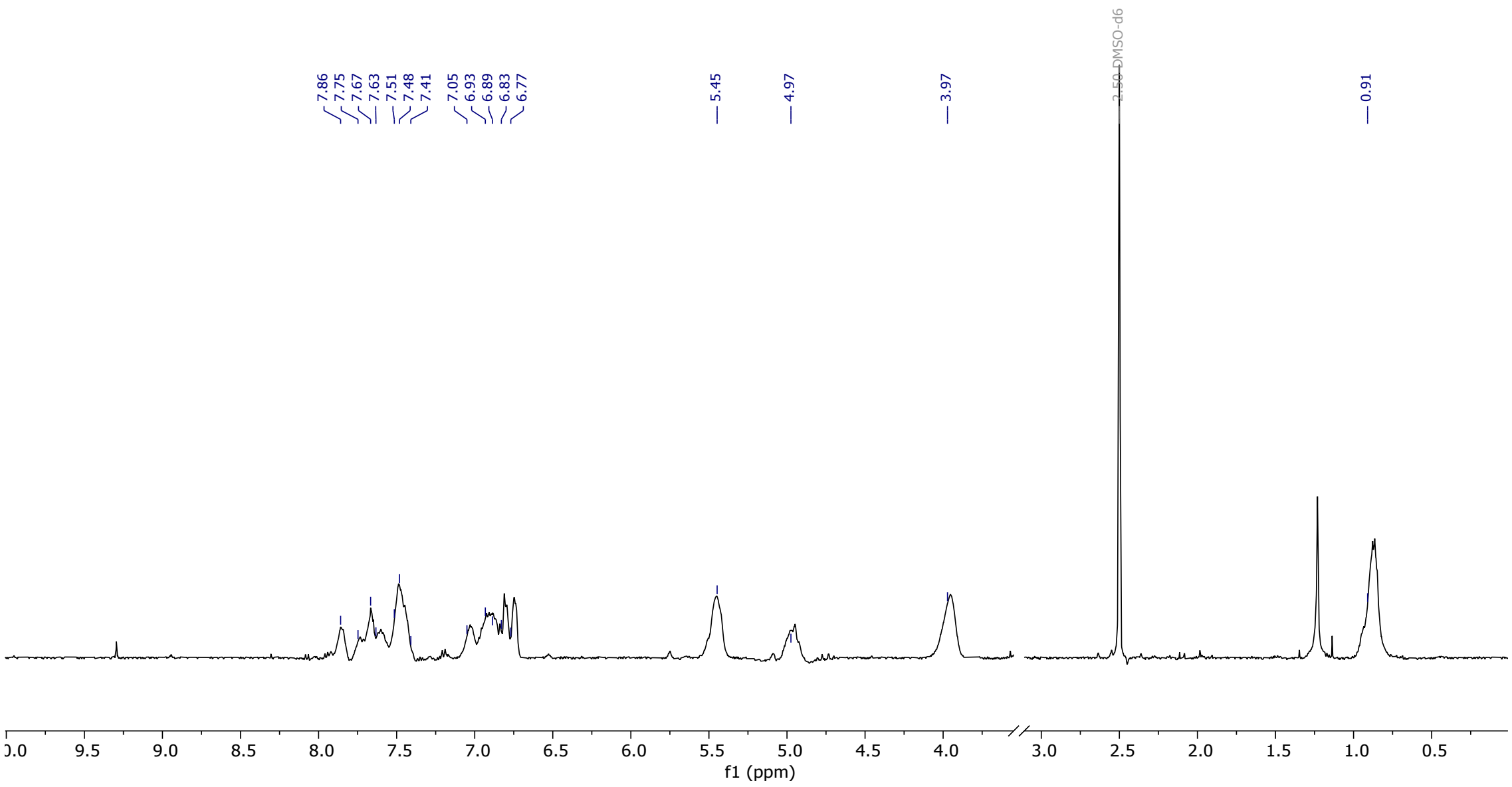

Figure S22: ^{1}H NMR spectrum of compound **5** in DMSO-d_6 after addition of 1 equiv. of $AgNO_3$.

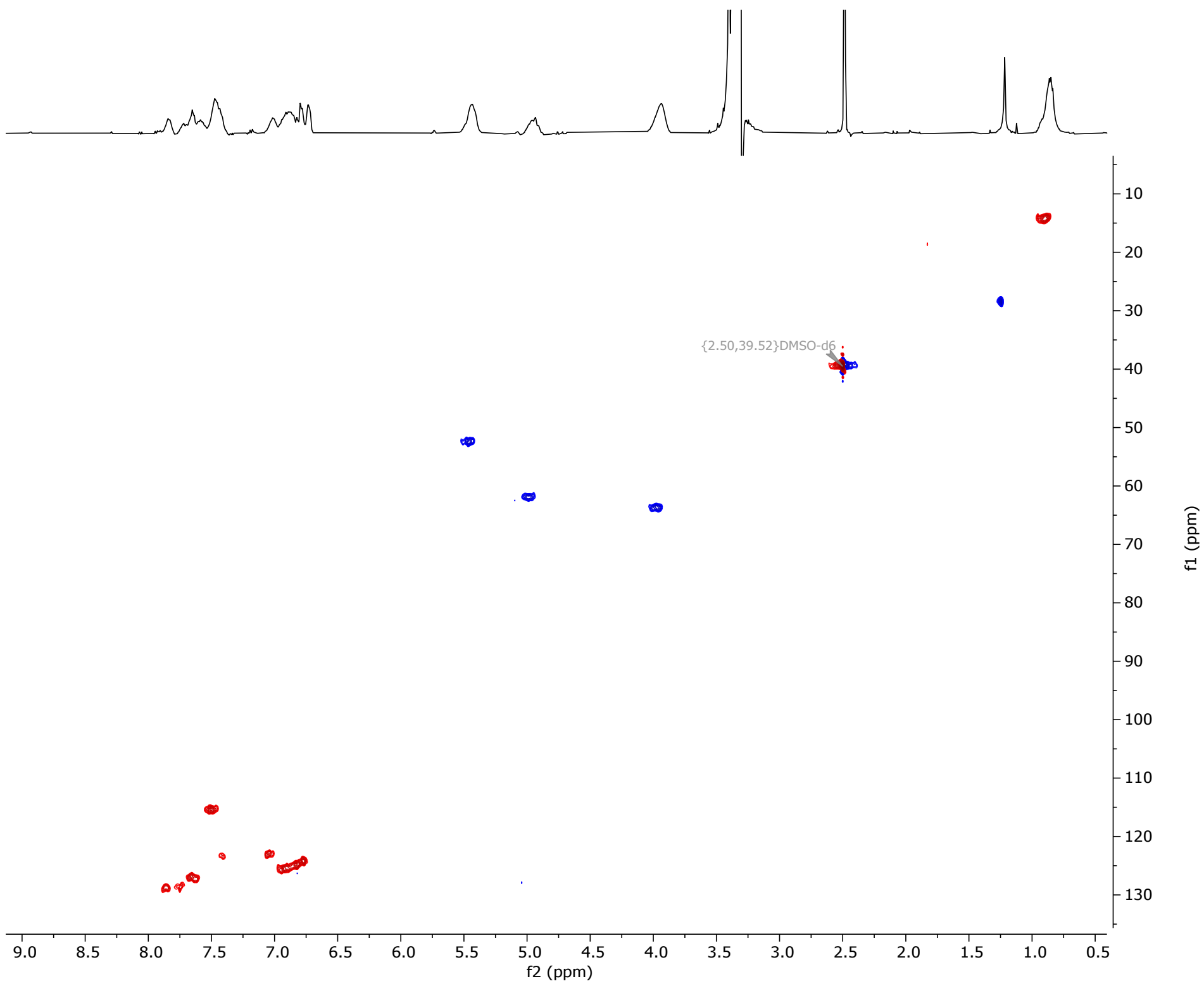

Figure S23: HSQC spectrum of compound **5** in DMSO-d_6 after addition of 1 equiv. of $AgNO_3$.

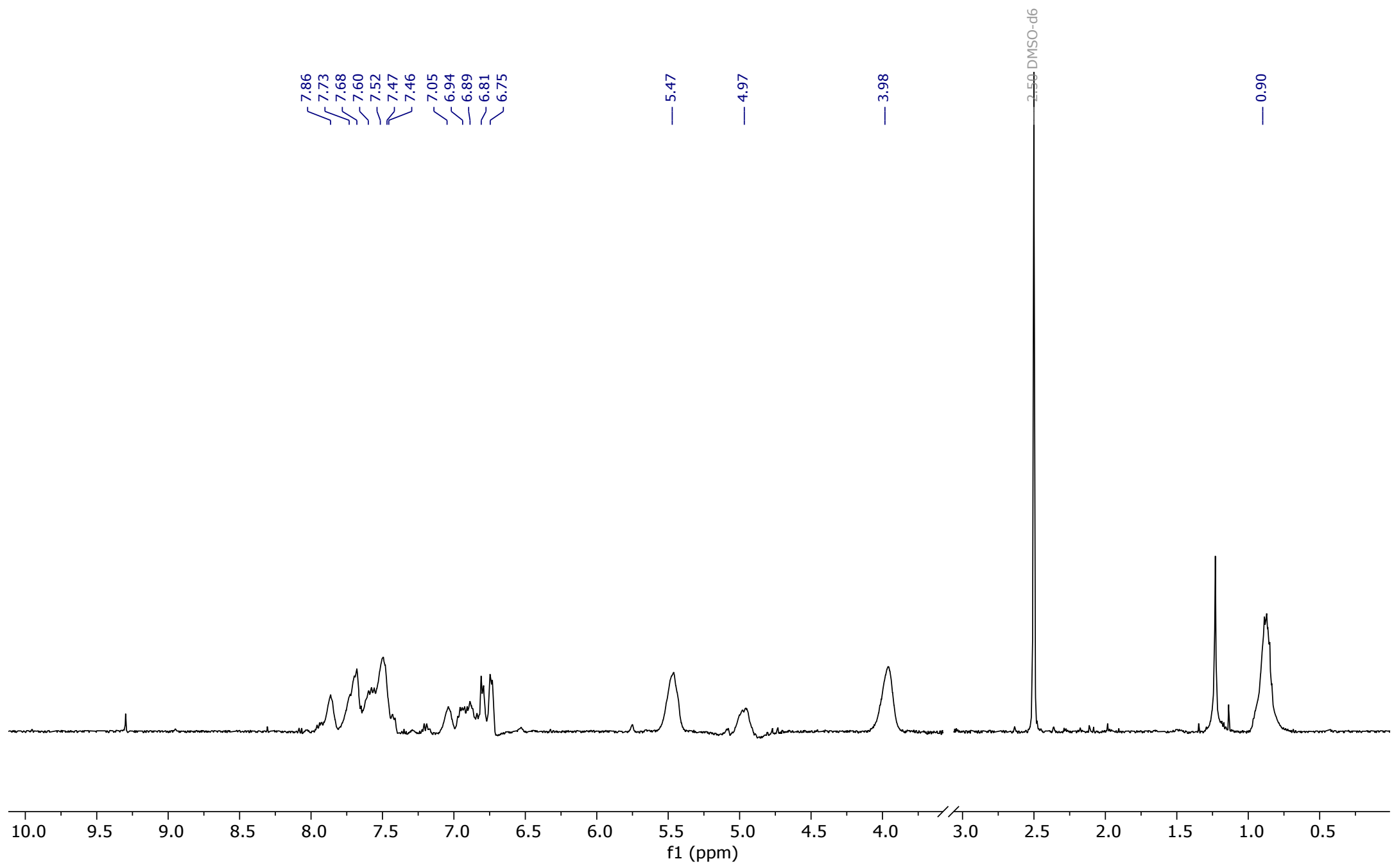

Figure S24: ^{1}H NMR spectrum of compound **5** in DMSO-d_6 after addition of 2 equiv. of $AgNO_3$.

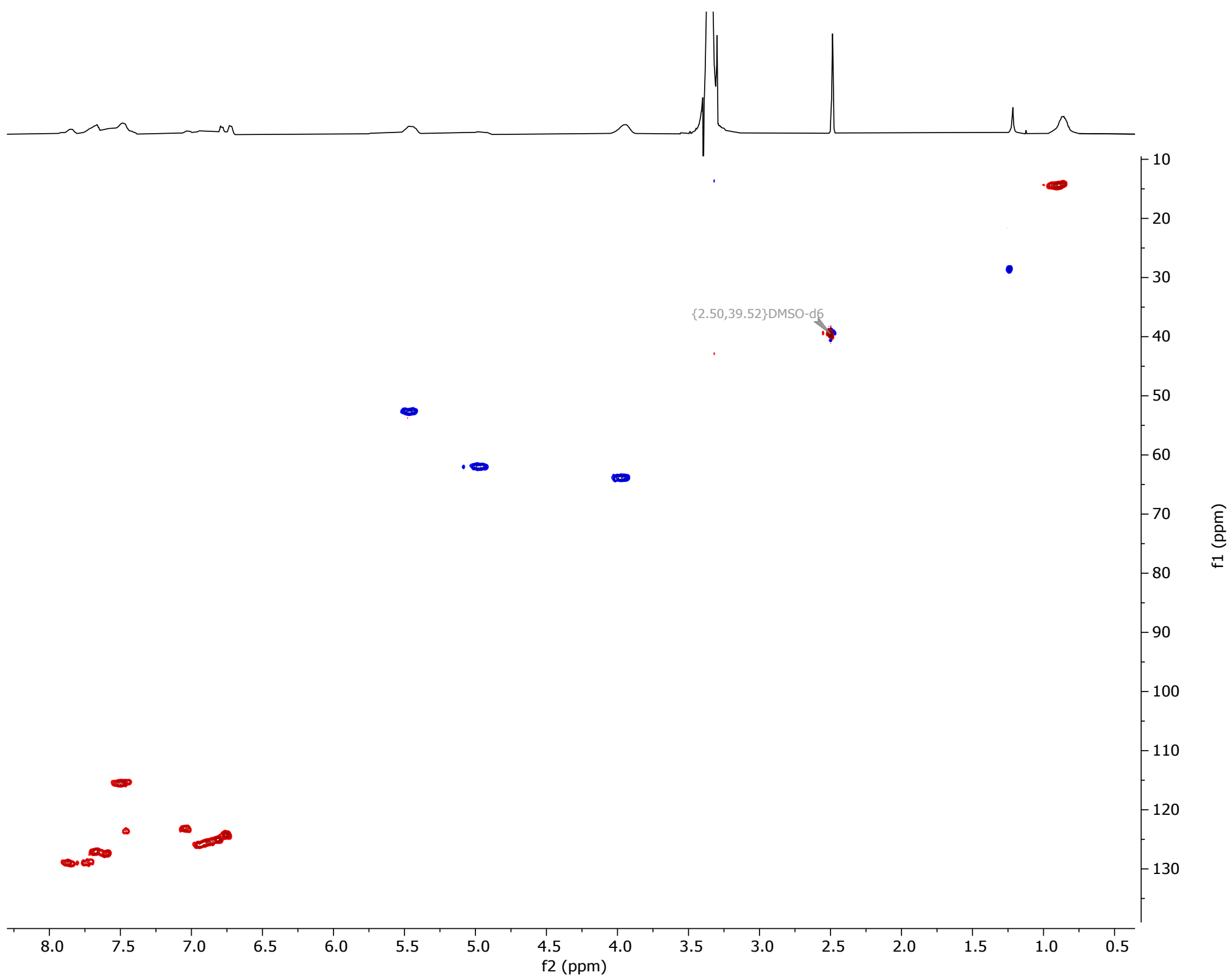

Figure S25: HSQC spectrum of compound **5** in DMSO-d_6 after addition of 2 equiv. of $AgNO_3$.

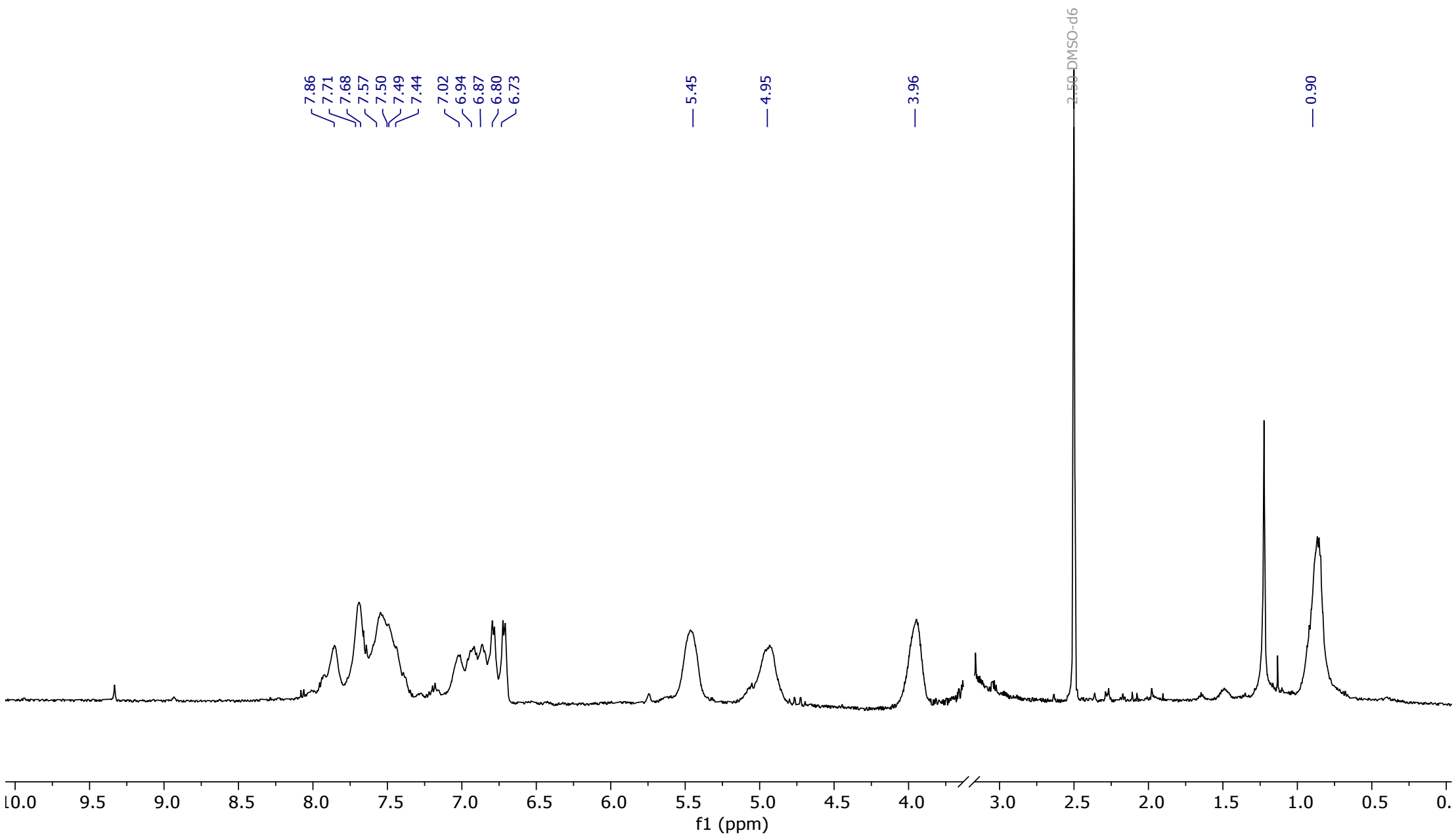

Figure S26: ^{1}H NMR spectrum of compound **5** in DMSO-d_6 after addition of 3 equiv. of $AgNO_3$.

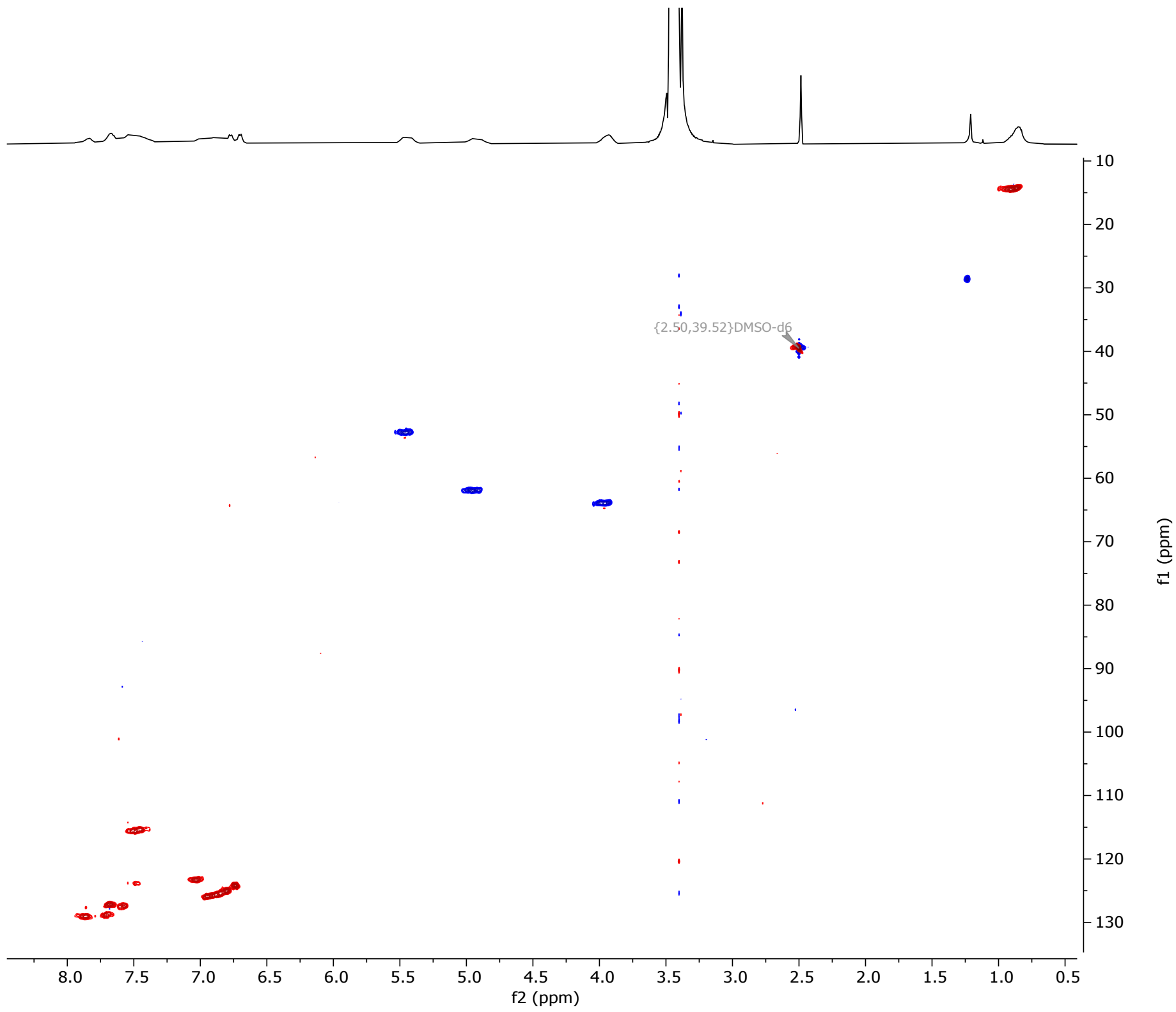

Figure S27: HSQC spectrum of compound **5** in DMSO-d_6 after addition of 3 equiv. of $AgNO_3$.

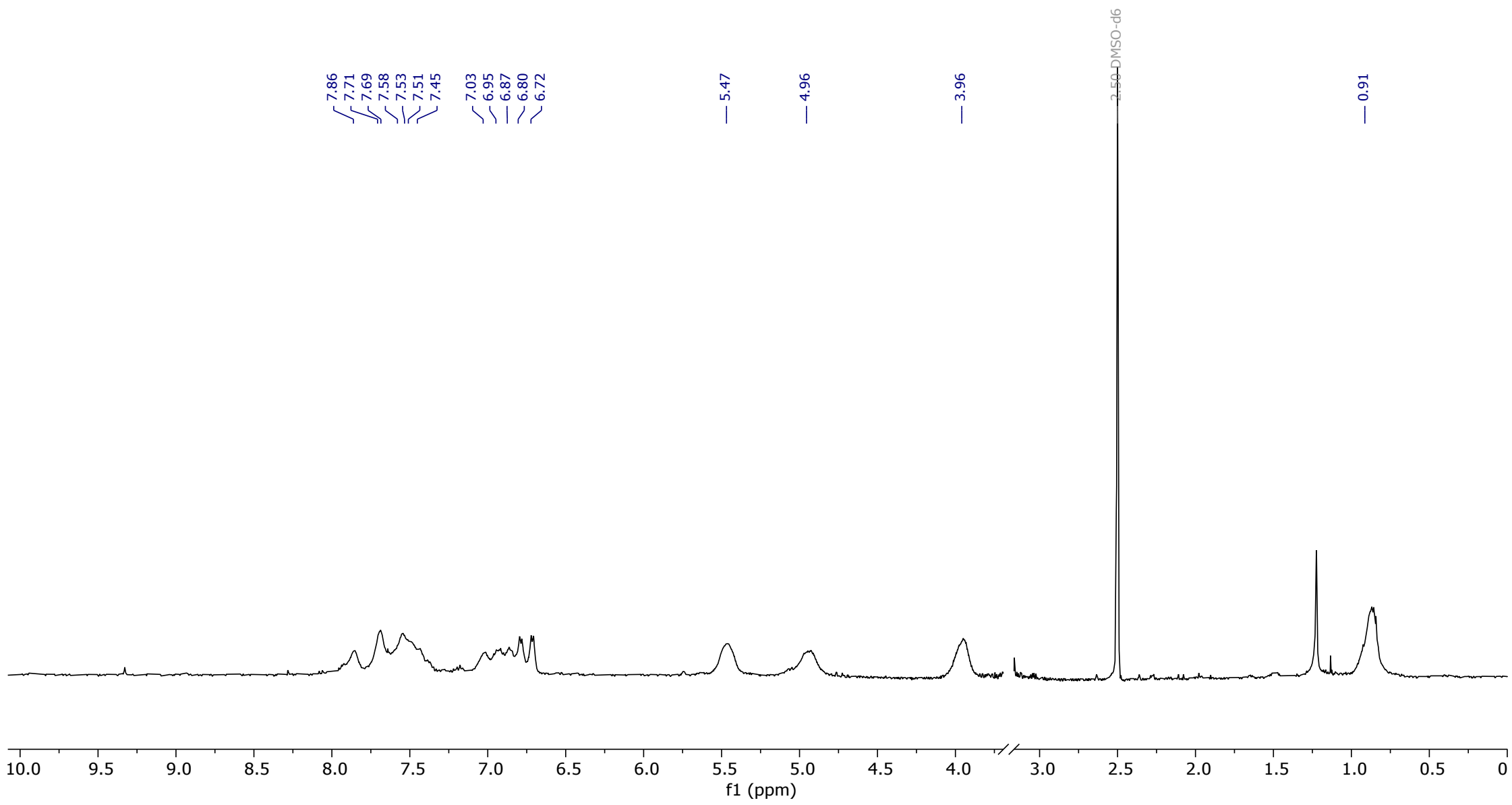

Figure S28: ^{1}H NMR spectrum of compound **5** in DMSO-d_6 after addition of 4 equiv. of $AgNO_3$.

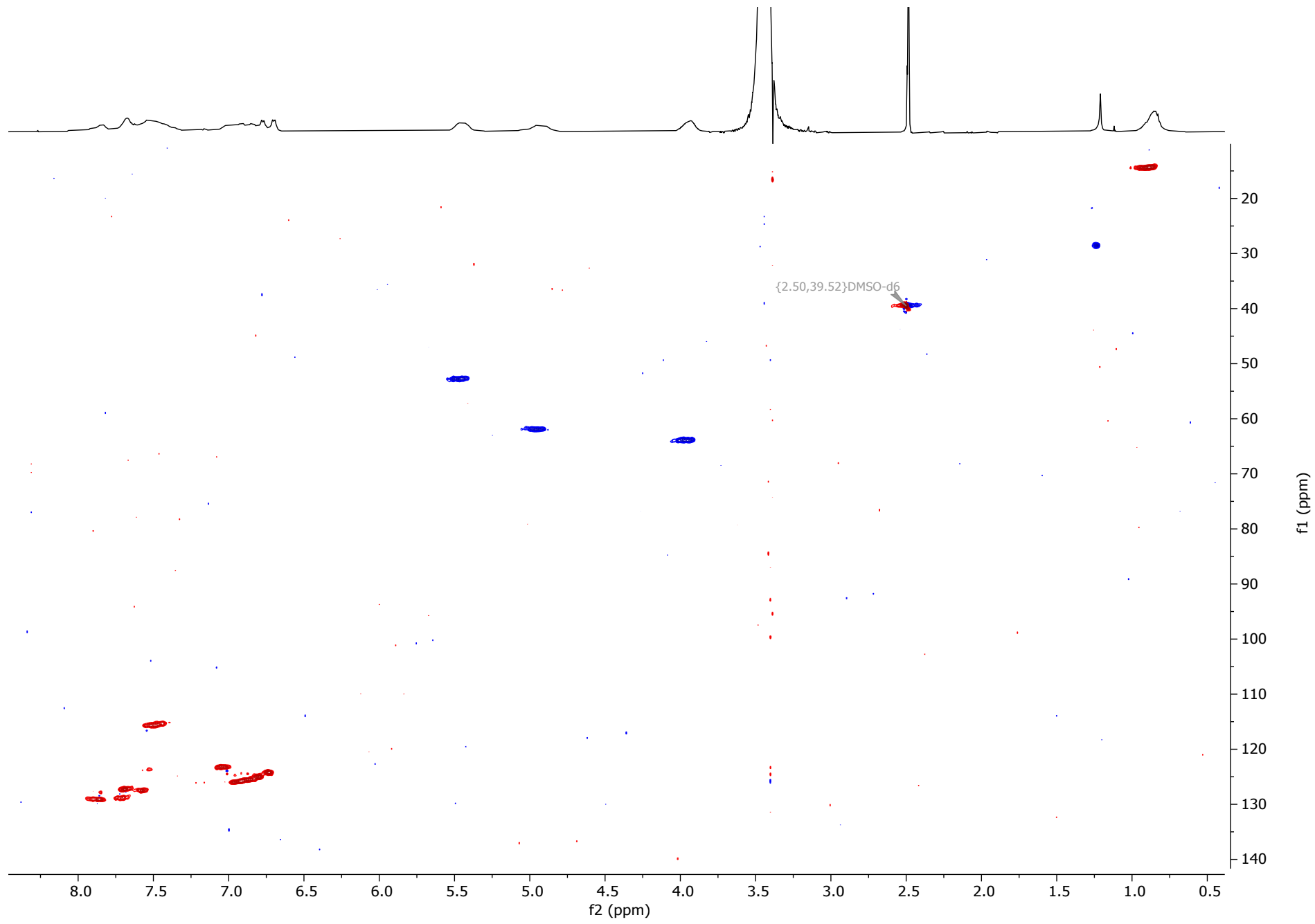

Figure S29: HSQC spectrum of compound **5** in DMSO-d_6 after addition of 4 equiv. of $AgNO_3$.

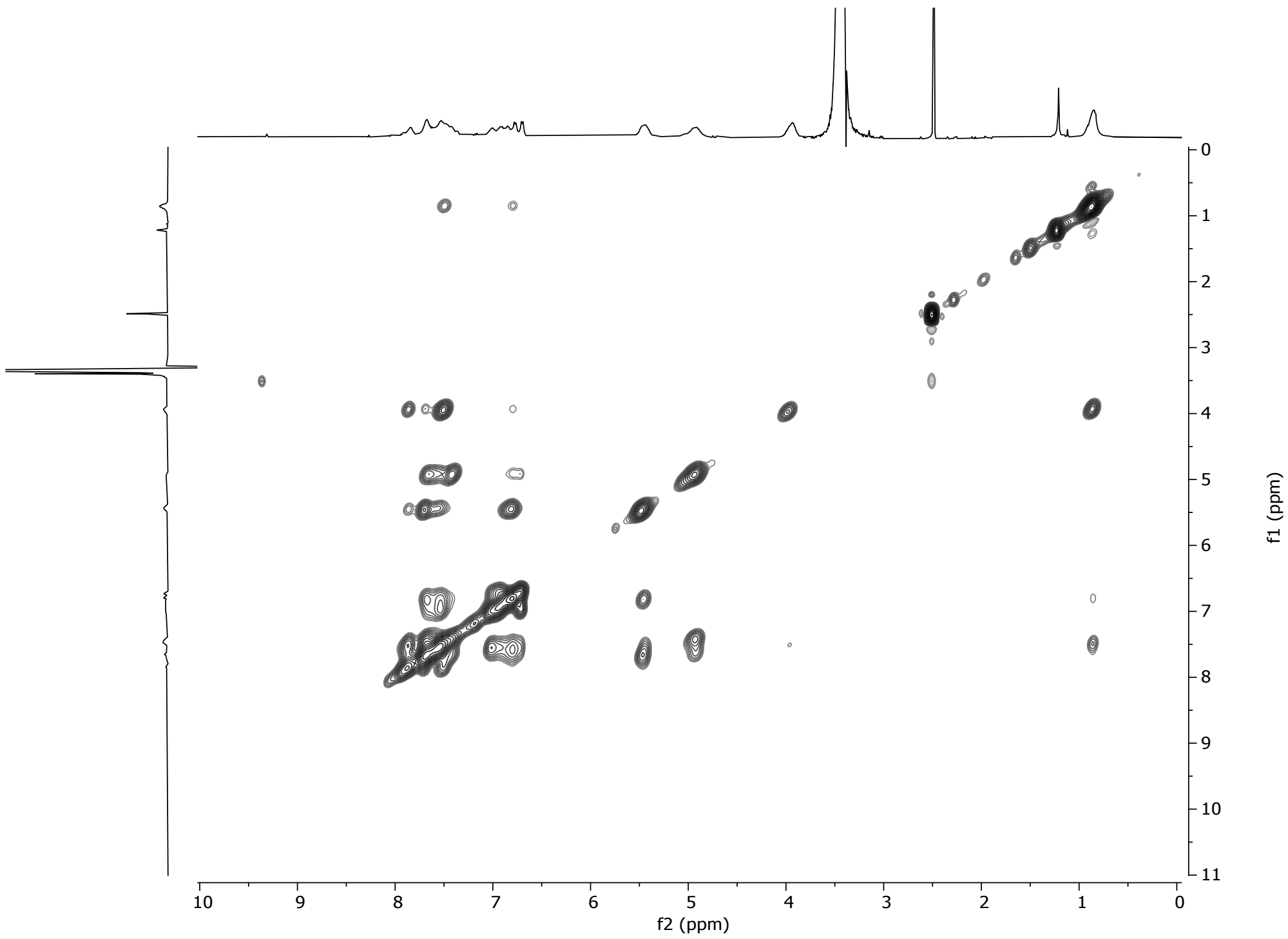

Figure S30: 2D-NOESY spectrum of compound **5** in DMSO-d_6 after addition of 4 equiv. of $AgNO_3$.

Table S1: ^{1}H and ^{13}C chemical shifts of compound **5** in DMSO-d_6 in absence and in presence of 1, 2, 3, 4 equiv. of $AgNO_3$.

**^{1}H chemical shift (ppm)**						**^{13}C chemical shift (ppm)**				
**Peaks**	**5**	**5:** $AgNO_3$ **1:1**	**5:** $AgNO_3$ **1:2**	**5:** $AgNO_3$ **1:3**	**5:** $AgNO_3$ **1:4**	**5**	**5:** $AgNO_3$ **1:1**	**5:** $AgNO_3$ **1:2**	**5:** $AgNO_3$ **1:3**	**5:** $AgNO_3$ **1:4**
**21**	7.86	7.86	7.86	7.86	7.86	128.9	128.9	128.8	129.1	129.1
**6**	7.77	7.75	7.73	7.71	7.71	128.6	128.8	128.8	128.7	128.7
**7**	7.66	7.67	7.68	7.68	7.69	127.0	127.0	127.0	127.1	127.2
**20**	7.63	7.63	7.60	7.57	7.58	127.1	127.3	127.5	127.4	127.3
**5**	7.54	7.52	7.52	7.49	7.51	115.4	115.5	115.5	115.3	115.6
**22**	7.49	7.48	7.47	7.44	7.45	115.4	115.6	115.5	115.6	115.3
**13**	7.35	7.41	7.45	7.49	7.53	123.3	123.6	123.6	124.0	123.6
**8**	7.04	7.05	7.05	7.03	7.03	123.1	123.1	123.1	123.4	123.1
**9**	6.94	6.93	6.94	6.93	6.95	125.7	125.7	125.9	125.9	125.7
**17**	6.89	6.89	6.89	6.87	6.87	125.5	125.4	125.4	125.6	125.4
**16**	6.83	6.83	6.81	6.80	6.80	125.0	125.1	125.1	125.1	124.9
**10**	6.79	6.77	6.75	6.74	6.73	124.1	124.2	124.1	124.2	124.3
**14**	5.45	5.45	5.47	5.45	5.47	52.3	52.6	52.8	52.7	52.7
**11**	4.99	4.98	4.97	4.95	4.95	62.1	61.9	61.9	62.0	61.9
**25**	3.96	3.97	3.98	3.96	3.96	63.7	64.3	63.9	63.8	63.8
**26**	0.90	0.91	0.90	0.90	0.91	14.1	14.7	14.1	14.1	14.1

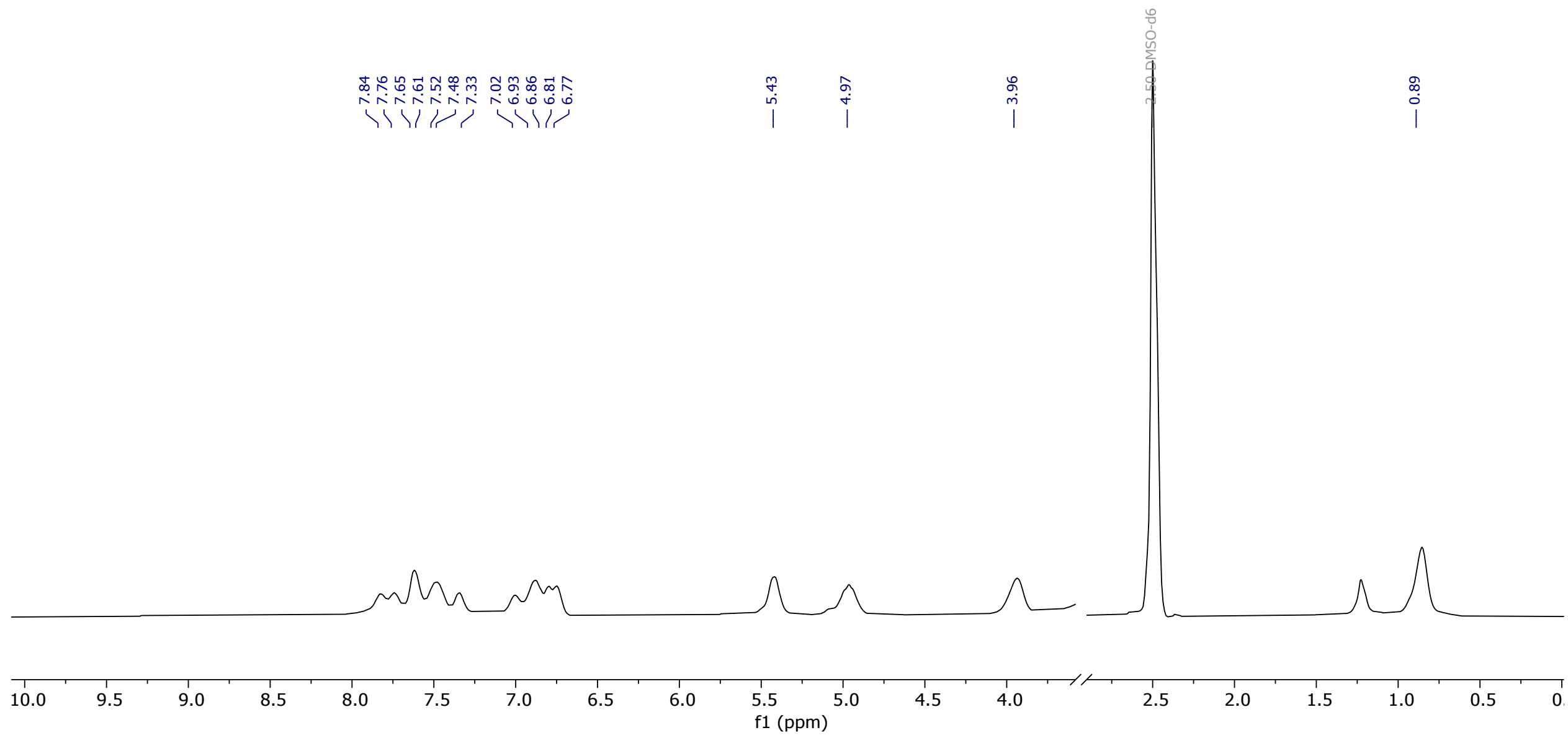

Figure S31: ^{1}H NMR spectrum of compound **5** in DMSO-d_6 after addition of 1 equiv. of $CoCl_2$.

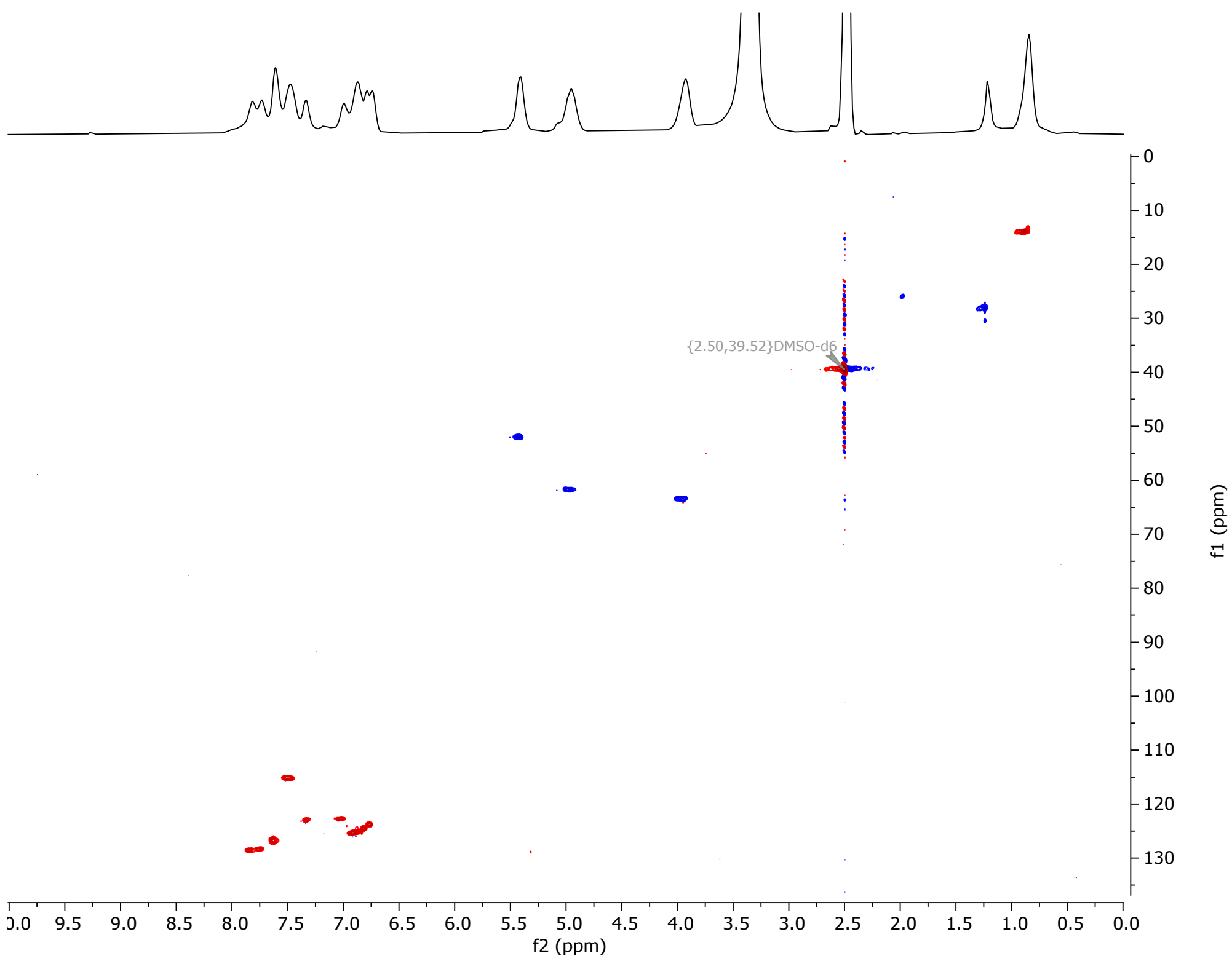

Figure S32: HSQC spectrum of compound **5** in DMSO-d_6 after addition of 1 equiv. of $CoCl_2$.

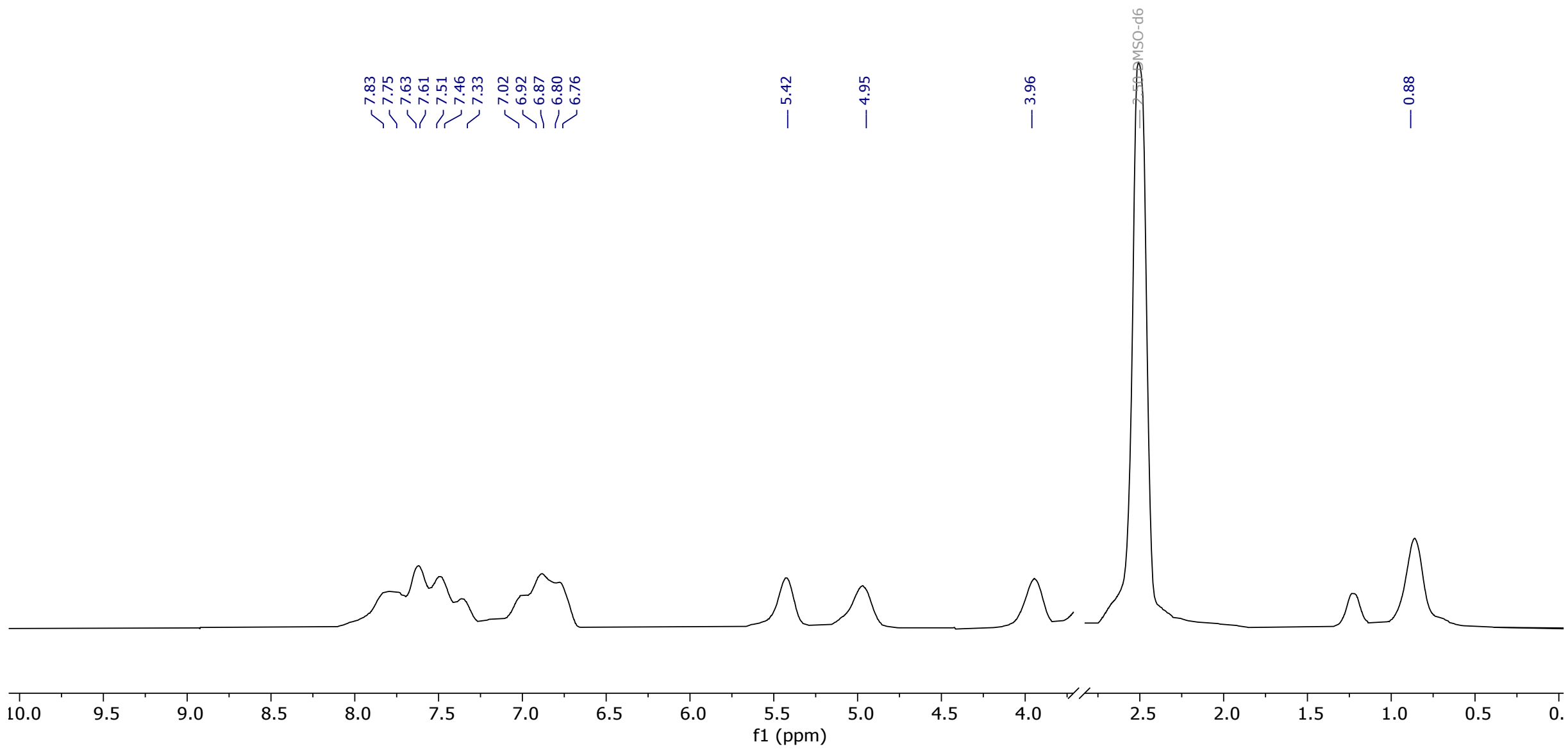

Figure S33: ^{1}H NMR spectrum of compound **5** in DMSO-d_6 after addition of 2 equiv. of $CoCl_2$.

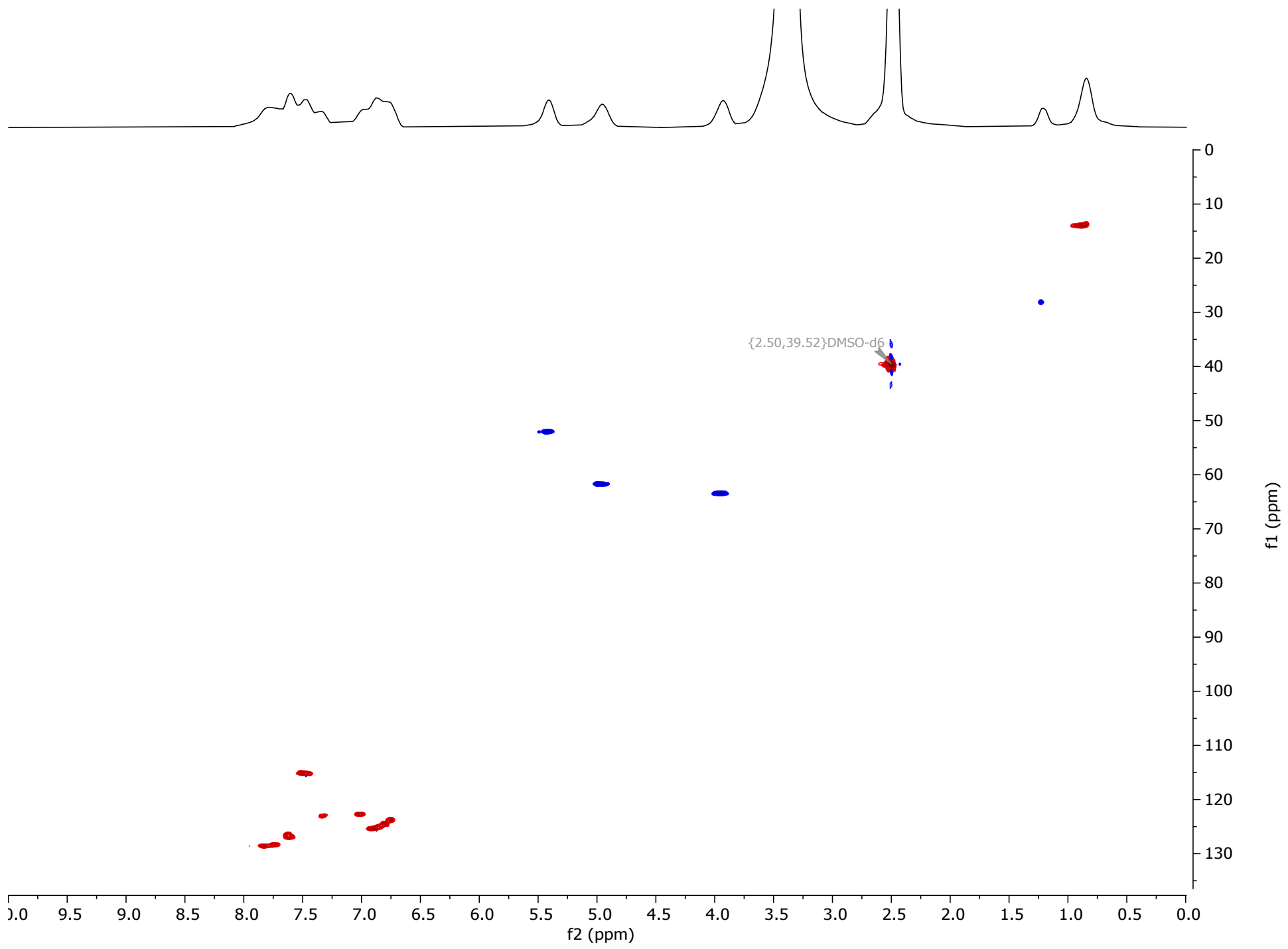

Figure S34: HSQC spectrum of compound **5** in DMSO-d_6 after addition of 2 equiv. of $CoCl_2$.

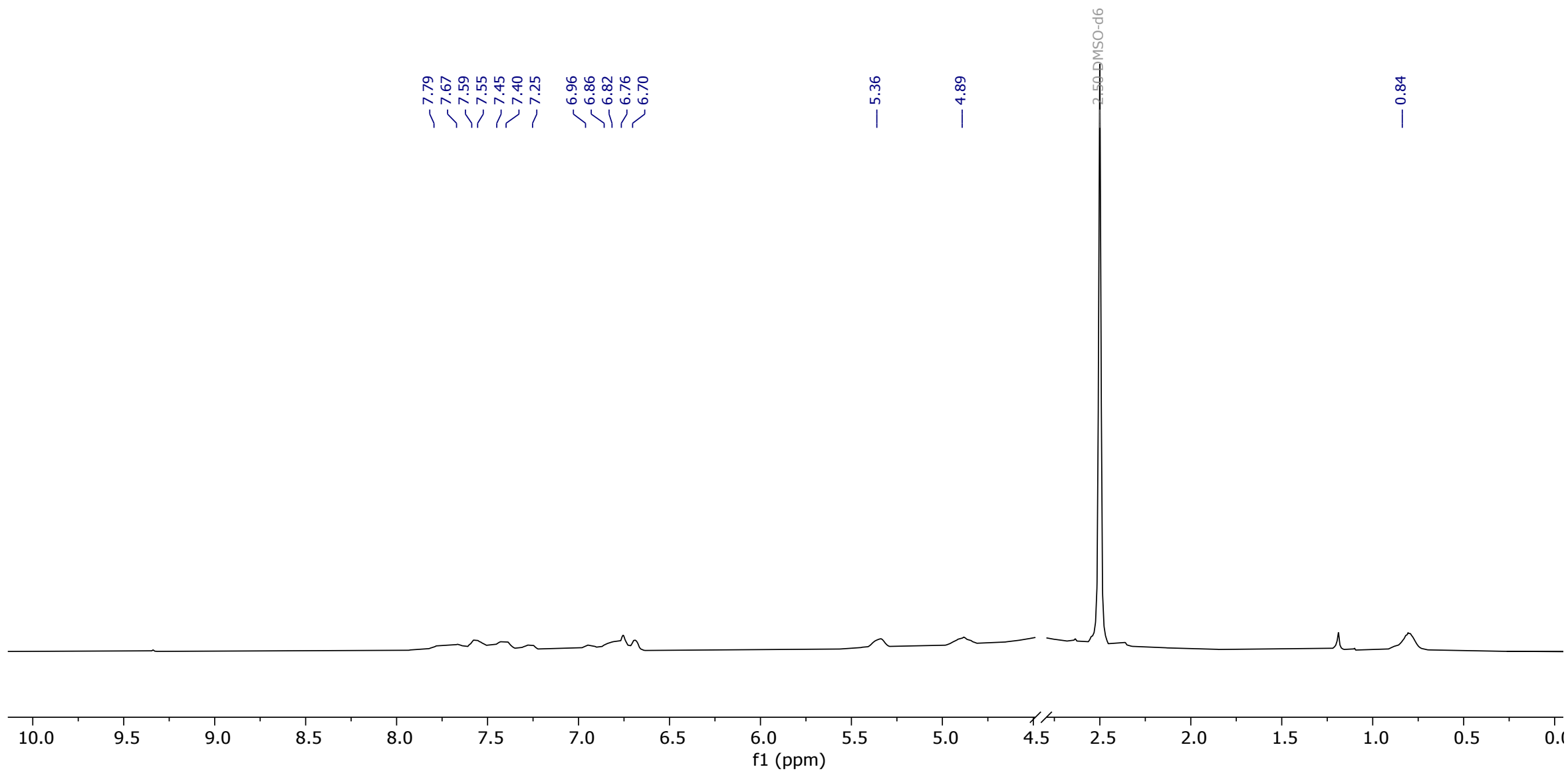

Figure S35: ^{1}H NMR spectrum of compound **5** in DMSO-d_6 after addition of 3 equiv. of $CoCl_2$.

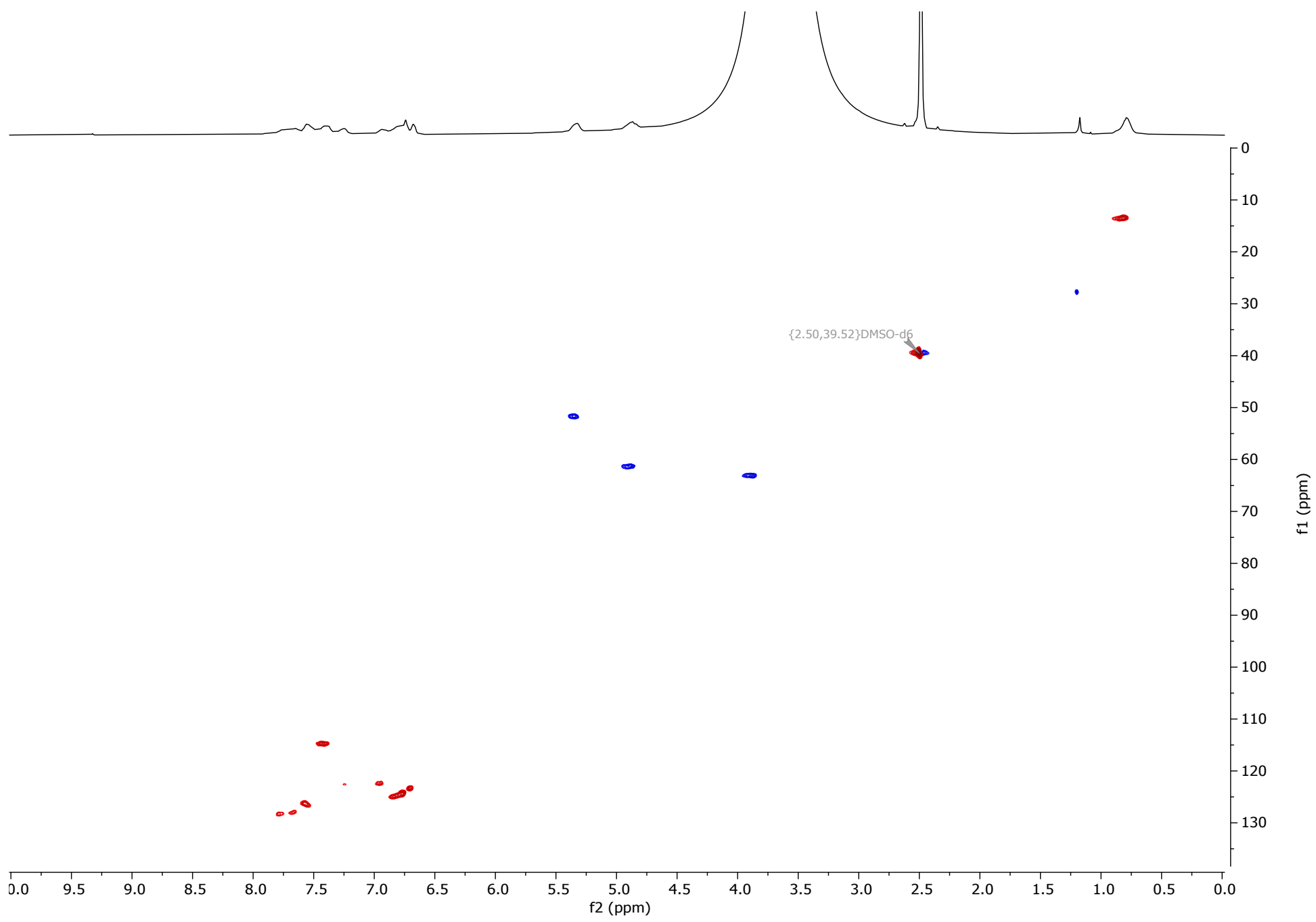

Figure S36: HSQC spectrum of compound **5** in DMSO-d_6 after addition of 3 equiv. of $CoCl_2$.

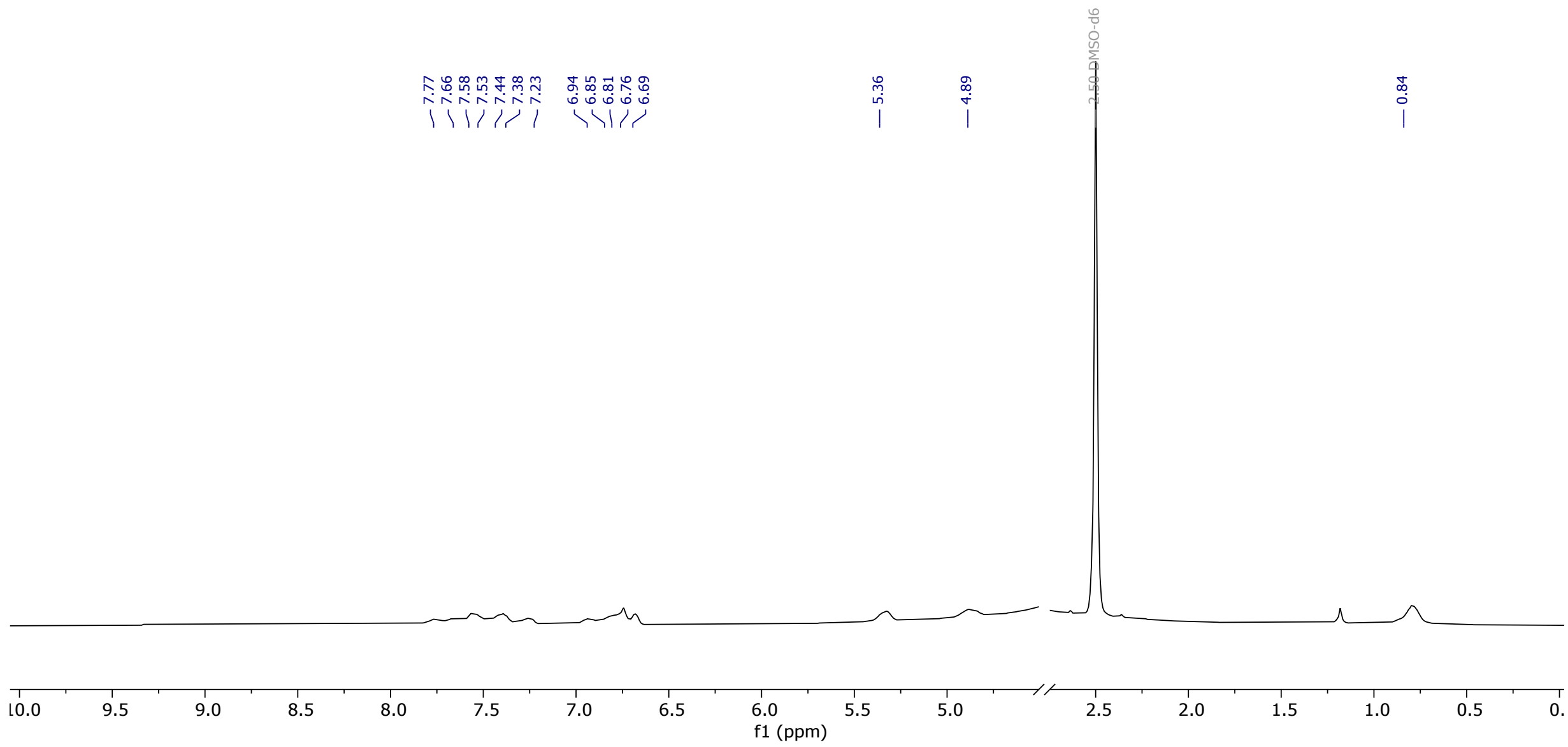

Figure S37: ^{1}H NMR spectrum of compound **5** in DMSO-d_6 after addition of 4 equiv. of $CoCl_2$.

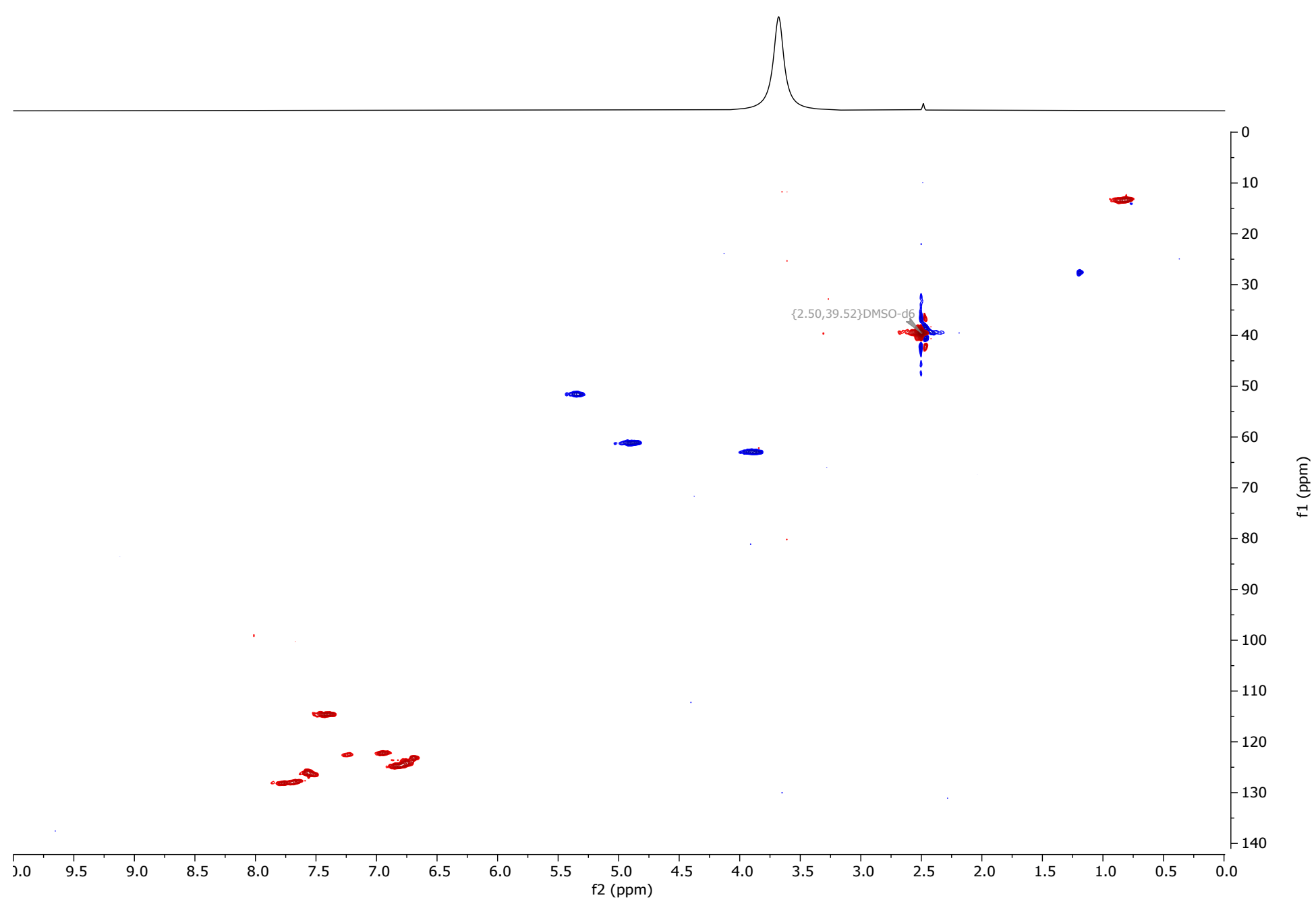

Figure S38: HSQC spectrum of compound **5** in DMSO-d_6 after addition of 4 equiv. of $CoCl_2$.

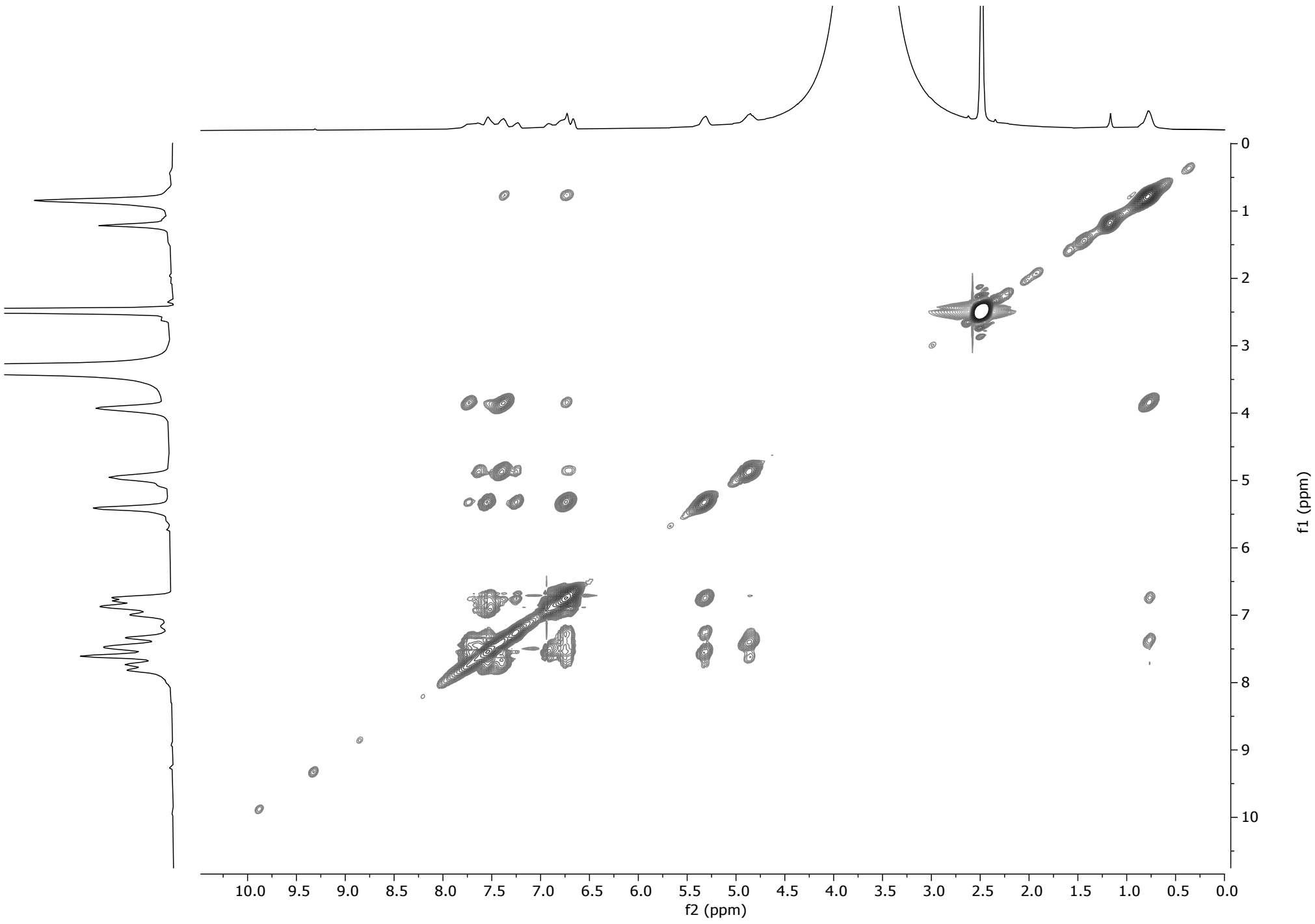

Figure S39: 2D-NOESY spectrum of compound **5** in DMSO-d_6 after addition of 4 equiv. of $CoCl_2$.

Table S2: ^{1}H and ^{13}C chemical shifts of compound **5** in DMSO-d_6 in absence and in presence of 1, 2, 3, 4 equiv. of $CoCl_2$.

**^{1}H chemical shift (ppm)**						**^{13}C chemical shift (ppm)**				
**Peaks**	**5**	**5: CoCl$_2$ 1:1**	**5: CoCl$_2$ 1:2**	**5: CoCl$_2$ 1:3**	**5: CoCl$_2$ 1:4**	**5**	**5: CoCl$_2$ 1:1**	**5: CoCl$_2$ 1:2**	**5: CoCl$_2$ 1:3**	**5: CoCl$_2$ 1:4**
**21**	7.86	7.84	7.83	7.79	7.77	128.9	128.6	128.5	128.3	128.1
**6**	7.77	7.76	7.75	7.67	7.66	128.6	128.4	128.2	128.1	127.8
**7**	7.66	7.64	7.63	7.59	7.58	127.0	126.7	126.4	126.2	125.9
**20**	7.63	7.61	7.61	7.55	7.53	127.1	126.8	126.7	126.6	126.3
**5**	7.54	7.52	7.51	7.45	7.44	115.4	114.9	114.9	114.6	114.6
**22**	7.49	7.48	7.46	7.40	7.38	115.4	115.2	115.0	114.8	114.6
**13**	7.35	7.33	7.33	7.25	7.23	123.3	123.0	122.9	122.7	122.4
**8**	7.04	7.02	7.02	6.96	6.94	123.1	122.8	122.6	122.6	122.2
**9**	6.94	6.93	6.92	6.86	6.85	125.7	125.4	125.2	125.0	124.8
**17**	6.89	6.87	6.87	6.82	6.81	125.5	125.3	125.0	124.9	124.7
**16**	6.83	6.82	6.81	6.77	6.76	125.0	124.5	124.3	124.5	124.1
**10**	6.79	6.77	6.76	6.70	6.69	124.1	123.9	123.6	123.4	123.3
**14**	5.45	5.43	5.42	5.36	5.33	52.3	51.9	52.2	51.6	51.6
**11**	4.99	4.97	4.95	4.89	4.89	62.1	61.8	61.6	61.2	61.1
**25**	3.96	3.96	3.96	3.90	3.87	63.7	63.5	63.5	63.0	63.0
**26**	0.90	0.89	0.88	0.84	0.84	14.1	13.9	13.9	13.6	13.3

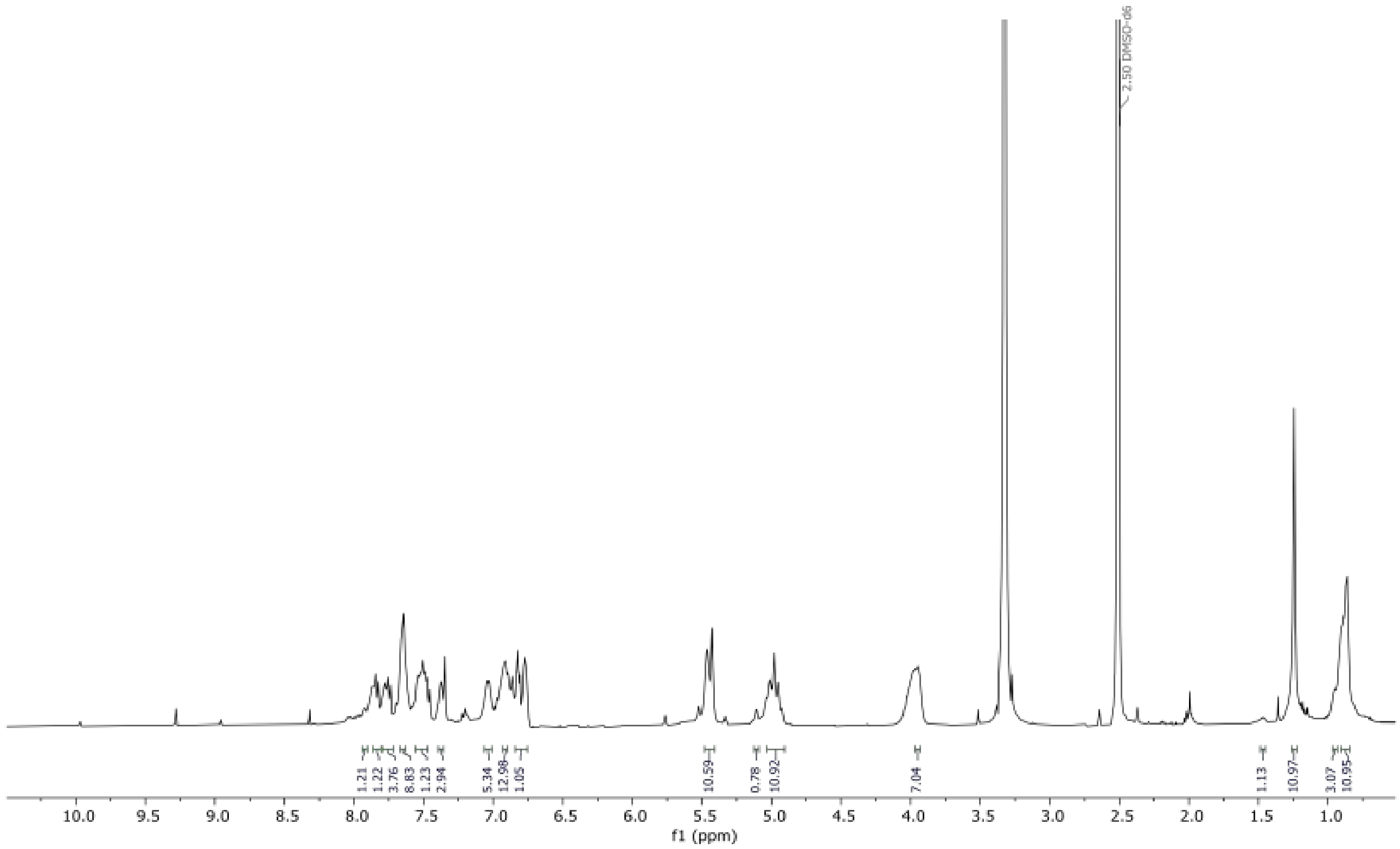

Figure S40: ^{1}H NMR spectrum of compound **10** in DMSO-d_6.

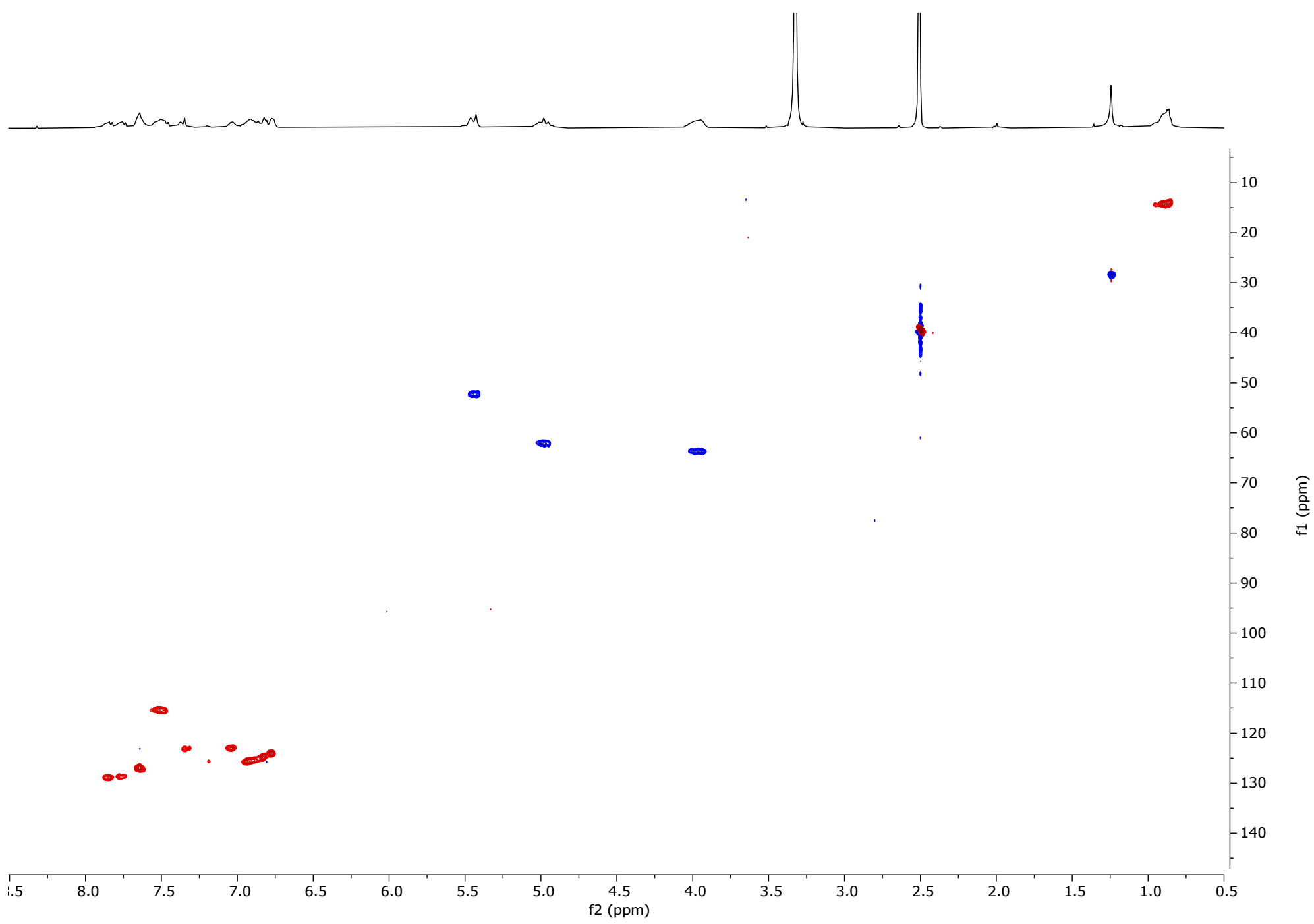

Figure S41: HSQC spectrum of compound **10** in DMSO-d_6.

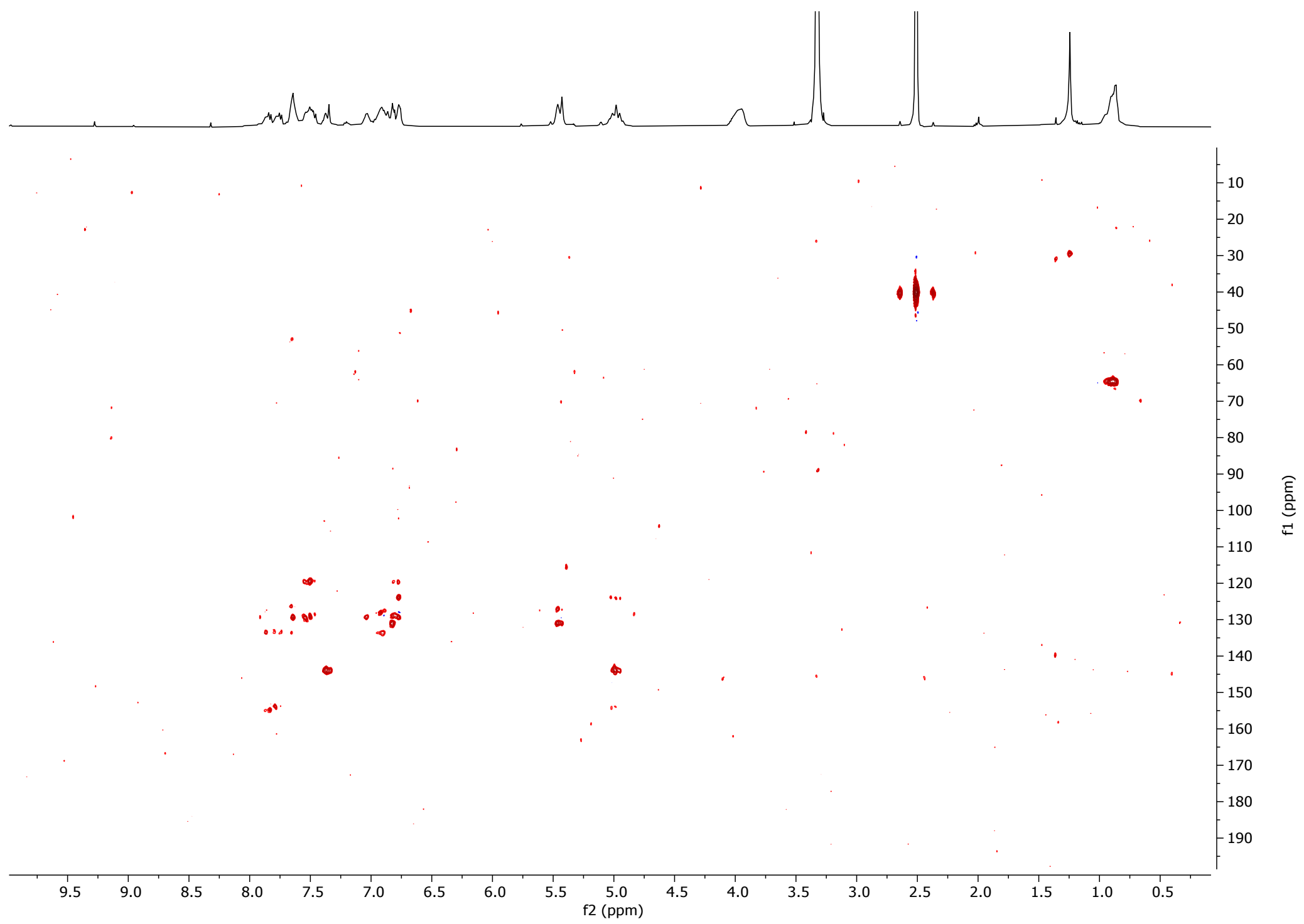

Figure S42: HMBC spectrum of compound **10** in DMSO-d_6.

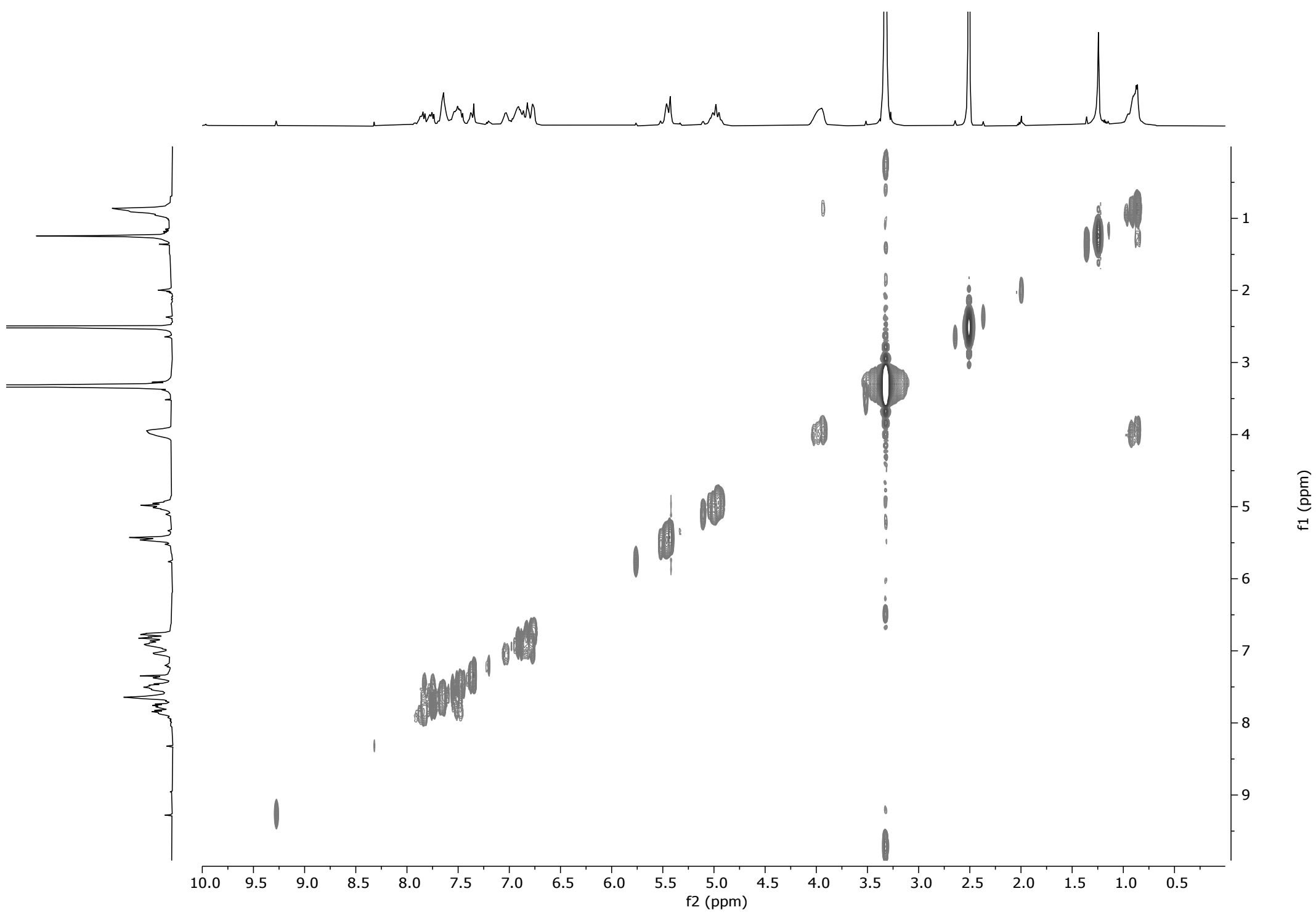

Figure S43: 2D-COSY spectrum of compound **10** in DMSO-d_6.

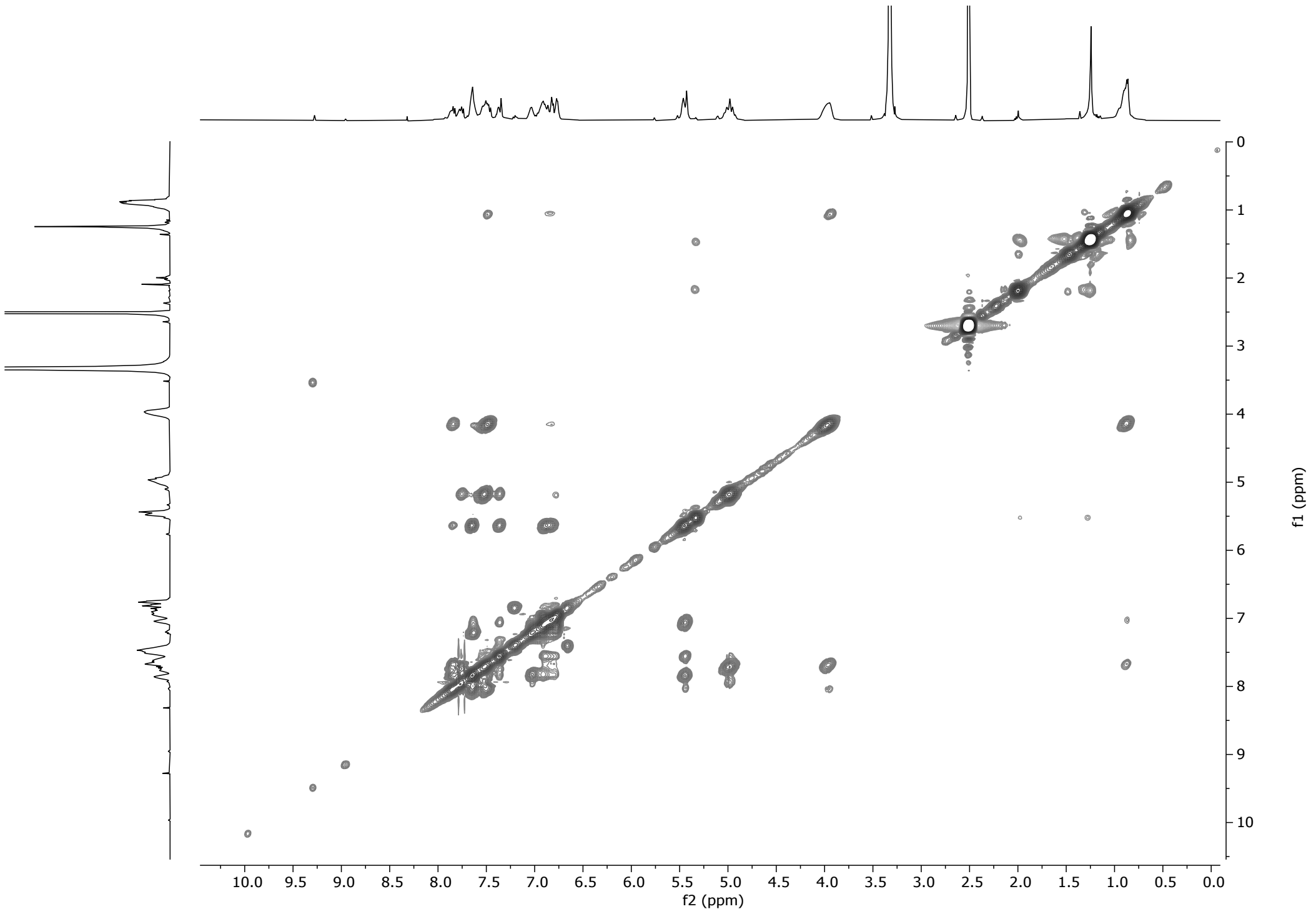

Figure S44: 2D-NOESY spectrum of compound **10** in DMSO-d_6.

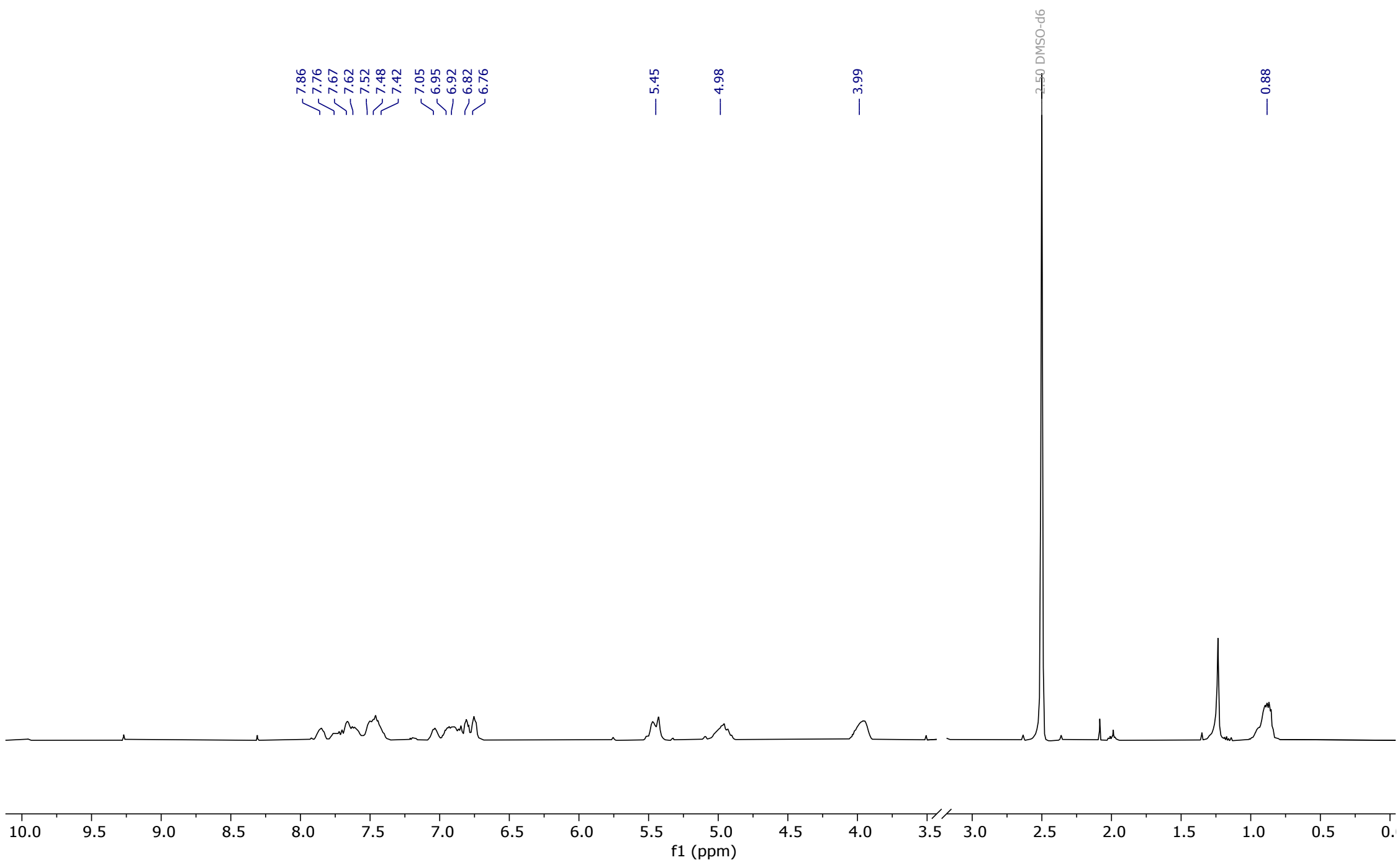

Figure S45: ^{1}H NMR spectrum of compound **10** in DMSO-d_6 after addition of 1 equiv. of $AgNO_3$.

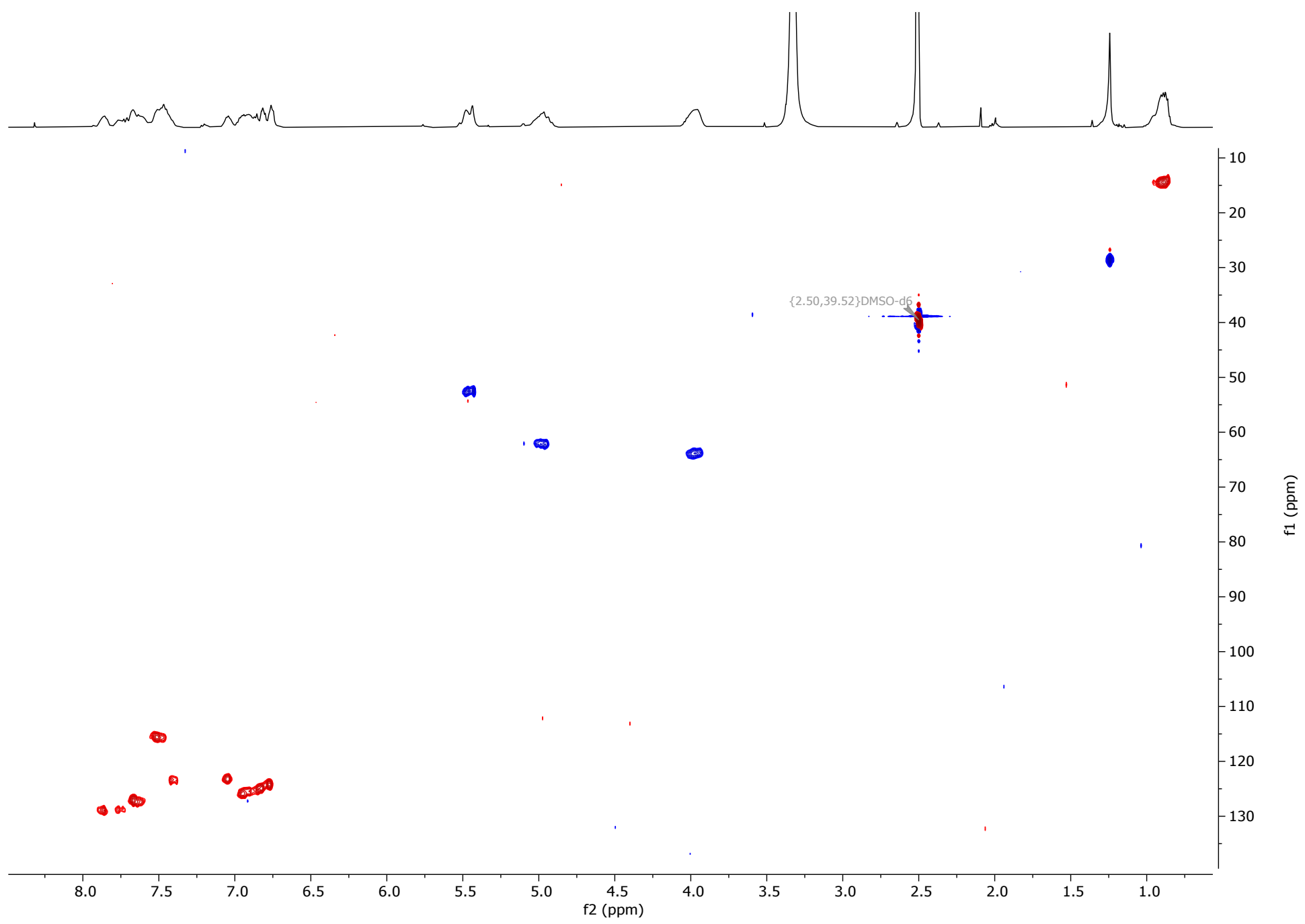

Figure S46: HSQC spectrum of compound **10** in DMSO-d_6 after addition of 1 equiv. of $AgNO_3$.

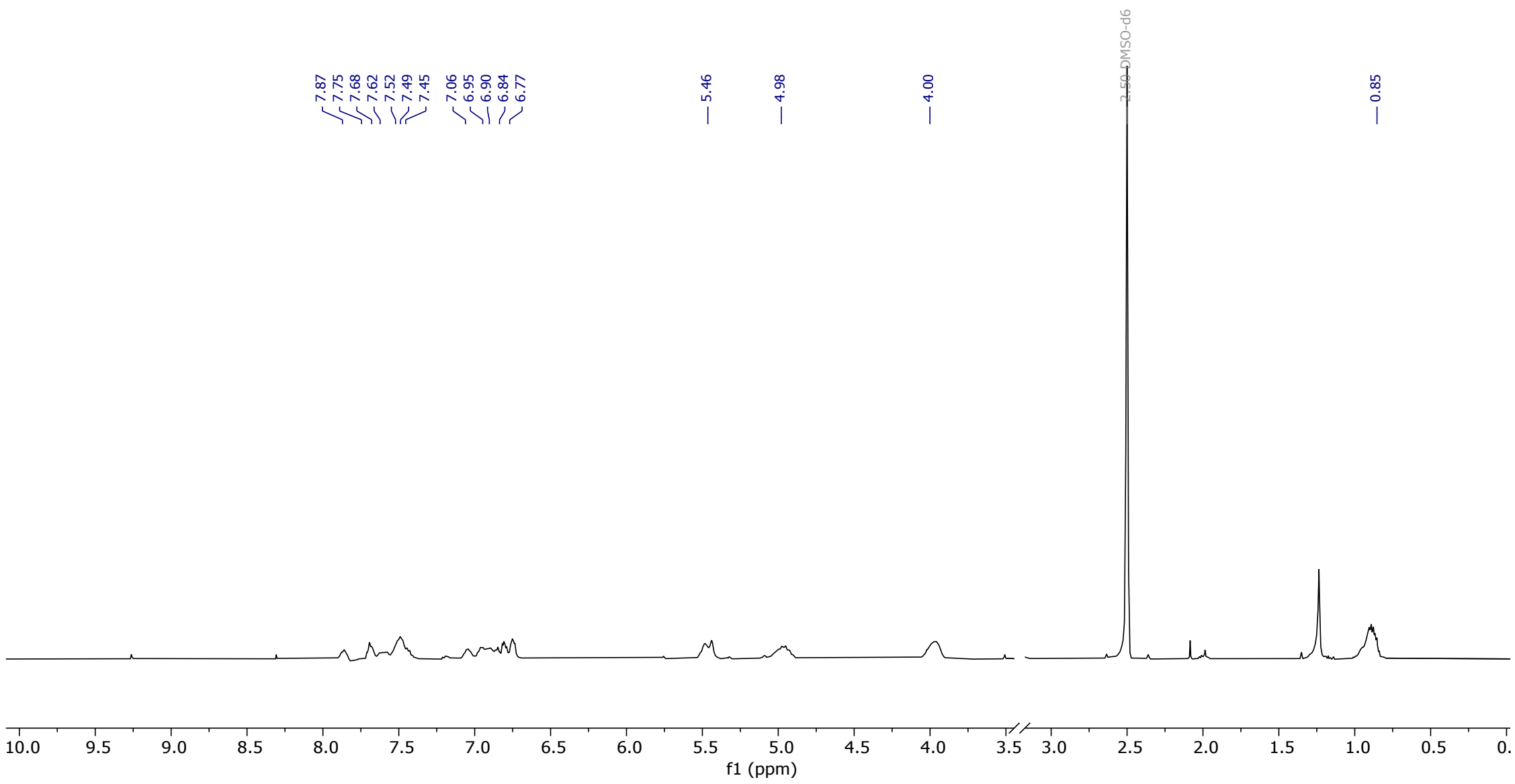

Figure S47: ^{1}H NMR spectrum of compound **10** in DMSO-d_6 after addition of 2 equiv. of $AgNO_3$.

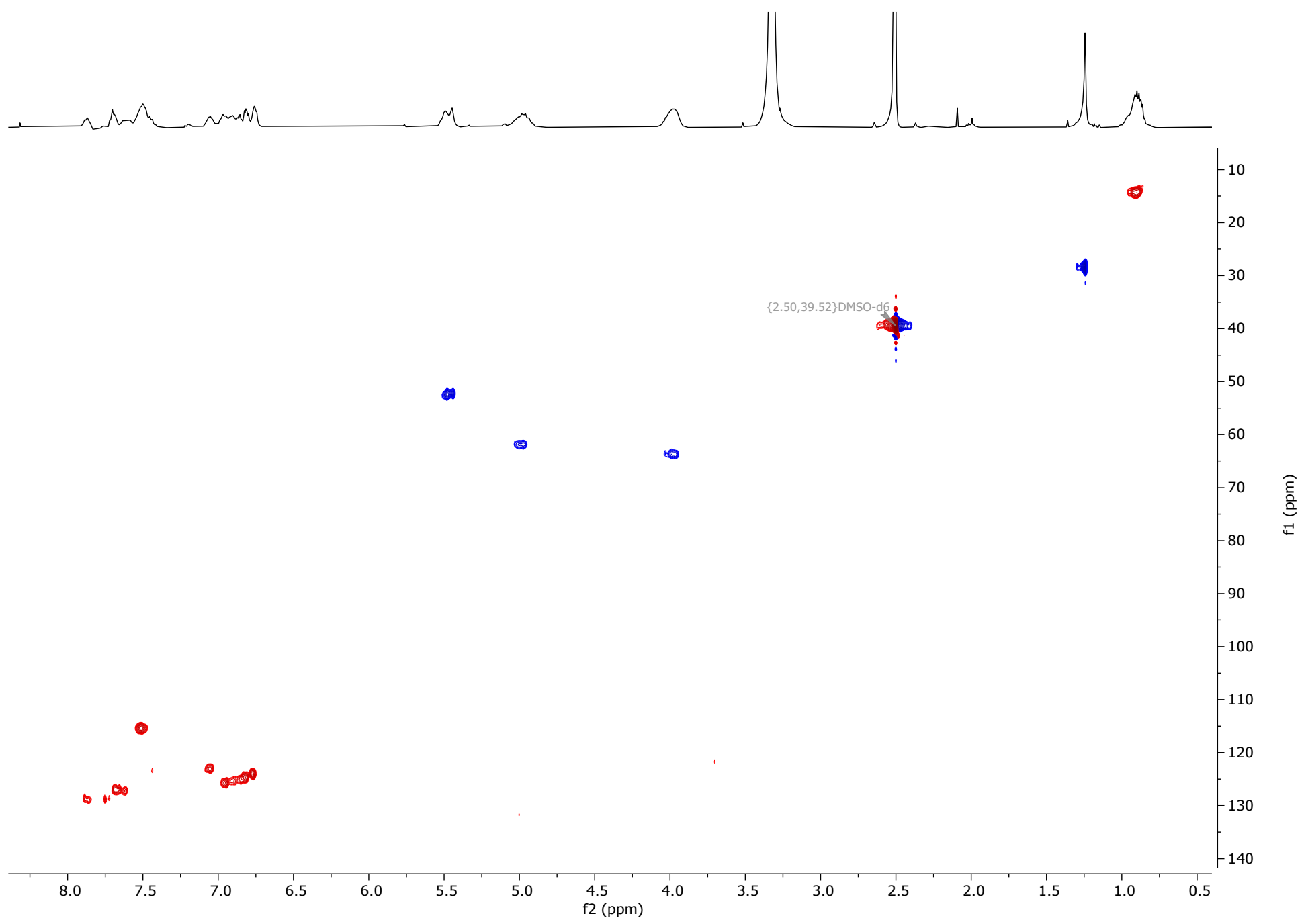

Figure S48: HSQC spectrum of compound **10** in DMSO-d_6 after addition of 2 equiv. of $AgNO_3$.

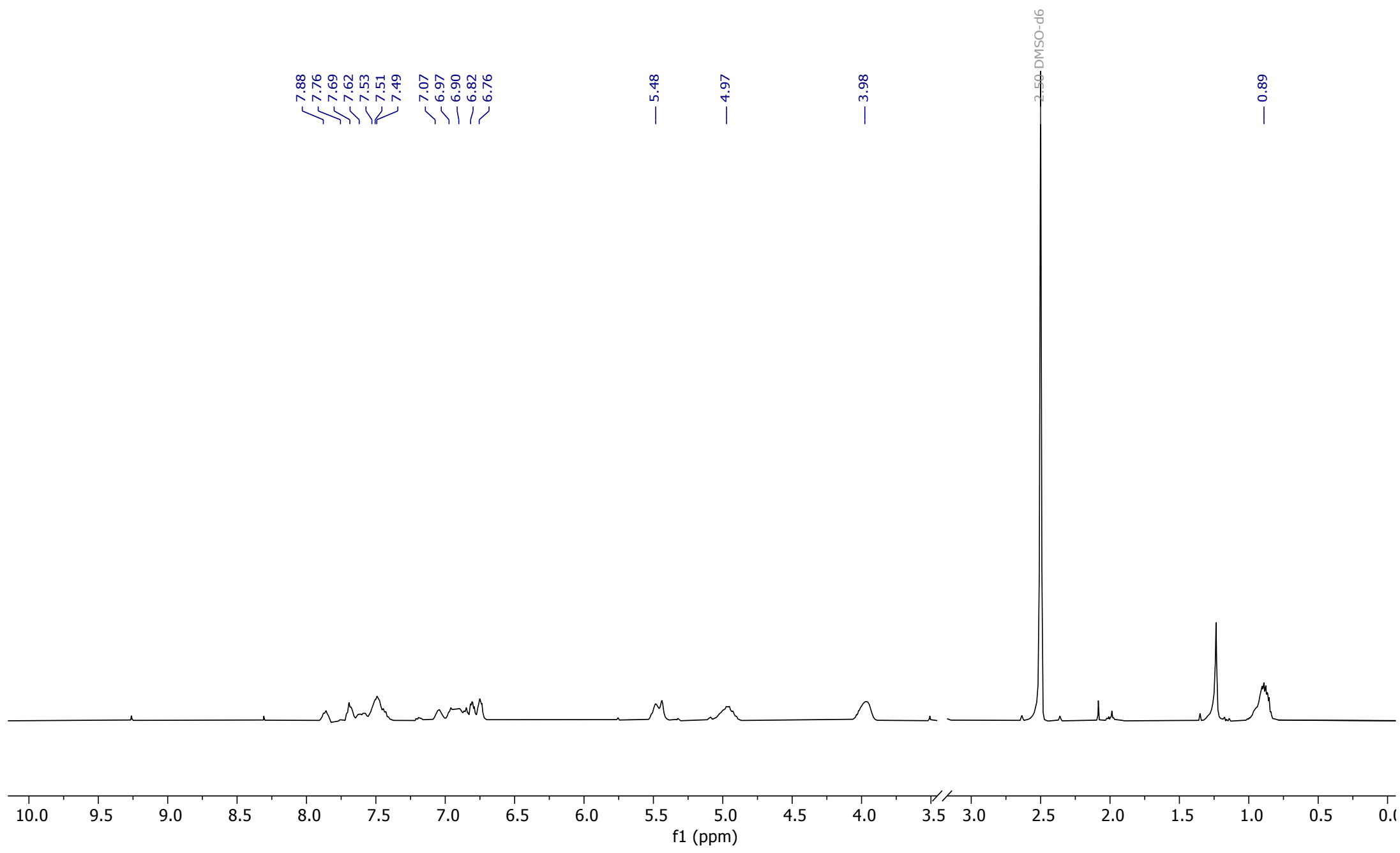

Figure S49: ^{1}H NMR spectrum of compound **10** in DMSO-d_6 after addition of 3 equiv. of $AgNO_3$.

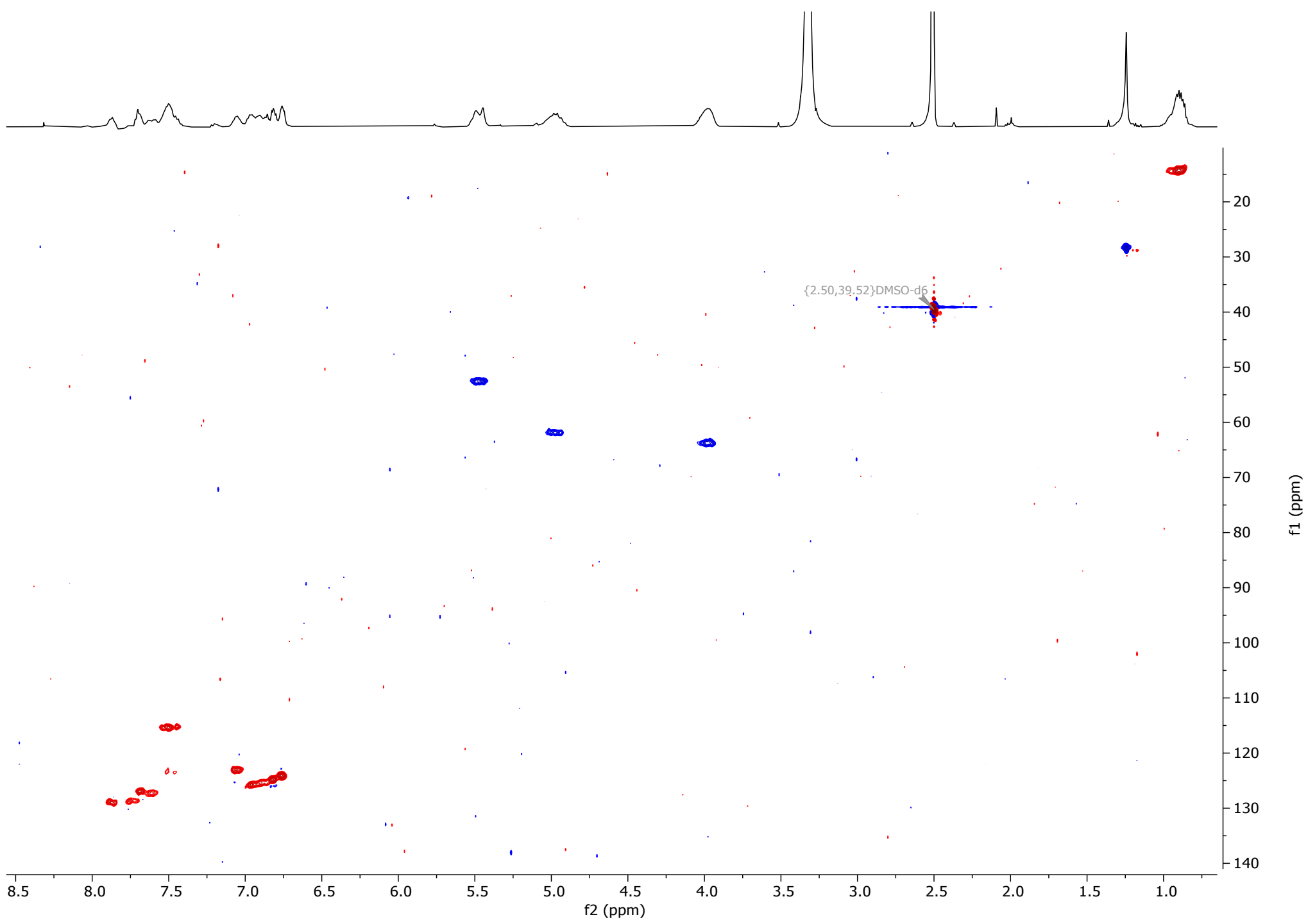

Figure S50: HSQC spectrum of compound **10** in DMSO-d_6 after addition of 3 equiv. of $AgNO_3$.

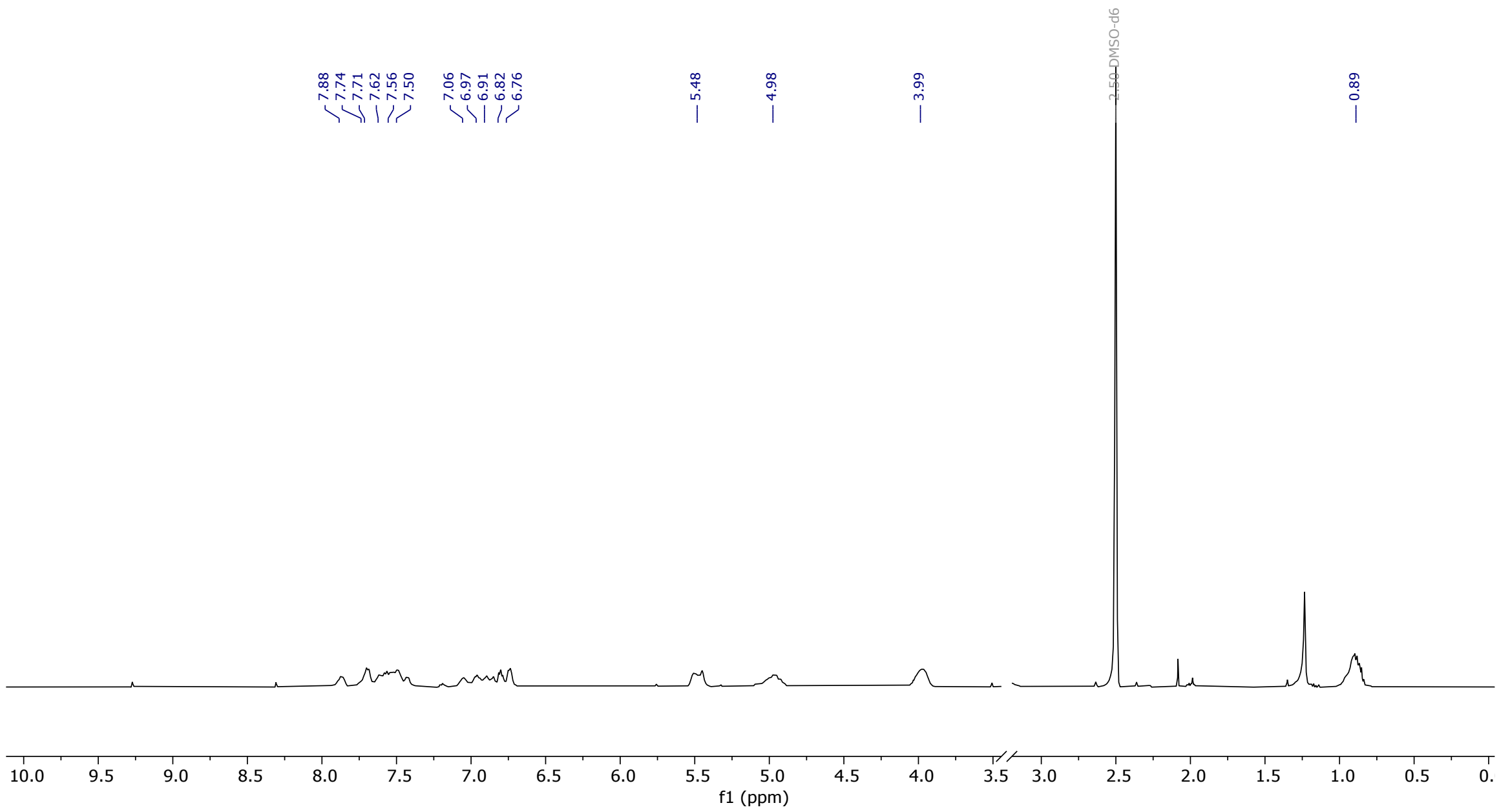

Figure S51: ^{1}H NMR spectrum of compound **10** in DMSO-d_6 after addition of 4 equiv. of $AgNO_3$.

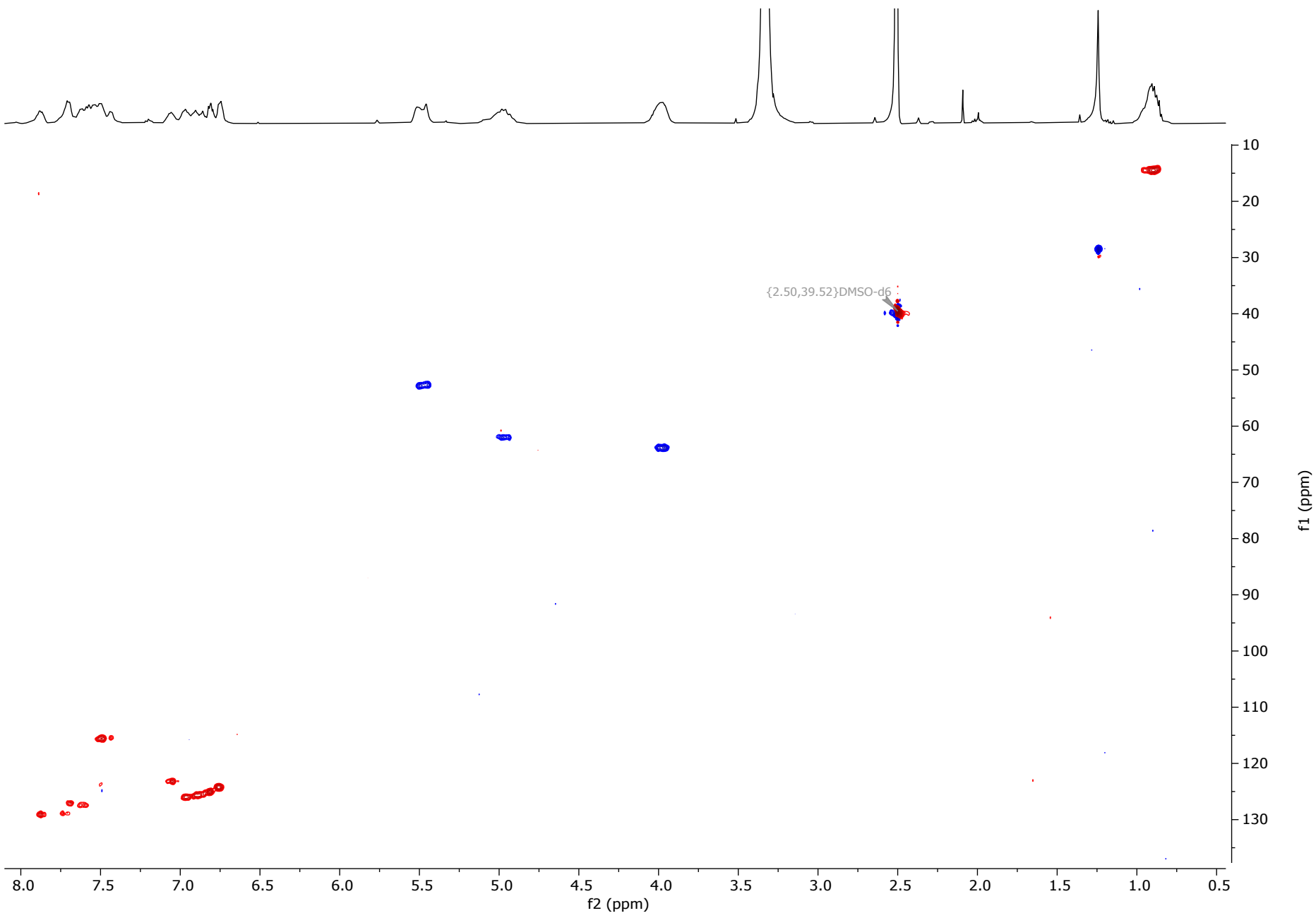

Figure S52: HSQC spectrum of compound **10** in DMSO-d_6 after addition of 4 equiv. of $AgNO_3$.

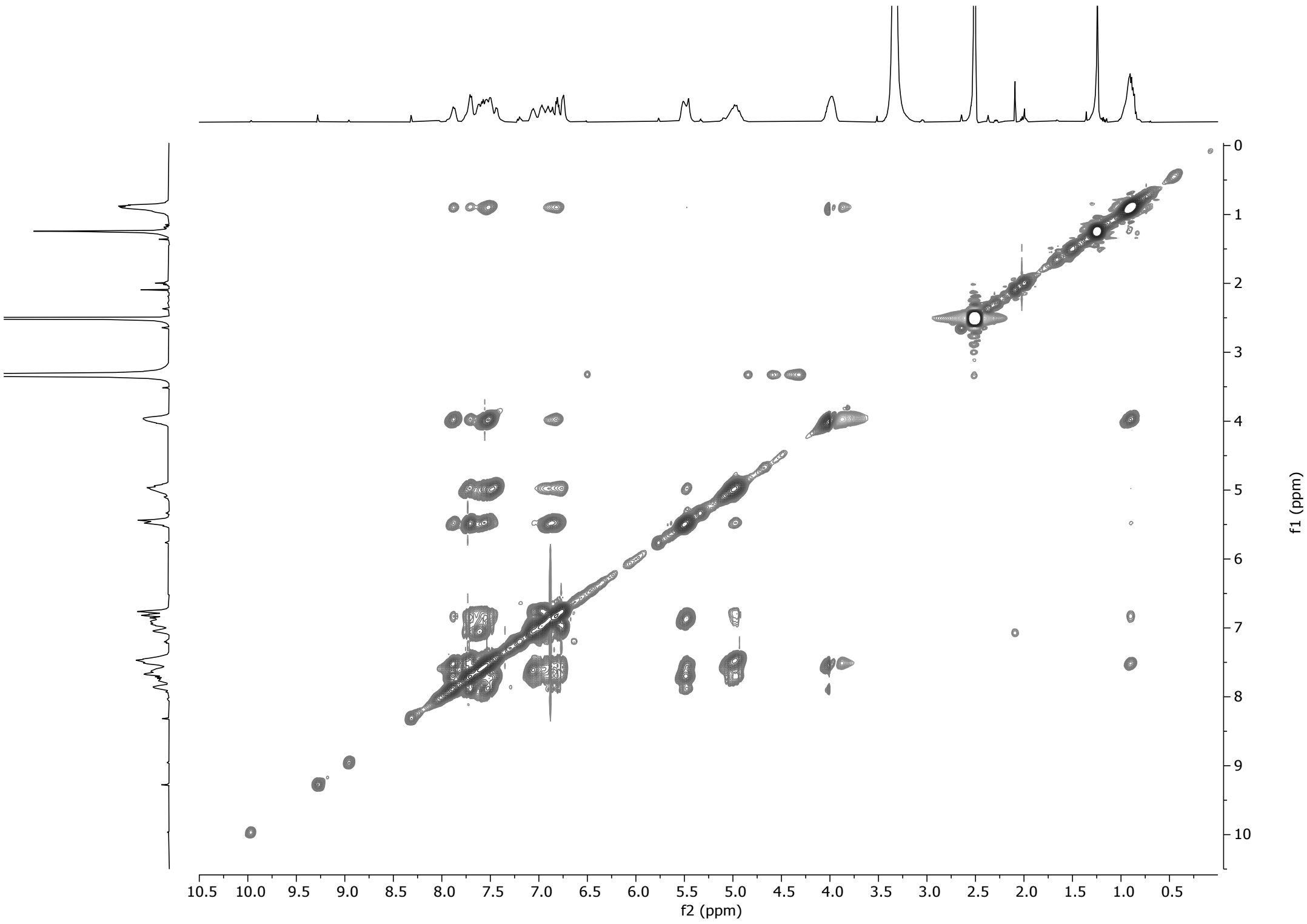

Figure S53: 2D-NOESY spectrum of compound **10** in DMSO-d_6 after addition of 4 equiv. of $AgNO_3$.

Table S3: ^{1}H and ^{13}C chemical shifts of compound **10** in DMSO-d_6 in absence and in presence of 1, 2, 3, 4 equiv. of $AgNO_3$.

**^{1}H chemical shifth (ppm)**						**^{13}C chemical shifth (ppm)**				
**Peaks**	**10**	**10:** $AgNO_3$ **1:1**	**10:** $AgNO_3$ **1:2**	**10:** $AgNO_3$ **1:3**	**10:** $AgNO_3$ **1:4**	**10**	**10:** $AgNO_3$ **1:1**	**10:** $AgNO_3$ **1:2**	**10:** $AgNO_3$ **1:3**	**10:** $AgNO_3$ **1:4**
**21**	7.85	7.86	7.87	7.88	7.89	129.0	128.8	129.0	128.8	128.9
**6**	7.76	7.76	7.75	7.76	7.74	128.7	128.7	128.8	128.6	128.7
**7**	7.66	7.67	7.68	7.69	7.71	126.9	127.0	127.0	126.9	127.0
**20**	7.63	7.62	7.62	7.62	7.62	127.2	127.3	127.4	127.1	127.3
**5**	7.53	7.52	7.52	7.53	7.55	115.4	115.4	115.4	115.3	115.4
**22**	7.49	7.48	7.49	7.50	7.50	115.2	115.3	115.4	115.3	115.3
**13**	7.33	7.42	7.45	7.49	7.50	123.1	123.5	123.6	123.5	123.7
**8**	7.04	7.05	7.06	7.07	7.06	123.0	123.0	123.1	123.0	123.0
**9**	6.94	6.95	6.95	6.97	6.97	125.8	125.7	125.8	125.7	125.7
**17**	6.89	6.92	6.90	6.90	6.91	125.5	125.4	125.4	125.3	125.4
**16**	6.83	6.82	6.83	6.82	6.82	124.9	124.7	124.7	124.8	124.8
**10**	6.78	6.77	6.77	6.76	6.77	124.1	124.1	124.2	124.1	124.1
**14**	5.44	5.45	5.47	5.48	5.48	52.4	52.2	52.4	52.3	52.4
**11**	4.97	4.98	4.98	4.97	4.98	62.0	62.0	61.9	61.8	61.8
**25**	3.98	3.99	4.00	3.98	3.99	63.8	63.8	63.6	63.5	63.8
**26**	0.88	0.89	0.85	0.89	0.89	14.4	14.4	14.2	14.3	14.5

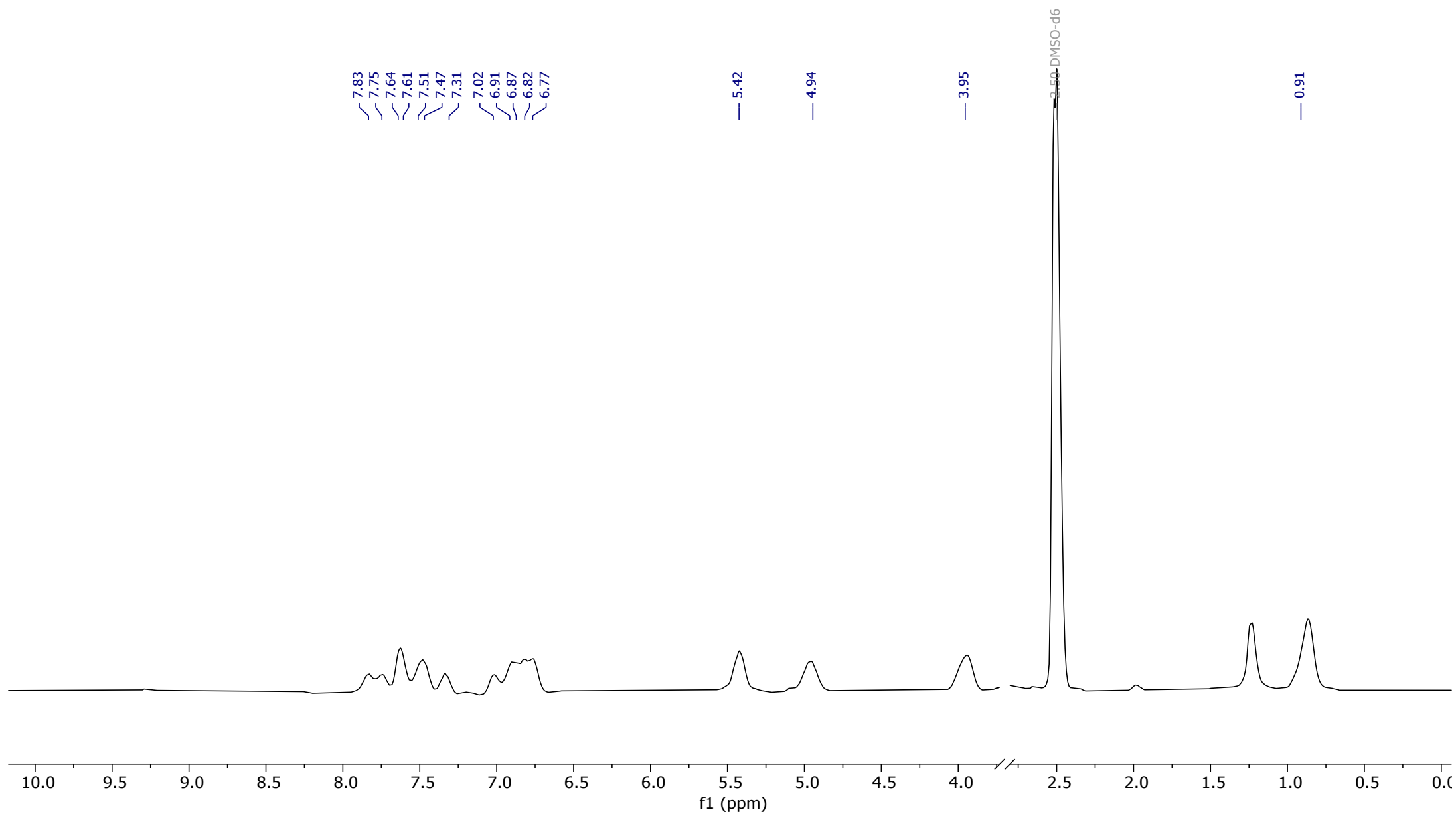

Figure S54: ^{1}H NMR spectrum of compound **10** in DMSO-d_6 after addition of 1 equiv. of $CoCl_2$.

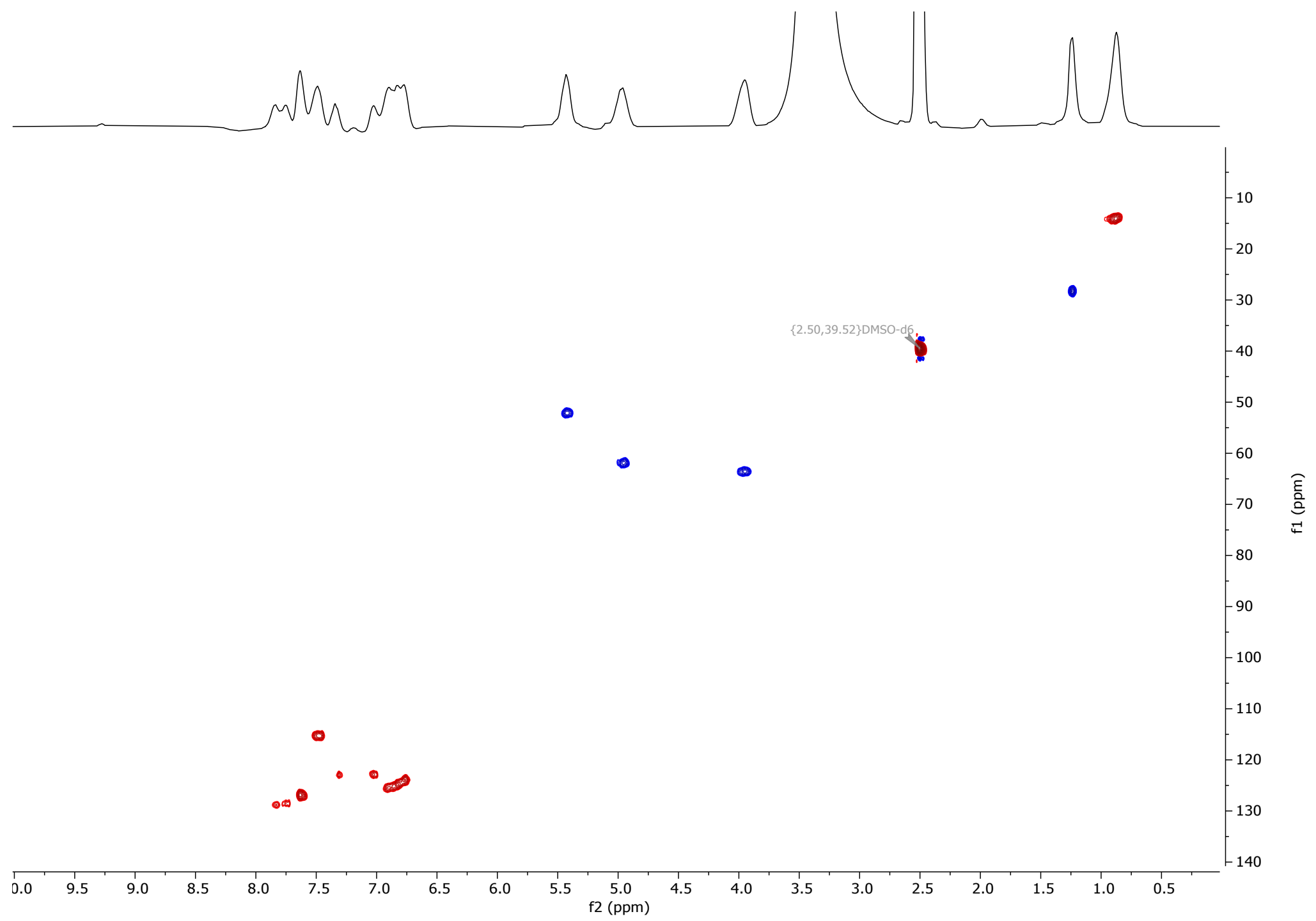

Figure S55: HSQC spectrum of compound **10** in DMSO-d_6 after addition of 1 equiv. of $CoCl_2$.

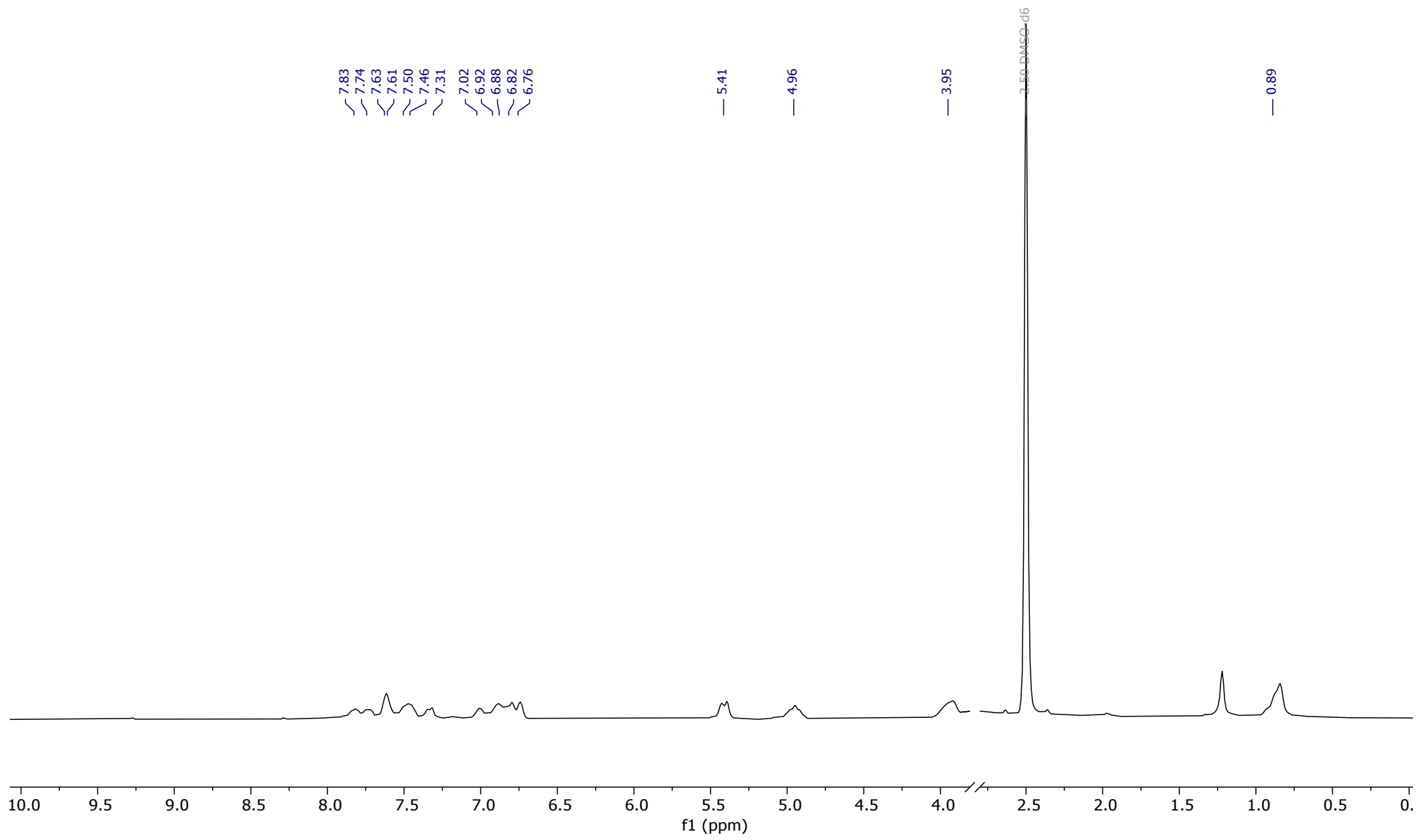

Figure S56: ^{1}H NMR spectrum of compound **10** in DMSO-d_6 after addition of 2 equiv. of $CoCl_2$.

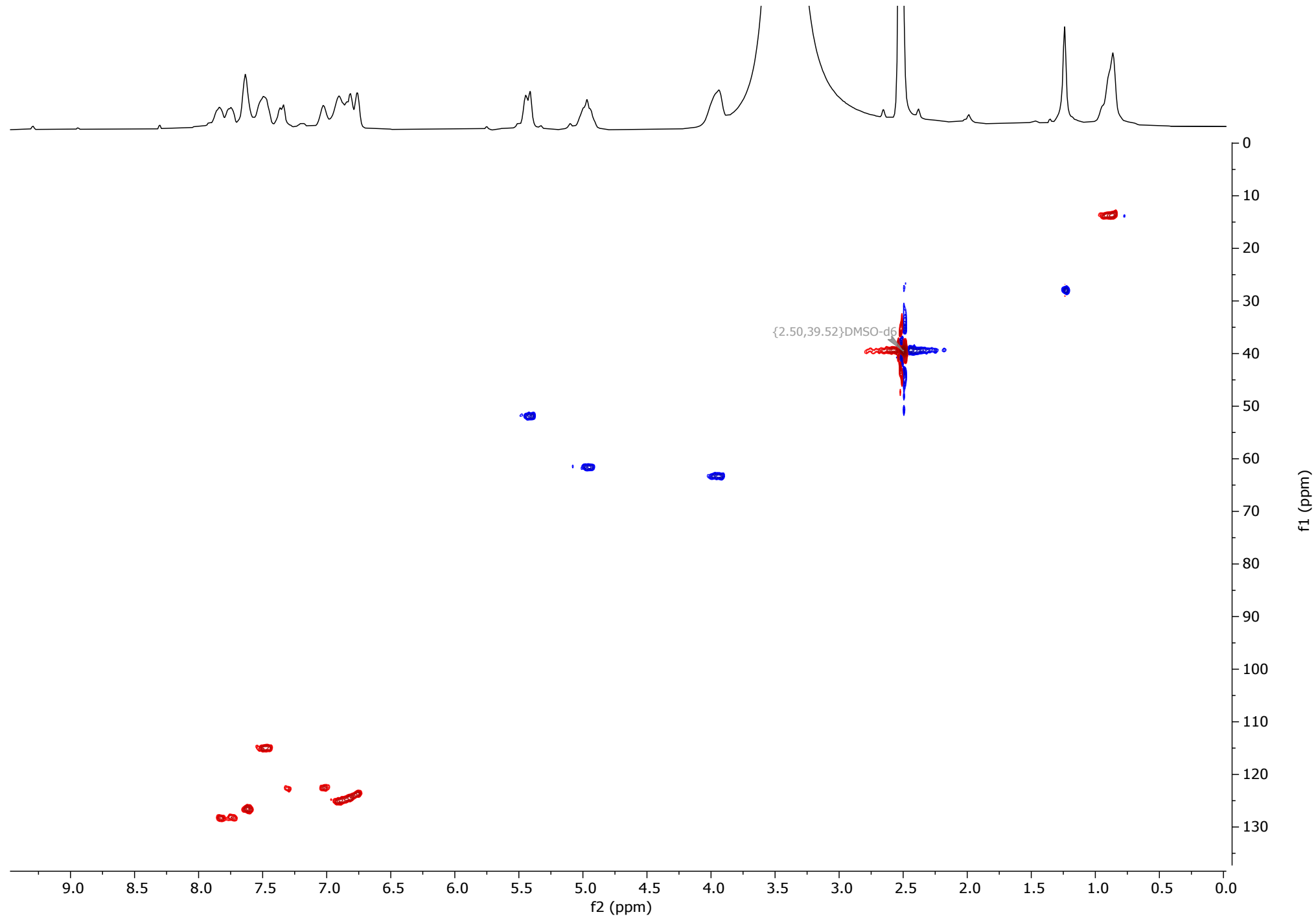

Figure S57: HSQC spectrum of compound **10** in DMSO-d_6 after addition of 2 equiv. of $CoCl_2$.

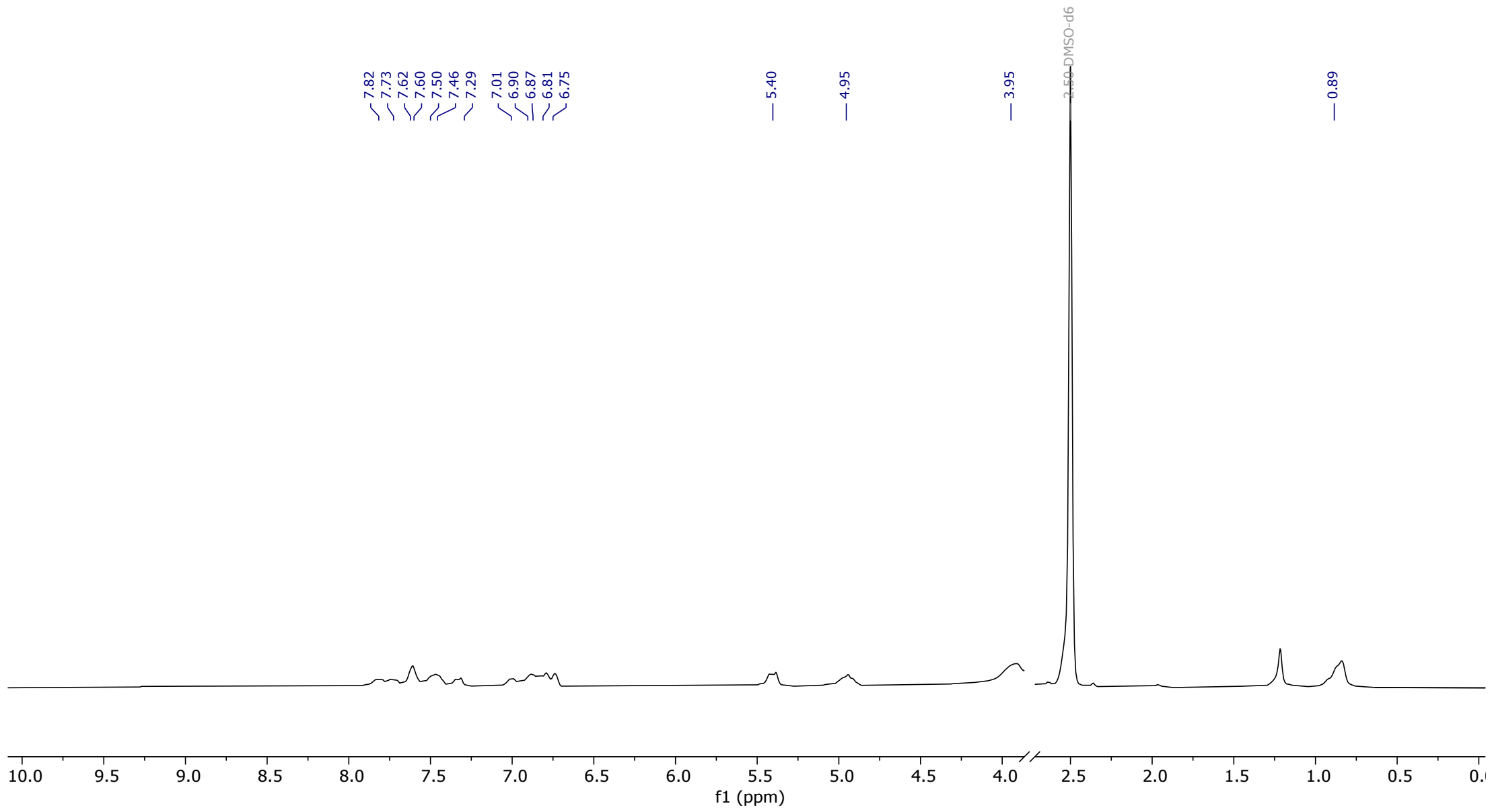

Figure S58: ^{1}H NMR spectrum of compound **10** in DMSO-d_6 after addition of 3 equiv. of $CoCl_2$.

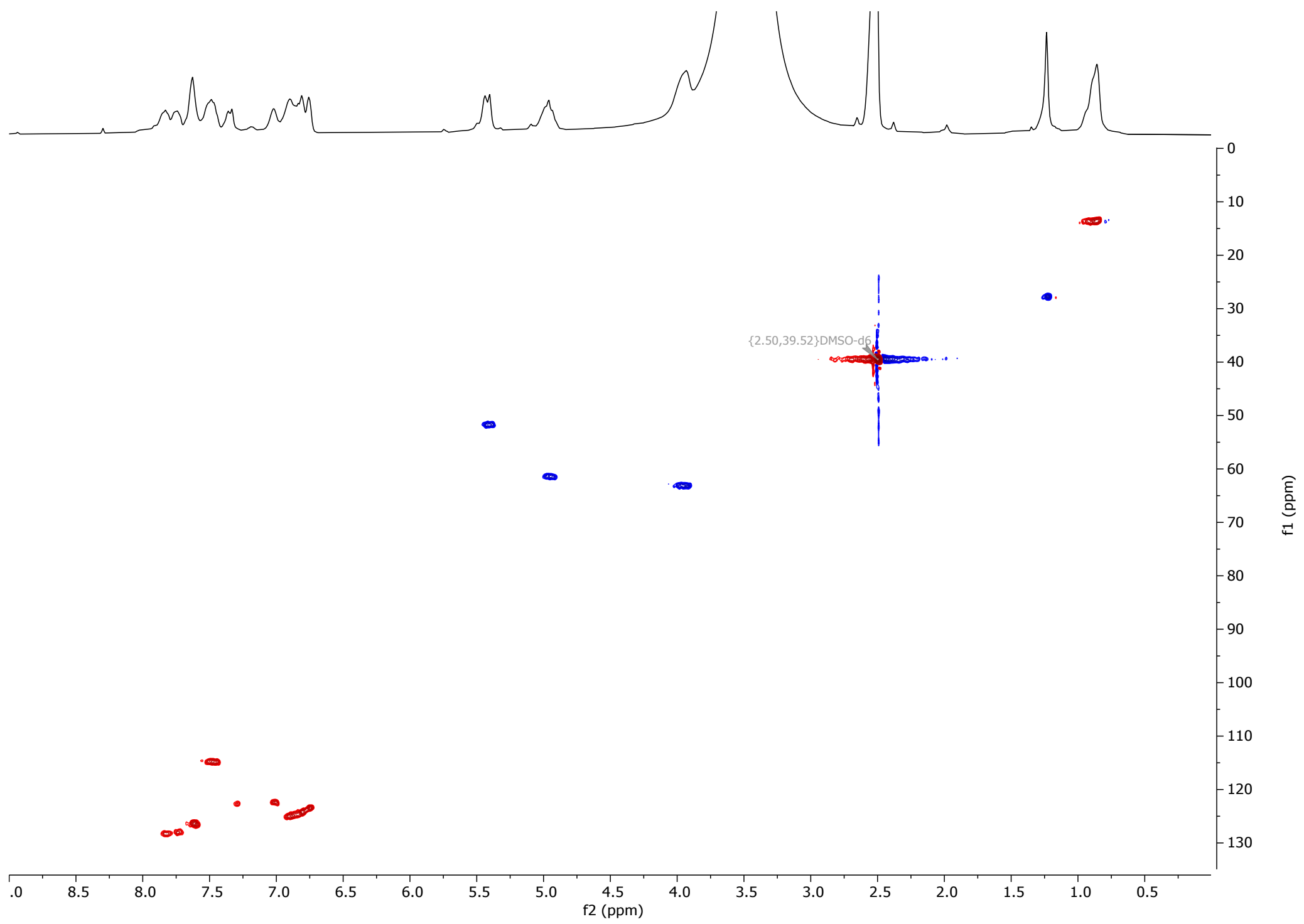

Figure S59: HSQC spectrum of compound **10** in DMSO-d_6 after addition of 3 equiv. of $CoCl_2$.

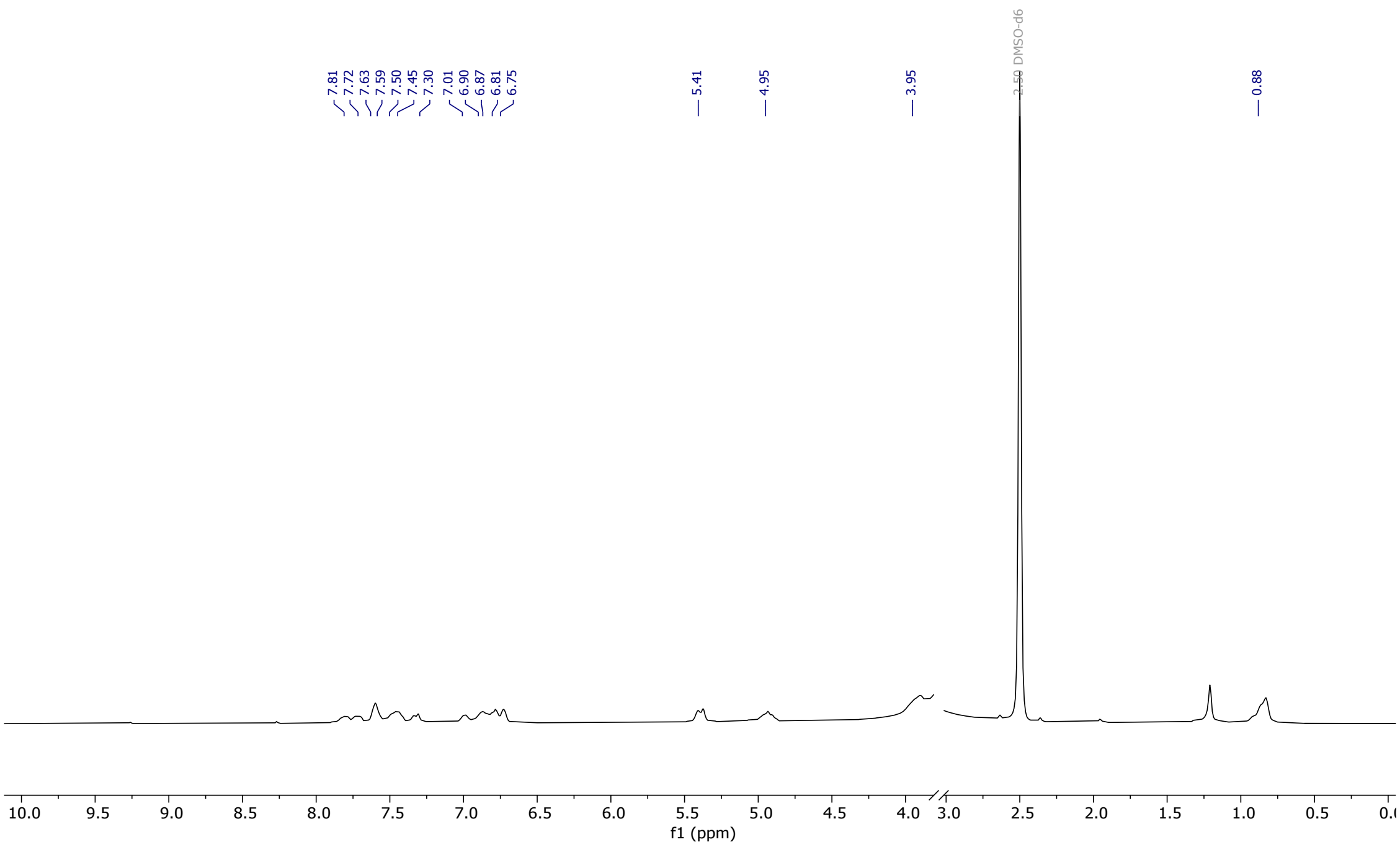

Figure S60: ^1H NMR spectrum of compound **10** in DMSO-d_6 after addition of 4 equiv. of $CoCl_2$.

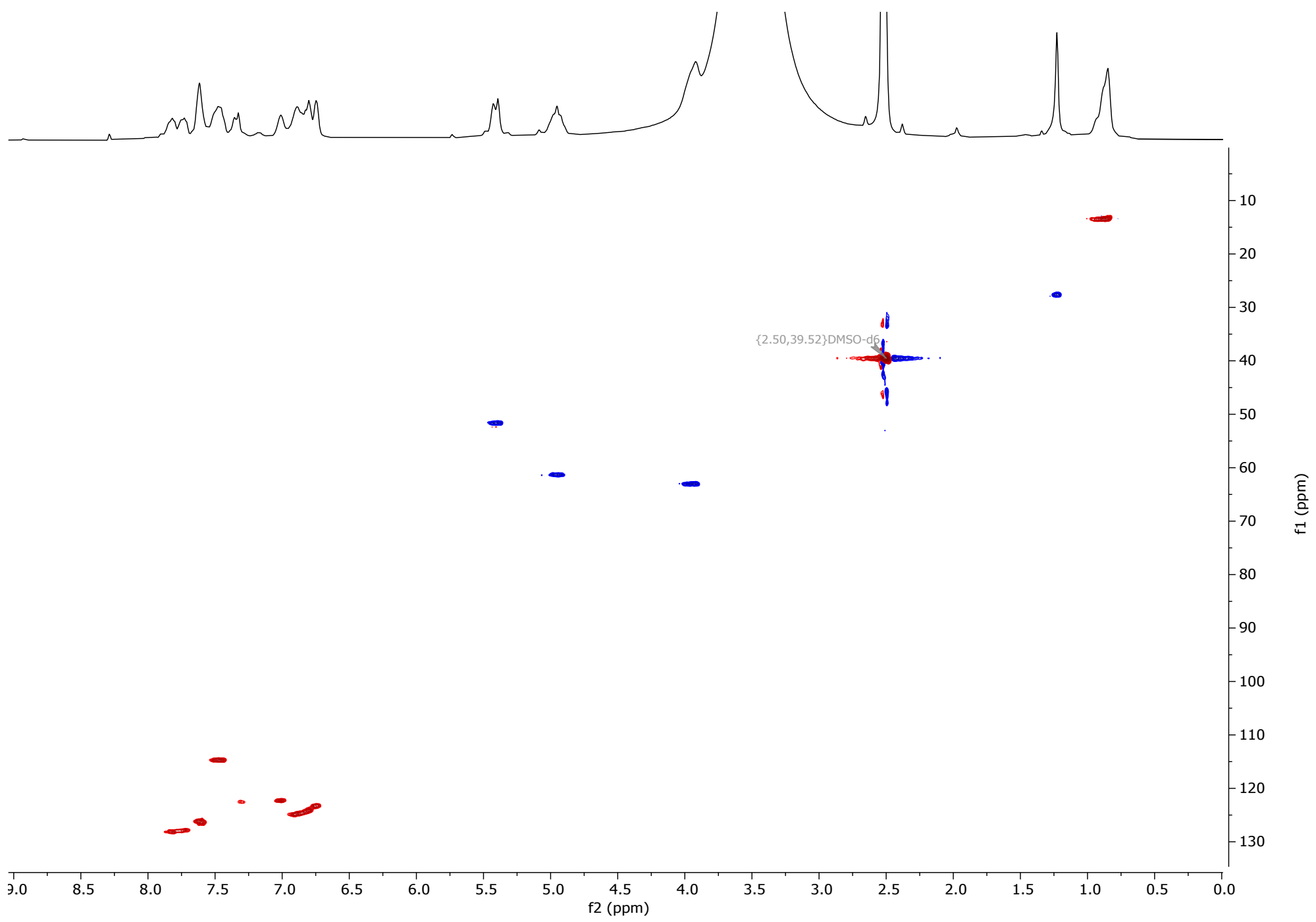

Figure S61: HSQC spectrum of compound **10** in DMSO-d_6 after addition of 4 equiv. of $CoCl_2$.

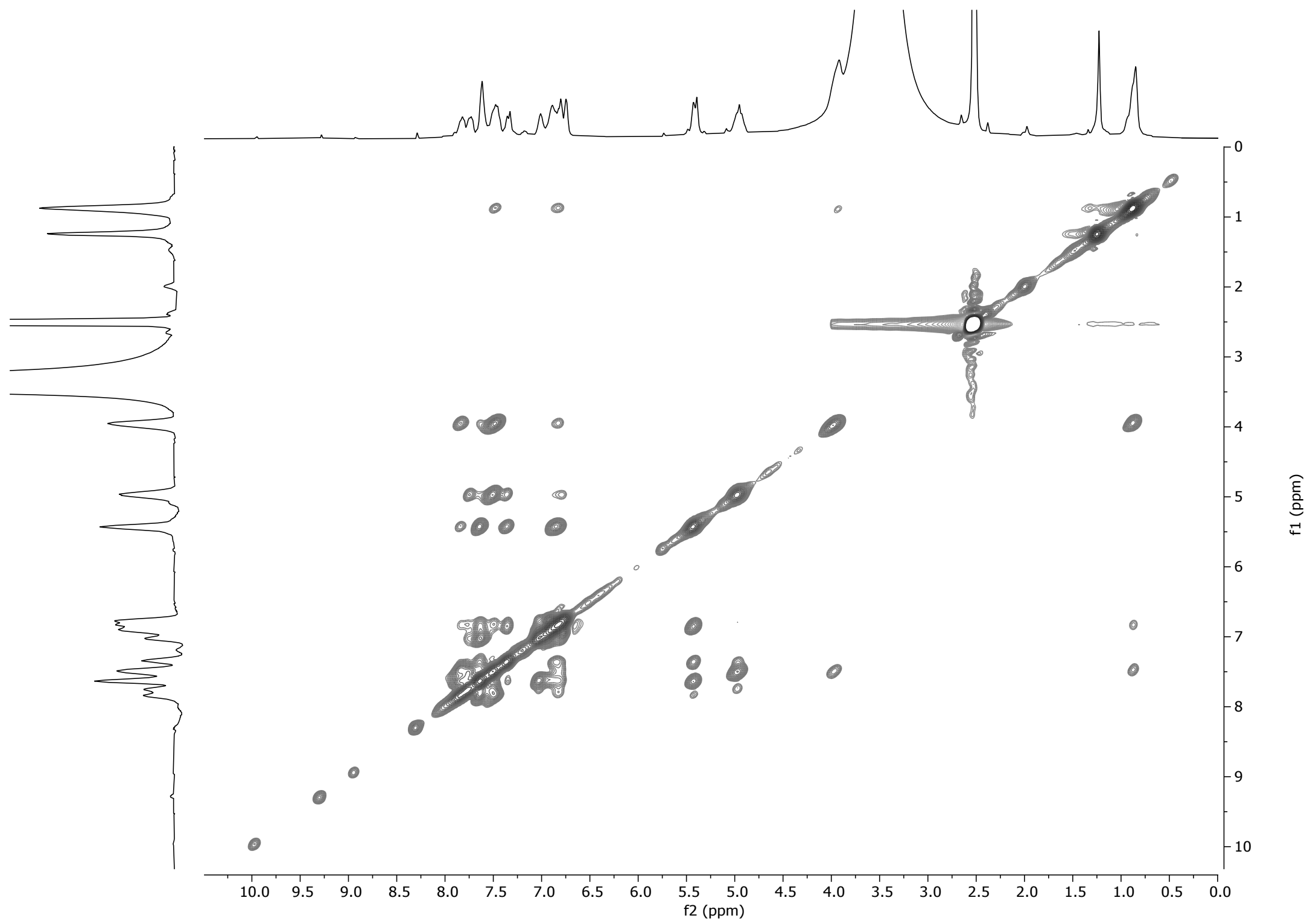

Figure S62: 2D-NOESY spectrum of compound **10** in DMSO-d_6 after addition of 4 equiv. of $CoCl_2$

Table S4: ^{1}H and ^{13}C chemical shifts of compound **10** in DMSO-d_6 in absence and in presence of 1, 2, 3, 4 equiv. of $CoCl_2$.

^{1}H chemical shift (ppm)						^{13}C chemical shift (ppm)				
**Peaks**	**10**	**10: $CoCl_2$ 1:1**	**10: $CoCl_2$ 1:2**	**10: $CoCl_2$ 1:3**	**10: $CoCl_2$ 1:4**	**10**	**10: $CoCl_2$ 1:1**	**10: $CoCl_2$ 1:2**	**10: $CoCl_2$ 1:3**	**10: $CoCl_2$ 1:4**
**21**	7.85	7.83	7.83	7.82	7.81	129.0	128.7	128.4	128.3	128.1
**6**	7.76	7.75	7.74	7.73	7.72	128.7	128.4	128.2	128.0	127.8
**7**	7.66	7.64	7.63	7.62	7.63	126.9	126.7	126.6	126.3	126.0
**20**	7.63	7.61	7.61	7.60	7.59	127.2	126.9	126.6	126.5	126.4
**5**	7.53	7.51	7.50	7.50	7.50	115.4	115.3	115.1	114.8	114.6
**22**	7.49	7.47	7.46	7.46	7.45	115.2	115.3	115.1	114.9	114.6
**13**	7.33	7.30	7.30	7.29	7.30	123.1	123.1	122.9	122.7	122.4
**8**	7.04	7.02	7.02	7.01	7.01	123.0	122.8	122.7	122.5	122.2
**9**	6.94	6.91	6.92	6.90	6.90	125.8	125.4	125.2	125.1	124.8
**17**	6.89	6.87	6.88	6.87	6.87	125.5	125.2	124.9	124.9	124.8
**16**	6.83	6.82	6.82	6.81	6.80	124.9	124.8	124.5	124.4	124.1
**10**	6.78	6.77	6.76	6.75	6.75	124.1	123.9	123.7	123.5	123.4
**14**	5.44	5.42	5.41	5.40	5.40	52.4	52.0	51.9	51.8	51.5
**11**	4.97	4.95	4.96	4.95	4.95	62.0	61.8	61.8	61.5	61.2
**25**	3.98	3.96	3.95	3.95	3.95	63.8	63.6	63.4	63.0	63.1
**26**	0.91	0.91	0.89	0.89	0.88	14.7	14.1	13.9	13.3	13.3

- **UV/Vis and Fluorescence studies concerning the macrocycle 5**

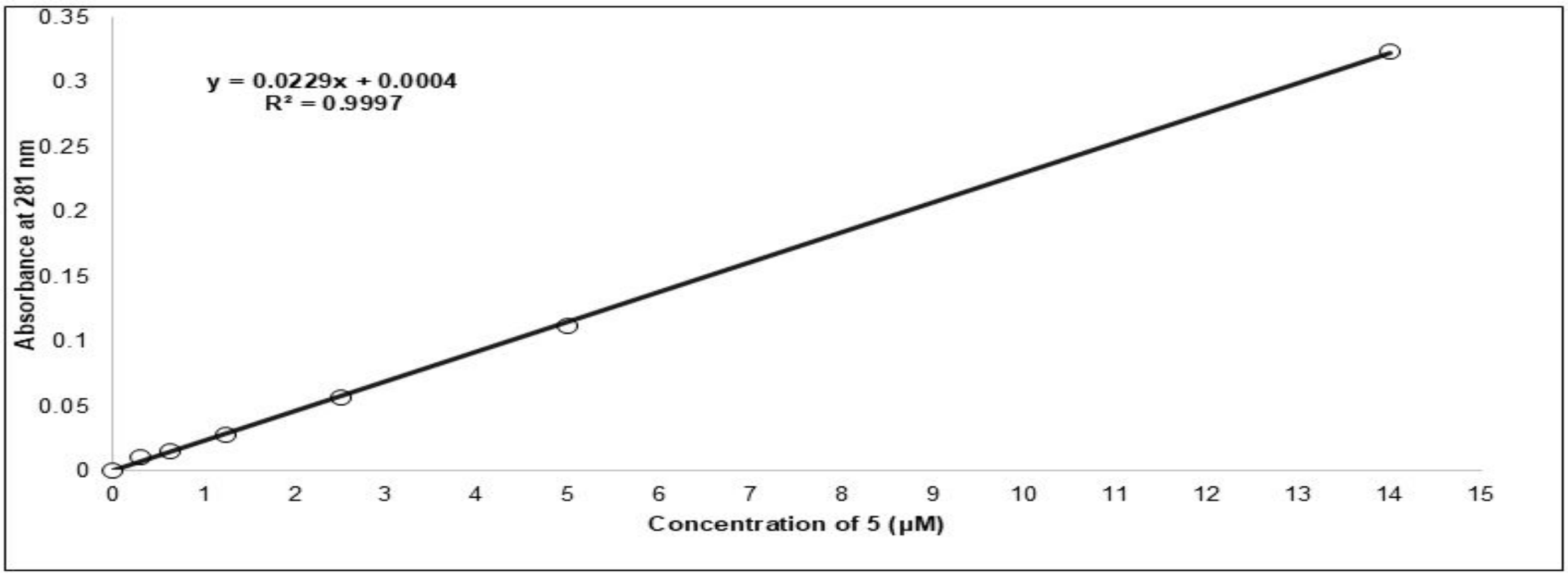

Figure S63. Calibration plot of **5** (concentration range 0-14 μM) at 281 nm using UV/Vis spectroscopy.

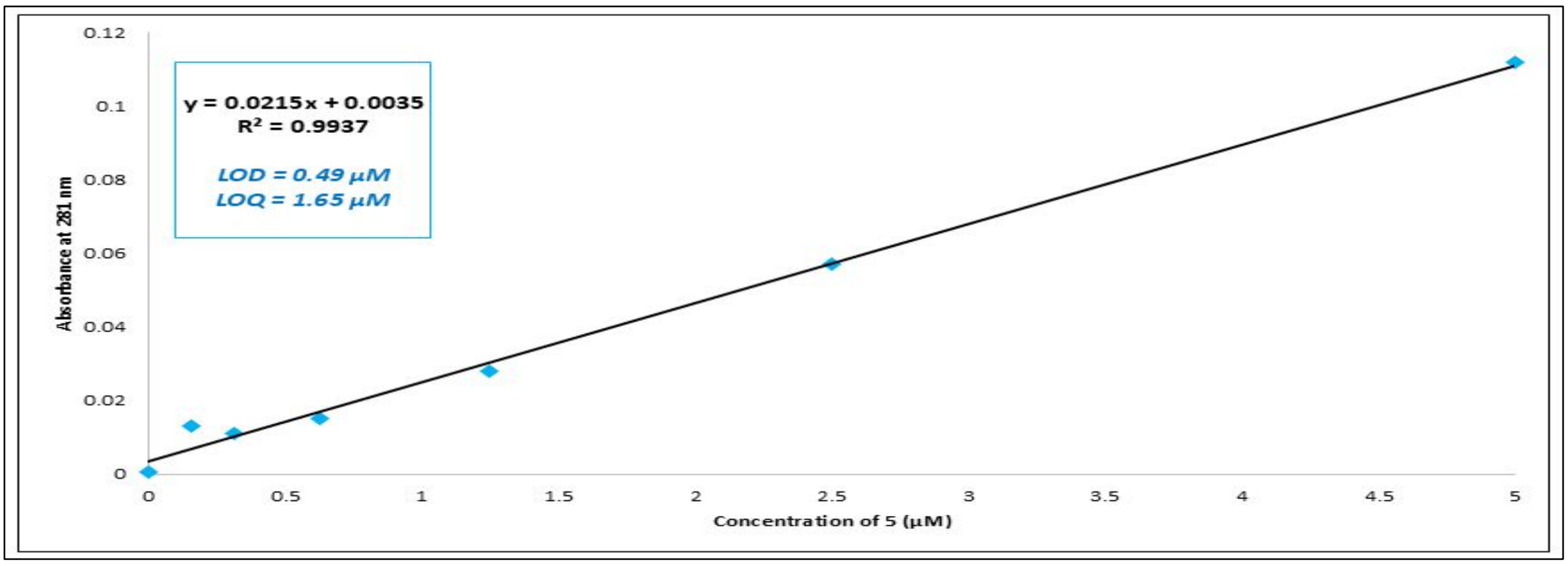

Figure S64. Determination of LOD and LOQ of **5** (concentration range 0-5 μM) at 281 nm using UV/Vis spectroscopy.

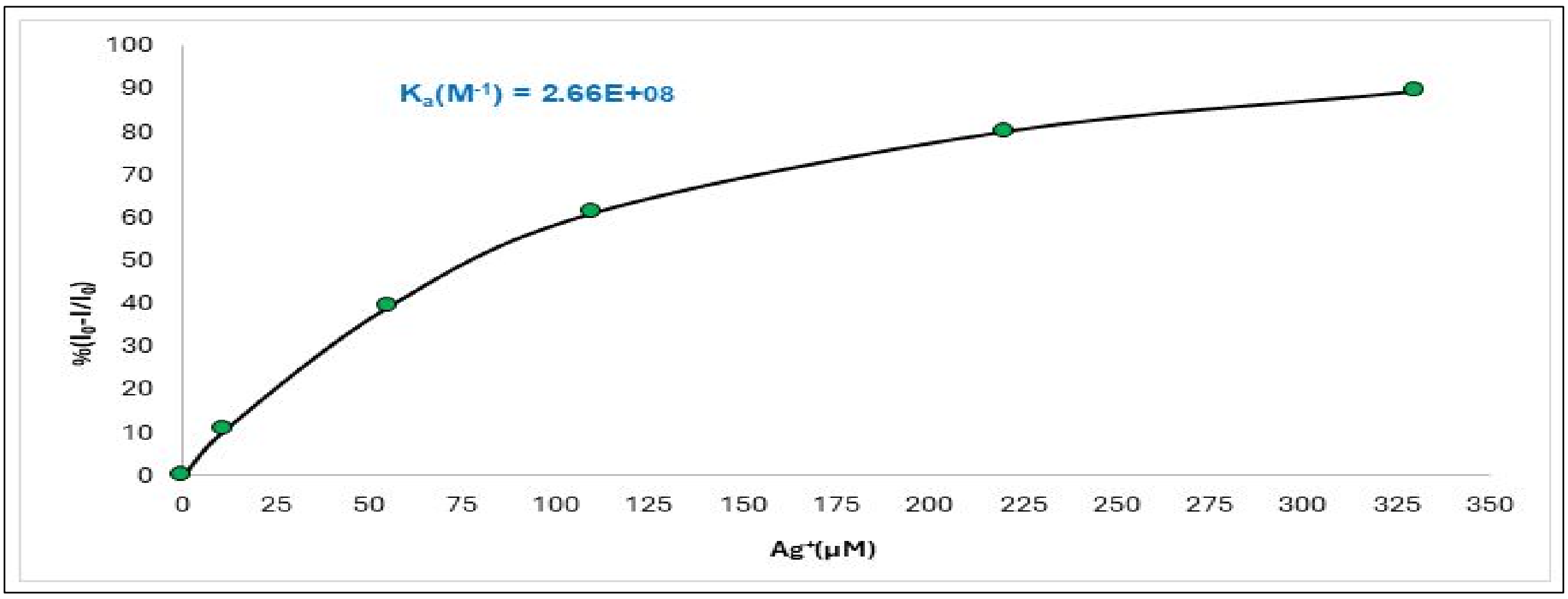

Figure S65. Determination of the Ka of **5** by fluorescence spectroscopy according to the calculation method according to the literature[4] (the concentration of **5** is 11 μM in MeOH/DMSO, *v/v*, 99/1 plus Ag^+ at 0, 1, 5, 10, 20 and 30 equivalents).

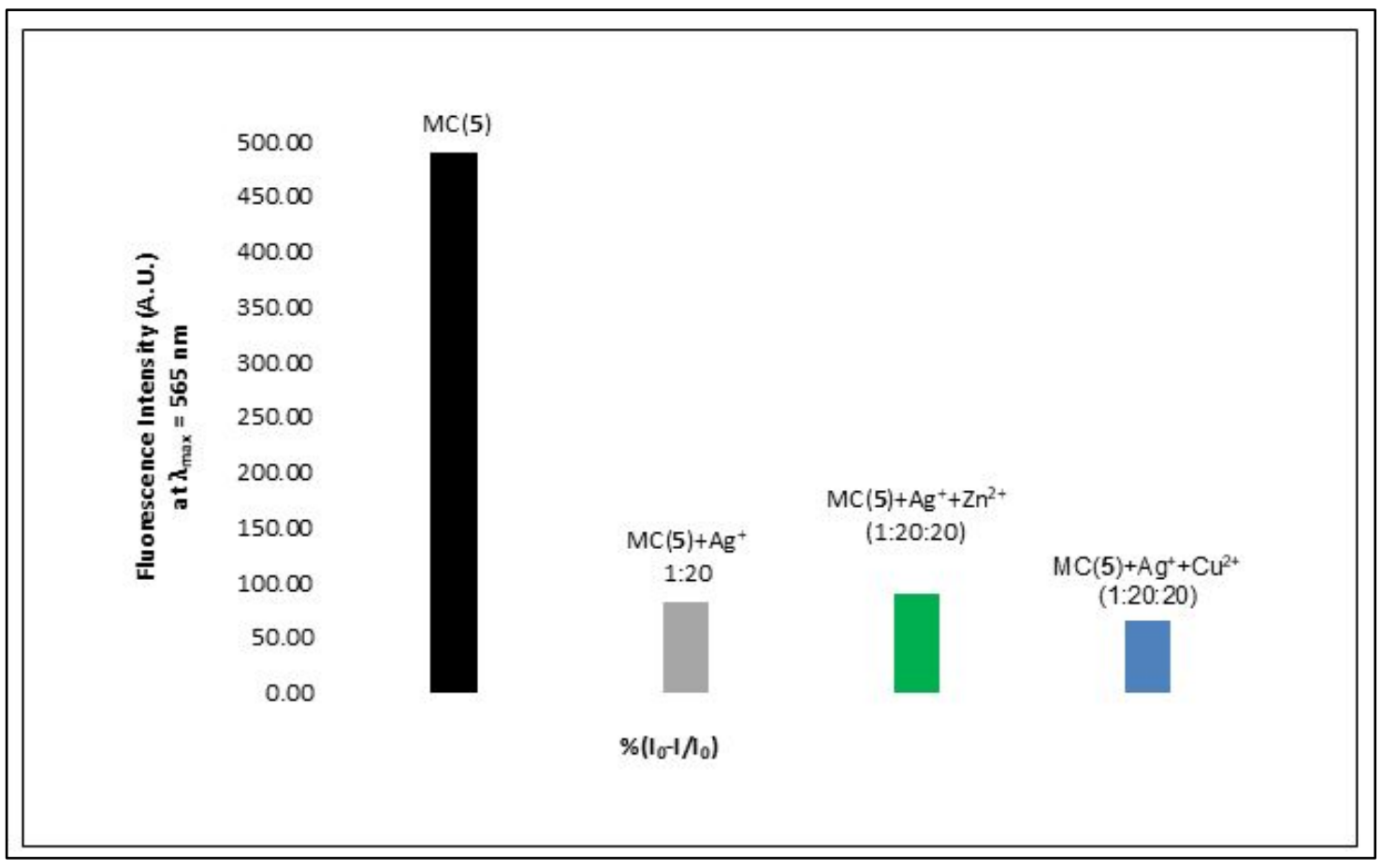

Figure S66. Competitive binding of the macrocycle **5** in presence of Ag^+ plus Zn^{2+} and Cu^{2+} as the two other cation which provide also fluorescence quenching.
Molar ratios: **5** = 11 µM and **5**:Ag^+:Zn^{2+} or **5**:Ag^+:Cu^{2+} = 1:20:20 equivalents.

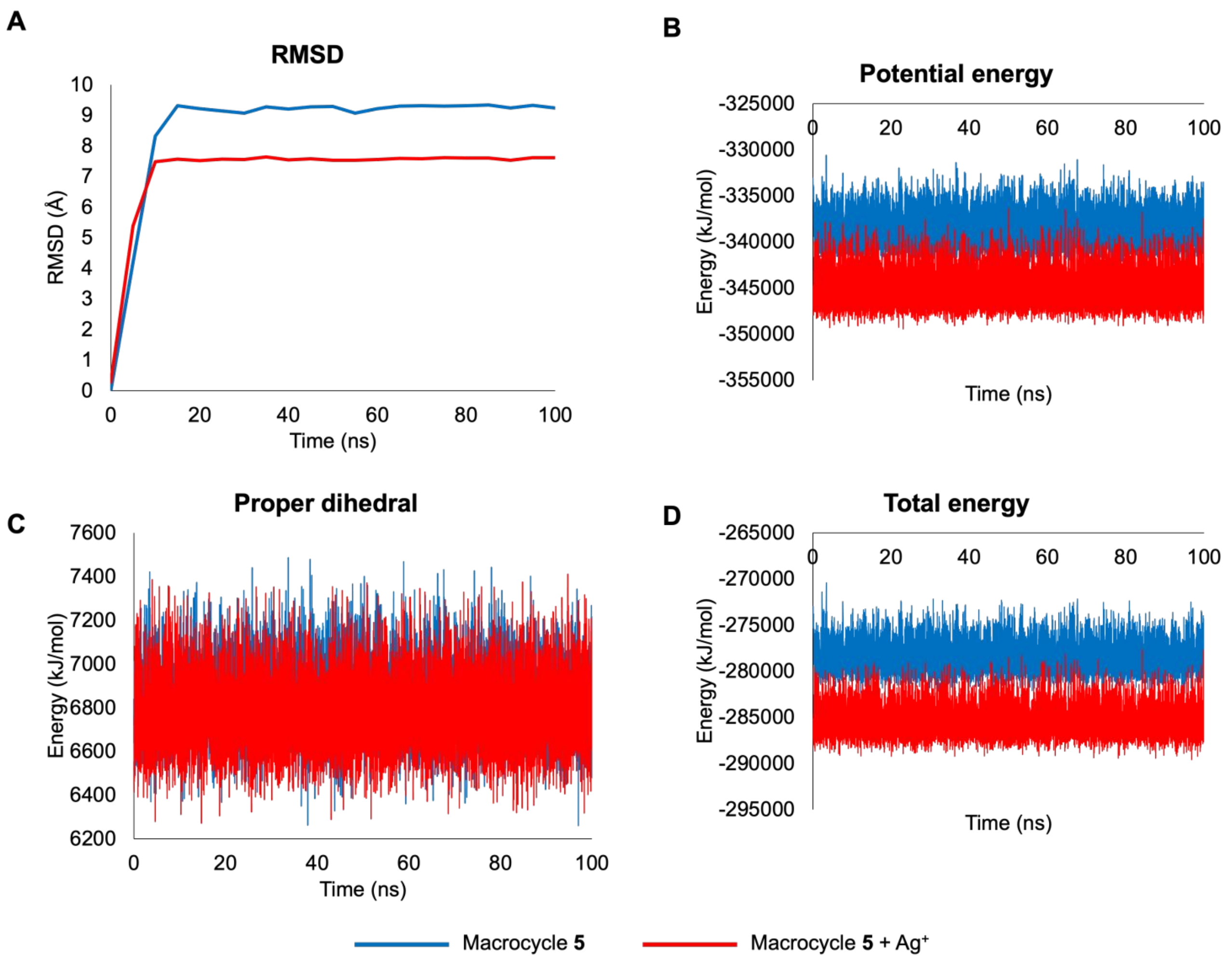

Figure S67. Quantitative metrics for MD simulations of free macrocycle 5 (blue line) and macrocycle 5 in complex with Ag^+ cation (red line): **A**) root mean square deviation of atomic position, **B**) potential energy, **C**) proper dihedral and **D**) total energy of the system over the simulation time of 100 ns.

	Macrocycle 5	Macrocycle 10
+ Li (I)	1.0	1.1
+ Na (I)	0.8	0.8
+ K (I)	0.8	1.0
+ Ag (I)	0.6	0.4
+ Ca (II)	1.1	1.0
+ Mg (II)	1.1	0.8
+ Zn (II)	0.9	0.9
+Cu (II)	0.7	0.6
+Ni (II)	1.5	1.1
+Co (II)	1.6	1.1

Table S5. Relative fluorescence quantum yield calculated for Macrocycle 5 and macrocycle 10 in the presence of the reported cations.

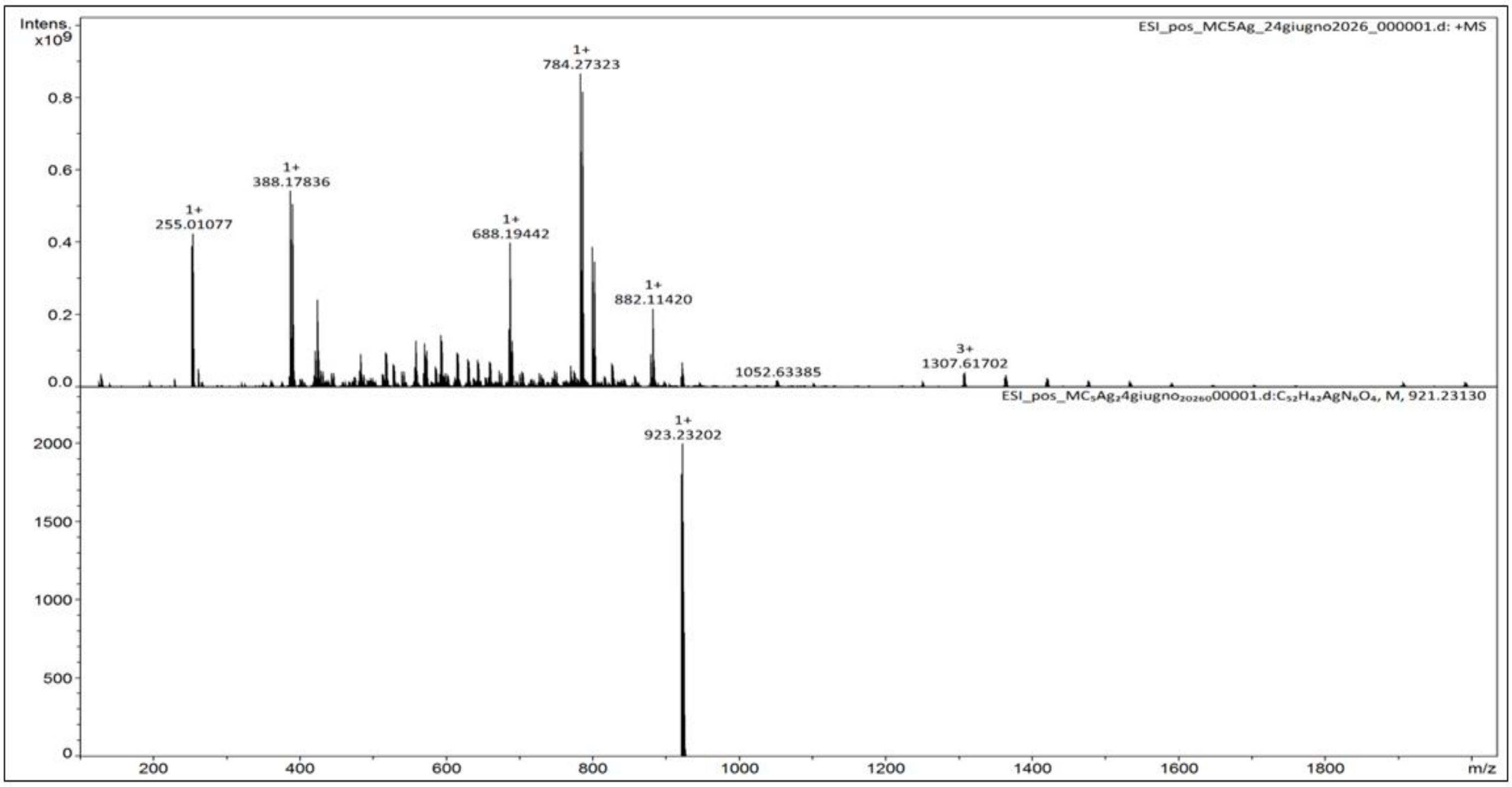

Figure S68. ESI-MS spectrum of the complex between 5 and Ag^+; expected m/z = 923.23202.

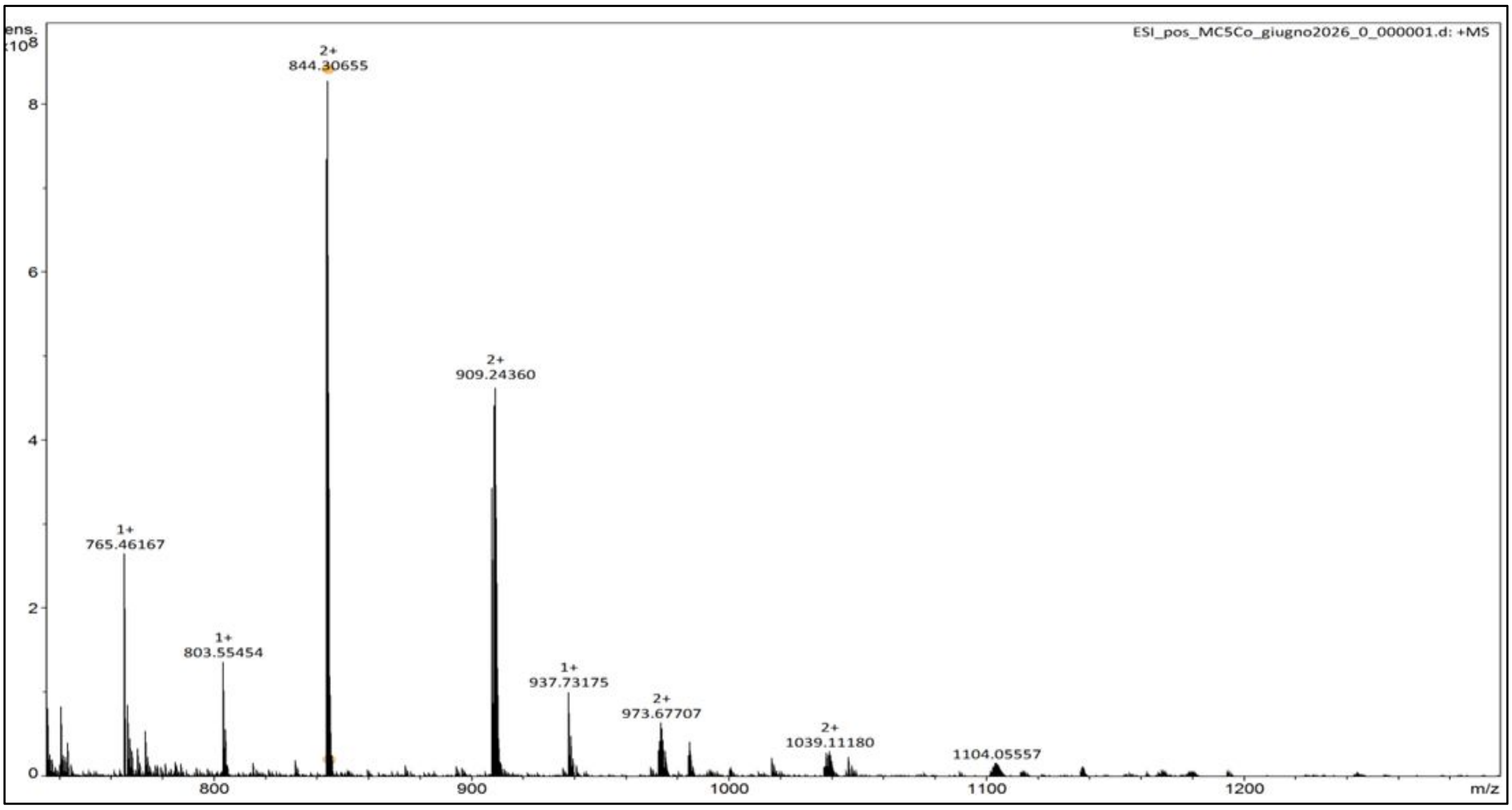

Figure S69. ESI-MS spectrum of the complex between 5 and Co^{2+}; Found m/2z = 844.30655 for [M-Et+Co]2+.

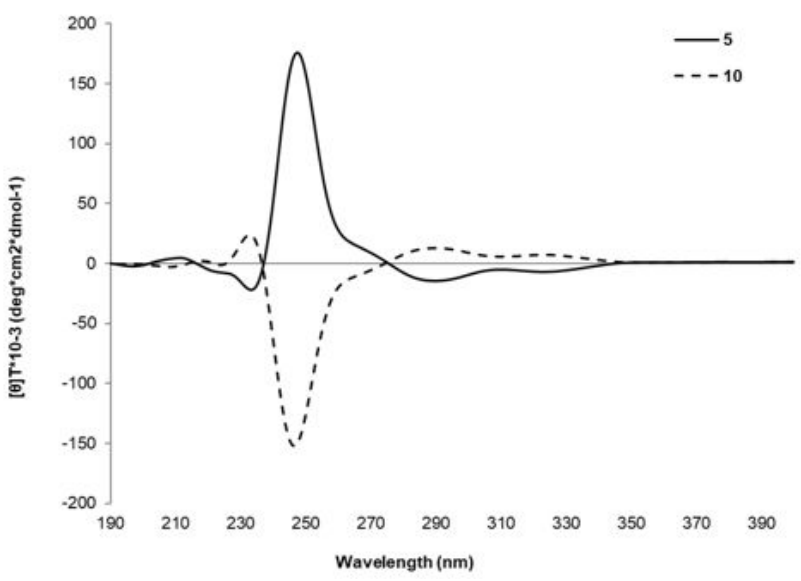

Figure S70. CD spectra of the macrocycles **5** (solid line) and **10** (dashed line) in MeOH/DMSO, *v/v*, 99/1 (200 μM).

Table S6. Comparison of the sensing performance of macrocycles **5** and **10** with representative BINOL/triazole-based fluorescent chemosensors reported in the literature.

Sensor	Target ion	Fluorescence response	Notes	Ref.
Macrocycle 5	Ag+	Turn-off (75.2% quenching)	Highest response among tested ions; Ka = 3.14×10^3 M^{-1}	-
Macrocycle 5	Co^{2+}	Turn-on (73.5% enhancement)	Dual sensing behavior	-
Macrocycle 10	Ag+	Turn-off (60.5% quenching)	Lower affinity respect to macrocycle 5 due to open conformation	-
BINOL-bis(triazole) cyclophane	Ag+	Turn-off	Highly selective for Ag+	[14]
BINOL-triazole derivative	Ag+	Turn-on	Molecular switch behavior	[26]
BINOL-triazole-pyrene	Ag+	Ratiometric enhancement	Selective Ag+ sensing	[26]
BINOL-bis(triazole)-glucose	Ag+	Turn-off	High selectivity toward Ag+	[1]

(1) Vanommeslaeghe, K.; MacKerell Jr, A. D. *Journal of chemical information modeling* **2012**, *52*, 3144.
(2) Vanommeslaeghe, K.; Raman, E. P.; MacKerell Jr, A. D. *Journal of chemical information modeling* **2012**, *52*, 3155.
(3) Huang, J.; Rauscher, S.; Nawrocki, G.; Ran, T.; Feig, M.; de Groot, B. L.; Grubmüller, H.; MacKerell, A. D. *Biophysical Journal* **2017**, *112*, 175a.

(4) Mansfeld, F. M.;Feng, G. and Otto, S. *Org. Biomol. Chem.* **2009**, 7, 4289–4295.
